# Guanxinkang Decoction Attenuates the Inflammation in Atherosclerosis by Regulating Efferocytosis and MAPKs Signaling Pathway in LDLR^−/−^ Mice and RAW264.7 Cells

**DOI:** 10.3389/fphar.2021.731769

**Published:** 2021-12-07

**Authors:** Yifan Zhang, Jie Ding, Yiru Wang, Xiaoteng Feng, Min Du, Ping Liu

**Affiliations:** Department of Cardiology, Longhua Hospital, Shanghai University of Traditional Chinese Medicine, Shanghai, China

**Keywords:** guanxinkang decoction, atherosclerosis, inflammation, efferocytosis, MAPKs

## Abstract

Guanxinkang decoction (GXK), a traditional Chinese medicinal drug, is used to treat cardiovascular disease. The aim of the study was to investigate the effects of GXK on inflammation in LDLR^−/−^ mice and RAW264.7 cells. Fed with high fat diet for 12 weeks, the mice were randomly divided into six groups, then administered with oral 0.9% saline or GXK (7.24, 14.48, and 28.96 g/kg) or Atorvastatin (1.3 mg/kg) for 12 weeks. RAW 264.7 cells were induced with ox-LDL or ox-LDL plus different concentrations of GXK (1.25, 2.5, and 5 μg/ml), or ox-LDL plus GXK plus MAPKs activators. Serum lipid profiles and inflammatory cytokines were detected by ELISA, gene expression by RT-qPCR, plaque sizes by Oil Red O, α-SMA, caspase 3, NF-κB p65 and TNF-α production by immunofluorescence staining, and protein expression by Western Blot. The phagocytic ability of cells was determined by neutral red uptake assay. Efferocytosis-related proteins (AML, MERTK, TYRO3 and MFGE8) and MAPKs pathways were detected by Western Blot. Compared to mice fed with high fat diet, the mice with GXK showed lower cholesterol, triglyceride, low-density lipoprotein cholesterol, IL-1β, IL-6, and TNF-α, smaller plaque sizes, higher α-SMA, and lower caspase 3 and NF-κB p65 in aortic roots. RAW264.7 cells treated with ox-LDL plus GXK had lower IL-1β, IL-6, and TNF-α. GXK also increased the phagocytic ability of cells. High levels of AML, MERTK, TYRO3 and MFGE8, and decreased levels of iNOS, VCAM-1, LOX-1 and MCP-1, and phosphorylation of ERK1/2, JNK, p38, and NF-κB were detected in GXK-treated group. MAPKs activators reversed the effects of GXK in repressing inflammation and promoting phagocytosis. These results suggested that GXK could attenuate atherosclerosis and resolve inflammation via efferocytosis and MAPKs signaling pathways in LDLR^−/−^ mice and RAW264.7 cells.

## Introduction

Atherosclerosis (AS), a lipid-driven inflammatory disease, lays a pathological basis for cardiovascular diseases (CVDs) (Libby et al., 2019). New pharmacotherapies have been developed in recent decades, but CVDs remain a major cause of death globally, with an estimated annual toll of 17.9 million (World Health Organization, 2021). Lipid-lowering and plaque-stabilizing drugs are the first choice for atherosclerotic disease (Kazi et al., 2017). Different mechanisms are involved in the pathogenesis of AS, including inflammation and efferocytosis.

Inflammation runs through the whole atherosclerotic process, and increases the rate of cardiovascular death (Geovanini and Libby, 19792018). MAPK and NF-κB are critical signaling regulators in inflammation. Lipid accumulation activates MAPK/NF-κB signal pathway (Muslin, 2008). Subsequently, an enormous number of pro-inflammatory cytokines are released, such as TNFα, IL-1β, and IL-6, aggravating inflammation (Mitchell et al., 2016). Therefore, anti-inflammation is a critical strategy in the management of atherosclerotic diseases.

AS is driven not only by inflammation but also by defective efferocytosis. Efferocytosis is a process through which phagocytes clear away apoptotic cells (ACs) (Doran et al., 2020). Effective efferocytosis requires the participation of multiple signaling molecules, such as TAM receptors (Tyro3, Axl, and Mer). These molecules help phagocytes to recognize, capture, phagocytize, and digest ACs, preventing secondary cell necrosis in the plaque and the release of inflammatory factors and toxic molecules from dead cells (8). Studies have found that efferocytosis becomes defective in the advanced stage of AS (Schrijvers et al., 2005), causing secondary necrosis, inflammation, and vulnerable plaques (Tabas, 2010). In AS, inflammation resolves with the clearance of ACs, providing new insight into the prevention and treatment of cardiovascular diseases.

AS has long been prevented and treated with traditional Chinese medicine (TCM) (Hao et al., 2017; Layne and Ferro, 2017). According to the theory of TCM, AS arises from “phlegm and blood stasis”, and should be treated with blood-activating and phlegm-resolving drugs. Previous studies suggested that some Chinese herbs with blood-activating and phlegm-resolving properties could exert therapeutic effects, such as suppressing inflammation in hypertrophied hearts of atherosclerosis-prone mice, resisting angiogenesis in zebrafish, and inhibiting oxidative stress in atherosclerotic ApoE^−/−^ mice (Liu et al., 2017; Xiao et al., 2018; Hu et al., 2019).

Guanxinkang decoction (GXK) is based on Gualou Xiebai Banxia decoction (GXBD), one classical TCM formula for coronary heart diseases (Lin et al., 2021). Guo et al. found that GXBD was capable of modulating blood lipid and inflammation in the AS model of Apo-E^−/−^ mice (Guo et al., 2017). Our previous research showed that GXK alleviated lipid metabolism disorder and inhibited the inflammatory response of ApoE^−/−^ mice (Mao et al., 2012). However, the underlying mechanism is not yet clear. Hence, we designed this study to explore the anti-inflammatory effects of GXK on AS in LDLR^−/−^ mice and RAW264.7 cells.

## Methods

### GXK Materials

GXK containing six herbs (Table 1) was offered by the Pharmacy of Longhua Hospital Affiliated to Shanghai University of Traditional Chinese Medicine. All the herbs were authenticated by a pharmacognosist of Longhua Hospital Affiliated to Shanghai University of Traditional Chinese Medicine, according to the Chinese Pharmacopoeia (Version 2015). All the herbs were soaked with double distilled water (ddH_2_O) and decocted twice. Then the mixed liquid was concentrated to 100 ml in a rotary steamer. Finally, lyophilized powder was collected into a lyophilizer. Then the powder was dissolved in 0.9% saline for oral administration in mice. And the powder was also resolved in Dulbecco’s Modified Eagle Medium supplemented with 10% fetal bovine serum for cell induction. Liquid Chromatography Mass Spectrometry (LCMS) of GXK was conducted (data not shown, Supplementary Material).

**TABLE 1:** Components of GXK.

Chinese name	Name for publishing^a^	Amount (g)	Lot no.	Place of origin	Company
Huang Qi	*Astragalus mongholicus* Bunge, root	30	2020020308	Neimenggu, China	Shanghai Yanghetang Chinese Medicine Decoction Pieces Co., Ltd.
Yi Mu Cao	*Leonurus japonicus* Houtt., aerial part	30	20200219-1	Henan, China	Shanghai Wanshicheng Pharmaceutical Co., Ltd.
Dan Shen	*Salvia miltiorrhiza* Bunge, root	12	200212	Shandong, China	Shanghai Hongqiao traditional Chinese medicine decoction pieces Co., Ltd.
Xie Bai	*Allium chinense* G.Don, bulb	12	2020021903	Shanxi, China	Shanghai Dehua Chinese Medicine Products Co., Ltd.
Ban Xia	*Pinellia ternata (Thunb.)* Makino., tuber	12	20200123	Gansu, China	Shanghai Yanghetang Chinese Medicine Decoction Pieces Co., Ltd.
Gua Lou	*Trichosanthes kirilowii* Maxim., mature fruits	15	20200227-1	Hebei, China	Shanghai Wanshicheng Pharmaceutical Co., Ltd.

a Latin scientific name + plant part (s).

### Animals

The animal study protocol was approved by the ethics committee of Longhua Hospital Affiliated to Shanghai University of Traditional Chinese Medicine (No. 2019-N002, Supplementary Material). The LDL receptor deficient mice (LDLR^−/−^, B6/JGpt-Ldlrem1Cd82/Gpt) were purchased from GemPharmatech Co., Ltd. (Nanjing, Jiangsu, http://www.gempharmatech.com) and bred at the Animal Center of Longhua Hospital Affiliated to Shanghai University of Traditional Chinese Medicine.

Forty-eight LDLR^−/−^ mice were randomly divided into six groups (8 mice/group): control group (C), model group (M), low GXK group (GL, 7.24 g/kg), medium GXK group (GM, 14.48 g/kg), high GXK group (GH, 28.96 g/kg), and 1.3 mg/kg Atorvastatin group (Ato). When the mice were 7 weeks old, they were given the high fat diet (HFD, 21% fat and 0.15% cholesterol). After 12 weeks’ feeding with HFD the mice were orally administered with GXK or Atorvastatin for 12 weeks. Model group received the same volume of 0.9% saline. Meanwhile, control group was fed with normal diet and orally administered with 0.9% saline. The health of the mice was monitored by observing the changes in the weight, posture, mental state, and excrement. The mice were fasted 12 h before sacrifice via chloral hydrate anesthesia.

### Cell Culture

RAW264.7, a mouse macrophage cell line, was purchased from Shanghai Cell Bank Type Culture Collection Committee (Shanghai, China). It was cultured in DMEM (SH30022.01, HyClone, United States) containing 10% fetal bovine serum (10099141C, Gibco, United States), 100 U/ml penicillin, 100 μg/ml streptomycin (SV30010, HyClone, United States) at 37°C in a humidified incubator containing 5% CO_2_.

Oxidized LDL (ox-LDL) was chosen as inducer because it can activate endothelial cells and intimal macrophages at atherosclerotic lesions to release pro-inflammatory cytokines, chemokines and adhesion molecules (Rhoads and Major, 2018). Monocyte-derived macrophages recognize and ingest ox-LDL to generate foam cells, significant markers of unstable atherosclerotic plaques (Bobryshev et al., 2016). Therefore, ox-LDL-induced RAW 264.7 cells were used in the *in vitro* experimental model.

### Evaluation of Serum Lipid in the LDLR^−/−^ Mice

Blood samples were kept for 0.5 h at room temperature and then centrifuged for 20 min at 3,000 rpm. The upper transparent serum was collected and diluted with phosphate-buffered saline (PBS) (1:2). Total cholesterol (TC), triglyceride (TG), low-density lipoprotein cholesterol (LDL-C), aspartate aminotransferase (AST), and alanine aminotransferase (ALT) levels were analyzed by automatic MODULAR biochemical identification instrument in the Clinical Laboratory Department of Longhua Hospital Affiliated to Shanghai University of Traditional Chinese Medicine.

### Oil Red O Staining for Mice Aortic Sinus Lipid-Rich Plaque

The fresh heart tissues were soaked in 4% paraformaldehyde for fixation at least 48 h, then transferred into in 10, 20, and 30% sucrose solutions for dehydration, and finally embedded with OCT glue. The heart was sliced into 10-µm-thick cross-sections by frozen tissue slicer to reveal the atrium and aortic sinus. Frozen slices were stained with Oil Red O solution (0.5% in isopropanol, diluted with ddH2O at a ratio of 3:2) at 37°Cfor 2 h and counterstained with hematoxylin for 2 min. ImageJ Software was used to quantify the Oil Red O stained lipid-rich plaque areas. The percentage of positive staining in total intimal area was used for data statistics.

### ELISA for Quantitative Analysis of IL-1β, IL-6, and TNF-α

The blood serum of mice and culture supernatant of RAW264.7 cells were collected. The levels of interleukin (IL)- 1β, IL-6, and tumor necrosis factor (TNF)-α were measured by Enzyme-linked immunosorbent assay (ELISA) kits (Beyotime, China) according to the manufacturer’s instructions. Mice blood serum was obtained as described above. Culture supernatant of cells was derived as follows: RAW264.7 cells (4 × 105 cells/well) in 96-well plates were treated with various concentrations of GXK and 40 μg/ml ox-LDL for 24 h. Or cells were pretreated with asiatic acid, anisomycin and LM22B-10 (Sellect, CN) for 0.5 h, then treated with ox-LDL for 24 h.

### Immunofluorescence Staining

Frozen slices of aortic sinus were made as mentioned before in 2.5 Oil Red O staining for mice aortic Sinus lipid-rich plaque. On the first day, frozen slices were penetrated with 0.1% Triton X-100 for 15 min. After being washed for two times with PBS, the slices were incubated with 5% bovine serum albumin (BSA) in PBS with Tween-20 (PBST) for 1 h, then with anti-α-SMA (Abcam, United Kingdom), anti-caspase 3 (CST, United States) and anti-NF-κB p65 (SANTA CRUZ, United States) at 4°C overnight. On the second day, after being washed twice with PBST, the slices were incubated with goat anti-rabbit IgG H&L (AlexaFluor^®^ 488 CST, United States) or anti-mouse IgG Fab2(AlexaFluor^®^ 488 CST, United States) at room temperature for 1 h, then treated with 4′,6-diamindino-2-phenylindole (DAPI).

RAW264.7 cells were cultured and treated as indicated, then washed for three times with PBS. The cells were fixed with 4% paraformaldehyde for 15 min and penetrated with 0.1% Triton X-100 for 10 min. The cells were blocked with PBS containing 5% bovine serum albumin (BSA) for 1 h, then with anti- TNF-α (CST, United States) at 4°C overnight. On the following day, the cells were washed with PBS and incubated with goat anti-rabbit IgG H&L (Alexa Fluor^®^ 488, CST, United States of America) at room temperature for 1 h. The cells were treated with DAPI. All images were captured with a fluorescence microscope.

### Real-Time Quantitative PCR Assay

The mRNA levels of inflammatory factors (IL- 1β, IL-6, and TNF-α) and efferocytosis related molecules (AXL, MERTK, and TYRO3) within the mice aorta and cells were tested. RAW264.7 cells were cultured and treated as indicated. Total RNA was extracted using RNA Purification Kit (EZBioscience, CN) with a spin column according to the manufacturer’s protocol. Isolated RNA was reverse-transcribed into cDNA using PrimeScript RT Reagent Kit (TAKARA, China) following the standard protocol. The Real-Time Quantitative PCR (RT-qPCR) assay was conducted using TB Green Premix EX Taq (TAKARA, China) with the Applied Biosystems 7500 Real-Time PCR System (Applied Biosystems, United States). The amplification parameters were 95°C for 1 min, followed by 40 cycles of 95°C for 5 s and 58°C for 15 s, 72°C for 30 s, and 95°C for 15 s. The relative expression of mRNA was calculated after normalization to β-actin. All primer sequences used are listed in Table 2.

**TABLE 2:** List of primers for real-time qPCR analysis.

Gene	Oligonucleotide sequence	
β-Actin	Forward	5′- GAG​ACC​TTC​AAC​ACC​CCA​GC - 3′
	Reverse	5′- ATG​TCA​CGC​ACG​ATT​TCC​C- 3′
IL-1β	Forward	5′- GCT​TCA​GGC​AGG​CAG​TAT​CA- 3′
	Reverse	5′- TGC​AGT​TGC​TAA​TGG​GAA​CG- 3′
IL-6	Forward	5′-AAA​GCA​GCA​AAG​AGG​CAC​TG- 3′
	Reverse	5′-TAC​CTC​AAA​CTC​CAA​AAG​ACC​AG- 3′
TNF-α	Forward	5′- CCC​TCC​AGA​AAA​GAC​ACC​ATG- 3′
	Reverse	5′- CAC​CCC​GAA​GTT​CAG​TAG​ACA​G- 3′
AXL	Forward	5′- GAGCCAACCGTGGAAAGA - 3′
	Reverse	5′- AGG​CCA​CCT​TAT​GCC​GAT​CTA - 3′
MERTK	Forward	5′- AGT​TTG​GGA​CGT​TGG​TGG​AT - 3′
	Reverse	5′- GGA​CAC​CGT​CAG​TCC​TTT​GT - 3′
TYRO3	Forward	5′- ACT​GGC​TTC​TCT​GCT​GCT​C - 3′
	Reverse	5′- AGC​ATC​AGA​CCG​TTC​CAC​TG - 3′

### Western Blotting

Mouse aortas or cultured cells were lysed with radio immunoprecipitation assay (RIPA) lysis buffer. The lysates were incubated on ice for 30 min for full dissociation and centrifuged at 12,000 rpm for 15 min at 4°C, and the supernatants were collected. The protein concentration was determined using a bicinchoninic acid (BCA) protein assay kit (Beyotime, China). Proteins (30 μg) were separated by 10% sodium dodecyl sulphate-polyacrylamide gel electrophoresis (SDS-PAGE) and transferred to polyvinylidene fluoride (PVDF) membranes for 60 min at 350 mA. After being blocked with 5% BSA in Tris-buffered saline with Tween-20 (TBST) for 1 h, the membranes were incubated overnight with primary antibodies diluted using 5% BSA in TBST at 4°C. On the next day, the membrane was washed with TBST buffer, and incubated for 1 h with the appropriate secondary antibody. After washing the membrane with TBST buffer, the protein bands were detected with enhanced chemiluminescence (ECL) western blot kit (Beyotime, China), visualized and photographed using the ChemiScope 6000 imaging machine (CLINX, China). Finally, digital images were analyzed using ImageJ Software to obtain the grey-scale value of signals. β-actin was used as an internal reference.

### Neutral Red Uptake Assay

The effects of GXK on the phagocytic ability of cells was determined using Neutral red (NR) uptake assay (Neutral Red Cell Proliferation and Cytotoxicity Assay Kit, Beyotime, China), following the manufacturer’s instructions. RAW264.7 cells (4 × 104 cells/well) in 96-well plates were treated with various concentrations of GXK and 40 μg/ml ox-LDL for 24 h. Meanwhile, the cells were pretreated with asiatic acid, anisomycin and LM22B-10 (Sellect, CN) for 0.5 h, then treated with ox-LDL for 24 h. Then NR staining solution was added in each well and incubated for 2 h. The cells were washed for three times with PBS. 200 μl cell lysis solution was added in cell plates and measured on a microplate reader at a wavelength of 540 nm.

### Statistical Analysis

Data from three independent experiments were shown as the mean ± standard deviation (SD), and processed with one-way analysis of variance (ANOVA) with SPSS 21.0. *p* < 0.05 was considered statistically significant.

## Results

### GXK Attenuates Atherosclerosis in Mice

The mice in the model, GXK, and Ato groups had much higher body weights than those in the C group after 12 weeks of HFD (*p* < 0.05); while after 12 weeks’ intervention of GXK or Ato, the body weight of mice was significantly lower than those of the model group (*p* < 0.05, Supplementary Figure S9). And the intervention of GXK or Ato also attenuates liver enzymes levels in mice (*p* < 0.05, Supplementary Figure S10).

**FIGURE 1:**
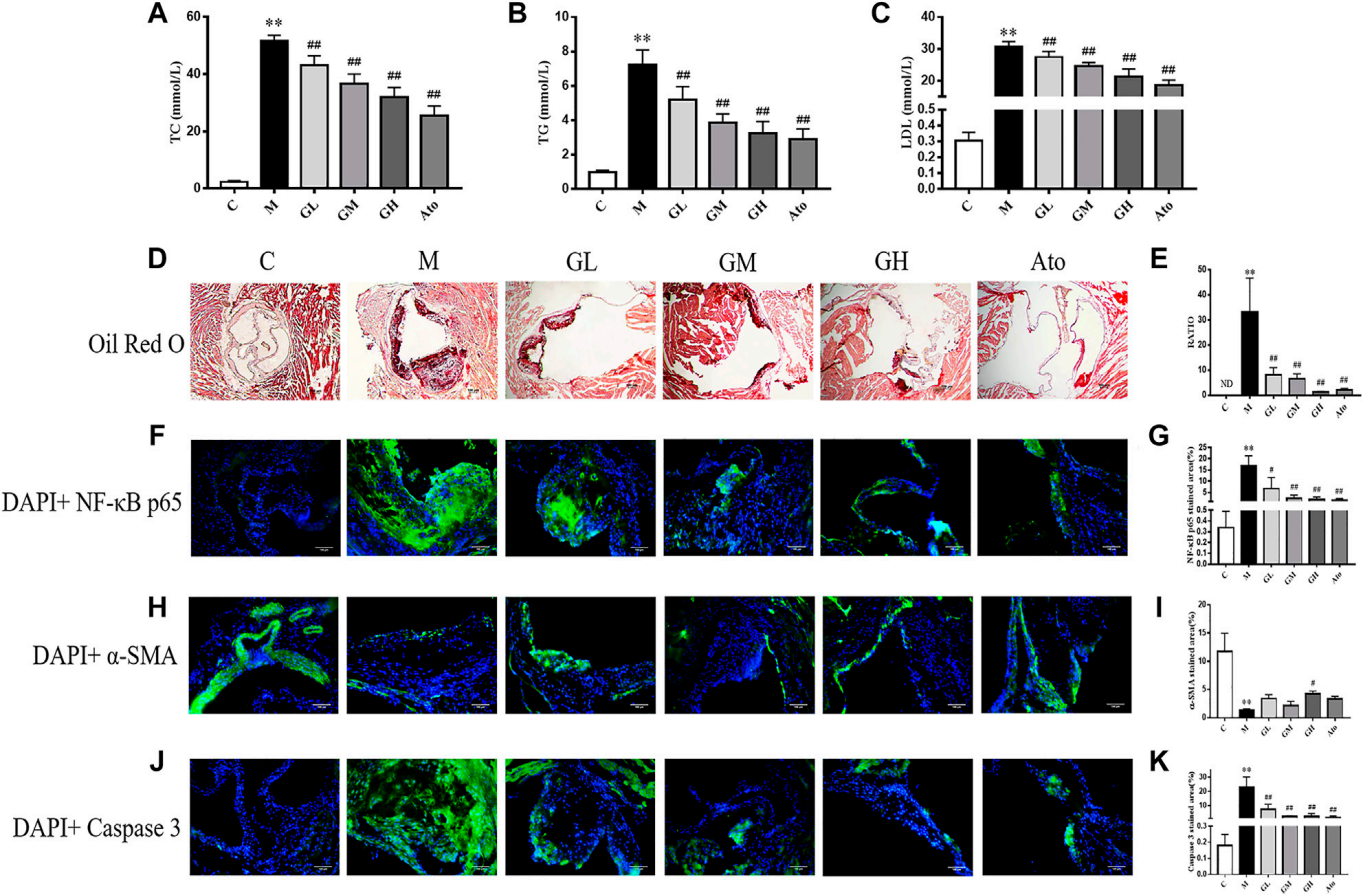
Serum lipid profiles and atherosclerosis of aortic sinus in the LDLR^−/−^ mice. **(A–C)** TC, TG, and LDL levels of serum were detected, n = 8. **(D)** Oil Red O staining was used to detect the progress of lipid-rich plaques, n = 3. **(E)** The areas of lesion were calculated respectively by ImageJ analysis software. (F–K) Representative photomicrographs of aortic root sections stained with α-SMA **(F)**, caspase 3 **(H)**, and NF-κB P65 **(J)** in atherosclerotic plaque followed by the quantification **(G,I,K)**, n = 3. C means control group, M means model group, GL means low GXK group, GM means medium GXK group, GH means high GXK group, Ato means Atorvastatin group. TC, total cholesterol; TG, triglyceride; LDL, low density lipoprotein cholesterol; ND, not detected; RATIO = plaque area/aortic sinus lumen area ×100%. M group was compared to C group and GXK or Ato group was compared with M group. Data are expressed as mean ± SD. **p < 0.01 vs. C group; ##*p* < 0.01 vs. M group.

To explore the effect of GXK on blood lipids in LDLR^−/−^ mice, the serum levels of total cholesterol (TC), triglyceride (TG), and low-density lipoprotein cholesterol (LDL-C) were quantified. The mice in the model group had much higher levels of TC, TG and LDL-C than the control group (*p* < 0.01); while after 12 weeks’ intervention of GXK or Ato, all the three indexes were lowered (*p* < 0.01, Figures 1A–C). The above results showed that GXK could regulate lipid metabolism in AS model mice.

The morphology of lipid-rich plaques was assessed by Oil Red O staining. As shown in Figure 1D, lipid-rich plaques were significantly larger in the model group, but not detected in the control group (*p* < 0.01). In contrast, a significant reduction of atherosclerotic lesion area was detected in the GXK-treated and Ato-treated groups than in the model group (*p* < 0.01, Figure 1E). The above results suggested that GXK could reduce atherosclerotic plaques in HFD-induced-LDLR^−/−^ mice.

In order to further explore the effect of GXK on the inflammation and stability of atherosclerotic plaques, immunofluorescence staining was performed. As shown in Figures 1F,G, the expression level of NF-κB p65 was significantly increased in the atherosclerotic lesions of the model group (*p* < 0.01). GXK and Ato inhibited the increase of NF-κB p65 (*p* < 0.05). Then we determined whether the enhanced inflammation affected atherosclerotic plaque stability. High necrotic core size and low fibrous cap area can increase plaque vulnerability. Figures 1H–K showed that the expression level of α-SMA was significantly decreased and the expression level of caspase 3 was significantly increased in the atherosclerotic lesions of the model group (*p* < 0.01). High dose of GXK increased α-SMA expression (*p* < 0.05). GXK and Ato could decrease caspase 3 expression (*p* < 0.05). The above results suggested that GXK could improve the stability of the plaque.

### GXK Attenuates Inflammatory Cytokine Expression in Mice

**FIGURE 2:**
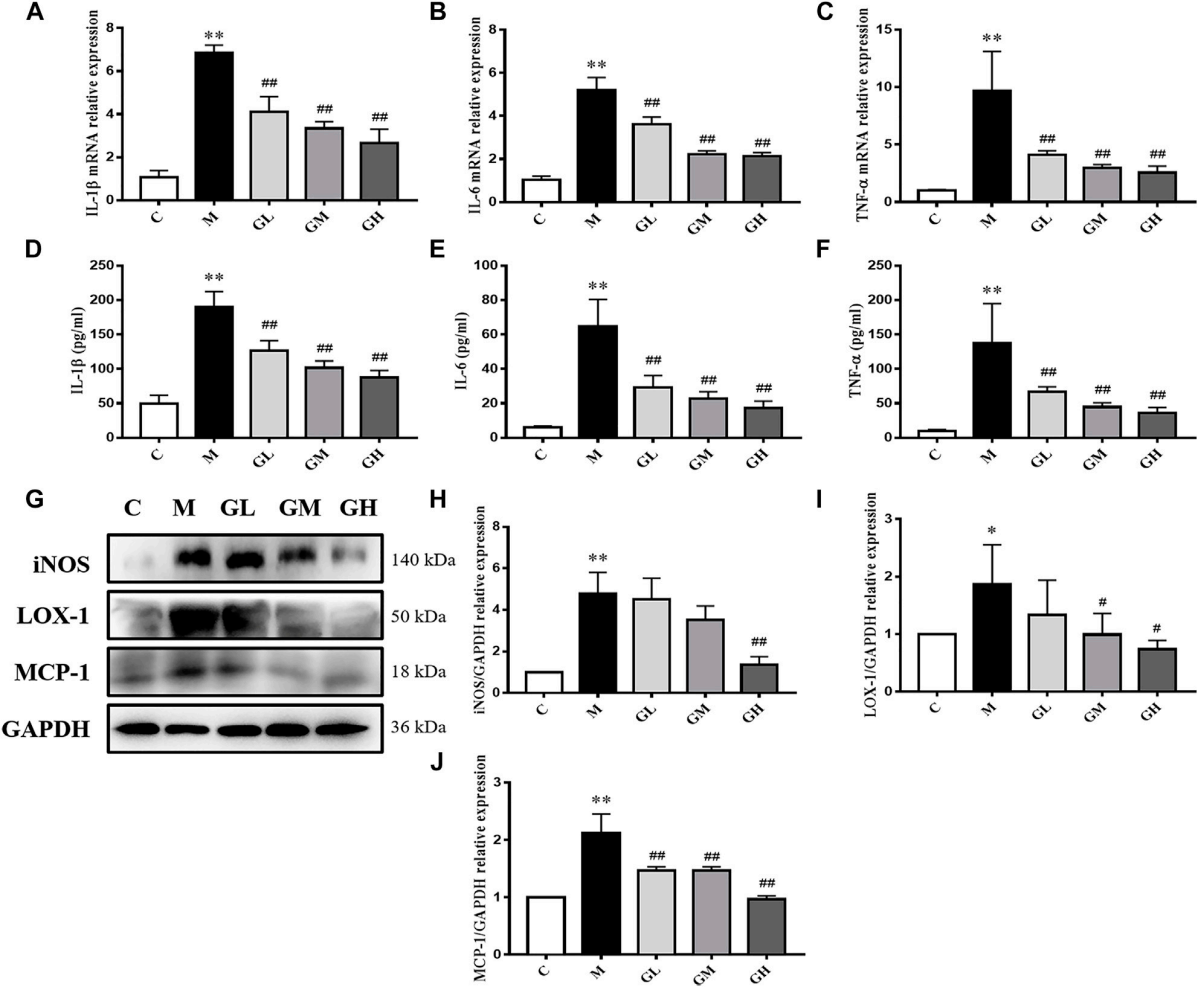
Inflammatory cytokines in the LDLR^−/−^ mice. **(A–C)** Aorta gene expression of IL-1β, IL-6 and TNF-α were detected by RT-qPCR method, n = 8. **(D–F)** Production of IL-1β, IL-6 and TNF-α in plasma were measured by ELISA kit, n = 8. **(G)** Protein was extracted from mice aorta. Then the levels of iNOS, LOX-1 and MCP-1 were determined by western blotting assay, n = 3. **(H–J)** The quantitative results were depicted. C means control group, M means model group, GL means low GXK group, GM means medium GXK group, GH means high GXK group. M group was compared to C group and GXK was compared with M group. Data are expressed as mean ± SD. *p < 0.05, **p < 0.01 vs. C group; #*p* < 0.01, ##*p* < 0.01 vs. M group.

In order to further examine the influence of GXK on inflammation, the expression of inflammatory factors of the aorta were detected. The mRNA levels of inflammatory cytokines (IL-1β, IL-6, and TNF-α) in mouse aortas were detected by RT-qPCR. Expression of mRNA was normalized to the housekeeping gene β-Actin. As shown in Figures 2A–C, the mRNA levels of IL-1β, IL-6 and TNF-α in the model group increased compared with those in the control group (*p* < 0.01). The 12 weeks’ intervention of GXK significantly decreased the mRNA levels of IL-1β, IL-6 and TNF-αexpression (*p* < 0.01). These results were consistent with the changes in the plasma levels of inflammatory cytokines IL-1β, IL-6, and TNF-α, as revealed by ELISA (*p* < 0.01, Figures 2D–F). Then the protein levels of inducible nitric oxide synthase (iNOS), lectin-like oxidized low-density lipoprotein receptor-1 (LOX-1) and monocyte chemotactic protein-1 (MCP-1) were detected by Western Blot assays. The protein levels of iNOS, LOX-1, and MCP-1 were significantly increased in the model group, compared to the control group (*p* < 0.05). GXK intervention decreased the expression levels of these proteins, especially high-dose GXK had a significant effect (*p* < 0.05, Figures 2G,H). The above results showed that GXK could inhibit the inflammatory response in atherosclerotic mice through inflammatory factors.

### GXK Enhances Efferocytosis in Mice

**FIGURE 3:**
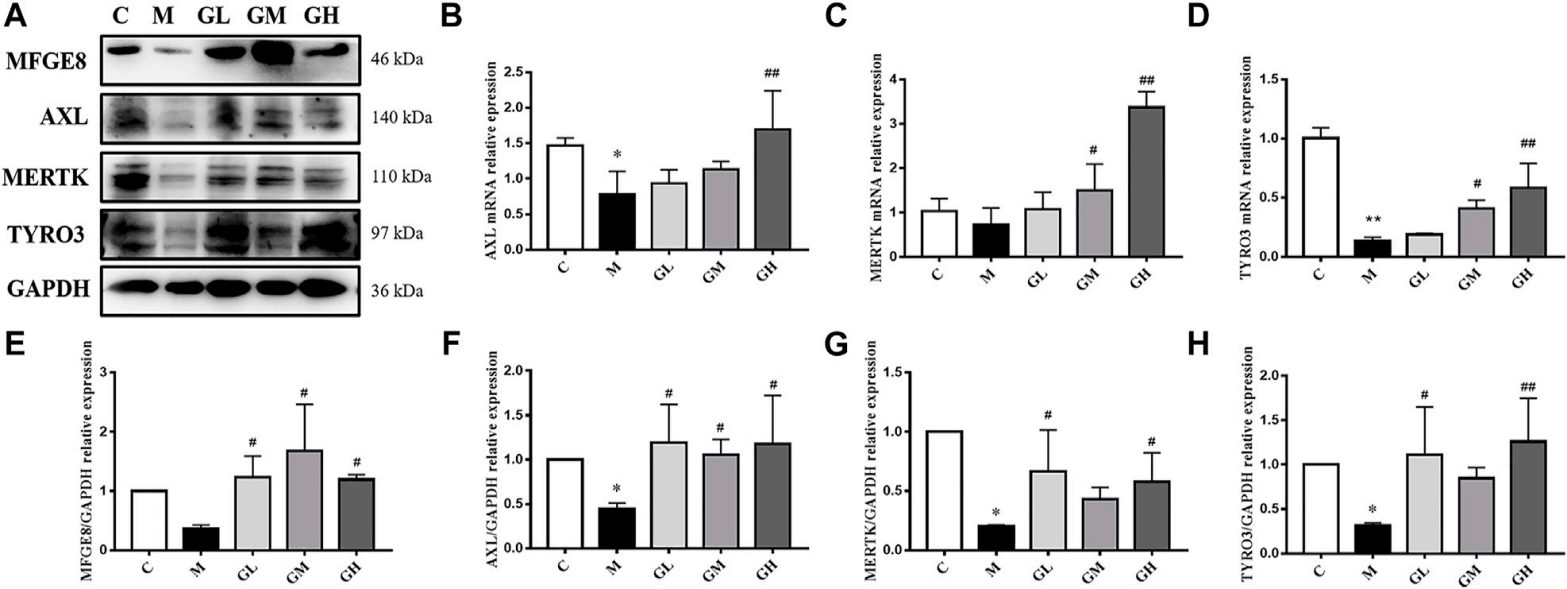
Effects of GXK on efferocytosis related molecules in the LDLR^−/−^ mice. **(A)** Protein was extracted from mice aorta. Then AXL, MERTK, TYRO3 and MFGE8 were tested by western blotting assay, n = 3. **(B–D)** Aorta gene expression of AXL, MERTK and TYRO3 were detected by RT-qPCR method, n = 3. **(E–H)** The quantitative results of western blotting assay were depicted. C means control group, M means model group, GL means low GXK group, GM means medium GXK group, GH means high GXK group. MFGE8, milk fat globule-epidermal growth factor. M group was compared to C group and GXK was compared with M group. Data are expressed as mean ± SD. *p < 0.05, **p < 0.01 vs. C group; #*p* < 0.05, ##*p* < 0.01 vs. M group.

Efferocytosis plays a critical role in the progression of AS and is related to inflammation and plaque stability. Thus, we detected the effect of GXK on efferocytosis. The mRNA levels of AXL, MERTK and TYRO3 in mouse aortas were detected by RT-qPCR method. As shown in Figures 3B–D, the mRNA levels of AXL and TYRO3 in the model group decreased, compared with those in the control group (*p* < 0.05, *p* < 0.01), but the mRNA level of MERTK expression showed no significant difference. After 12 weeks’ intervention of GXK, the levels of AXL, MERTK, and TYRO3 were significantly increased in the high-dose GXK group (*p* < 0.01). These results were consistent with the changes in protein levels in mouse aortas, as revealed by Western Blot (*p* < 0.05, Figures 3A, F–H). In addition, GXK also restored the protein expression of bridge molecule MFGE8 to normal (*p* < 0.05, Figure 3E). The results suggested that GXK improved AS through enhancing efferocytosis.

### GXK Inhibits MAPKs-Related Protein Expression in Mice

**FIGURE 4:**
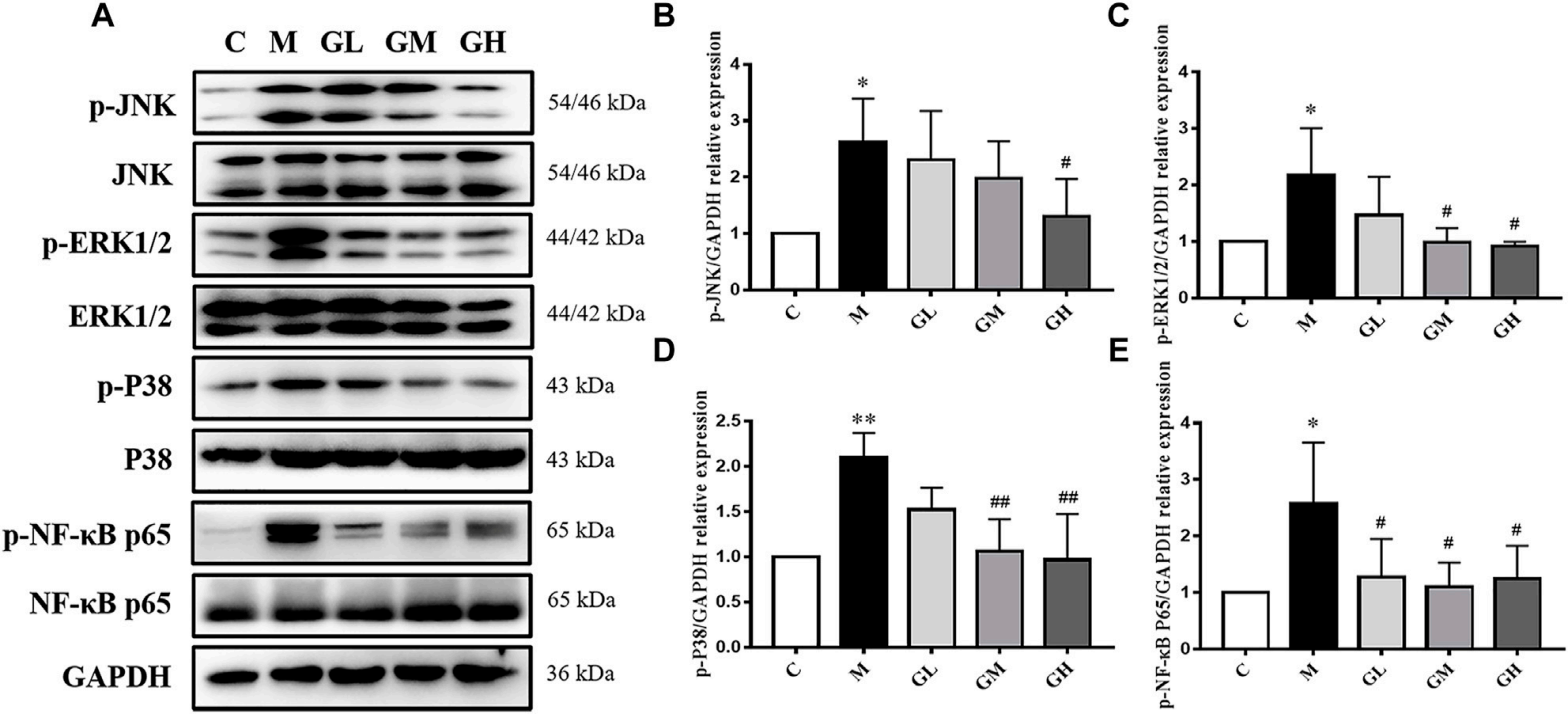
Effects of GXK on MAPKs signaling pathway in the LDLR^−/−^ mice. **(A)** Protein was extracted from mice aorta. Then the total and phosphorylated levels of JNK, ERK1/2, p38 and NF-κB P65 were determined by western blotting assay, n = 3. **(B–E)** The quantitative results were depicted. C means control group, M means model group, GL means low GXK group, GM means medium GXK group, GH means high GXK group. MFGE8, milk fat globule-epidermal growth factor. M group was compared to C group and GXK was compared with M group. Data are expressed as mean ± SD. *p < 0.05, **p < 0.01 vs. C group; #*p* < 0.05, ##*p* < 0.01 vs. M group.

In order to testify whether the increased inflammatory cytokines were related to MAPKs/NF-κB, phosphorylation of p38, JNK, ERK1/2 and NF-κB p65 was examined by Western Blot assays (Figure 4A). The expression levels of MAPKs and NF-κB p65 in the model group were higher than those in the control group (*p* < 0.05), lower than those in GXK groups (*p* < 0.05, Figures 4B–E). These results suggested GXK could mitigate the activation of MAPKs/NF-κB in atherosclerotic mice.

### GXK Attenuates Inflammatory Cytokine Levels in RAW264.7 Cells

**FIGURE 5:**
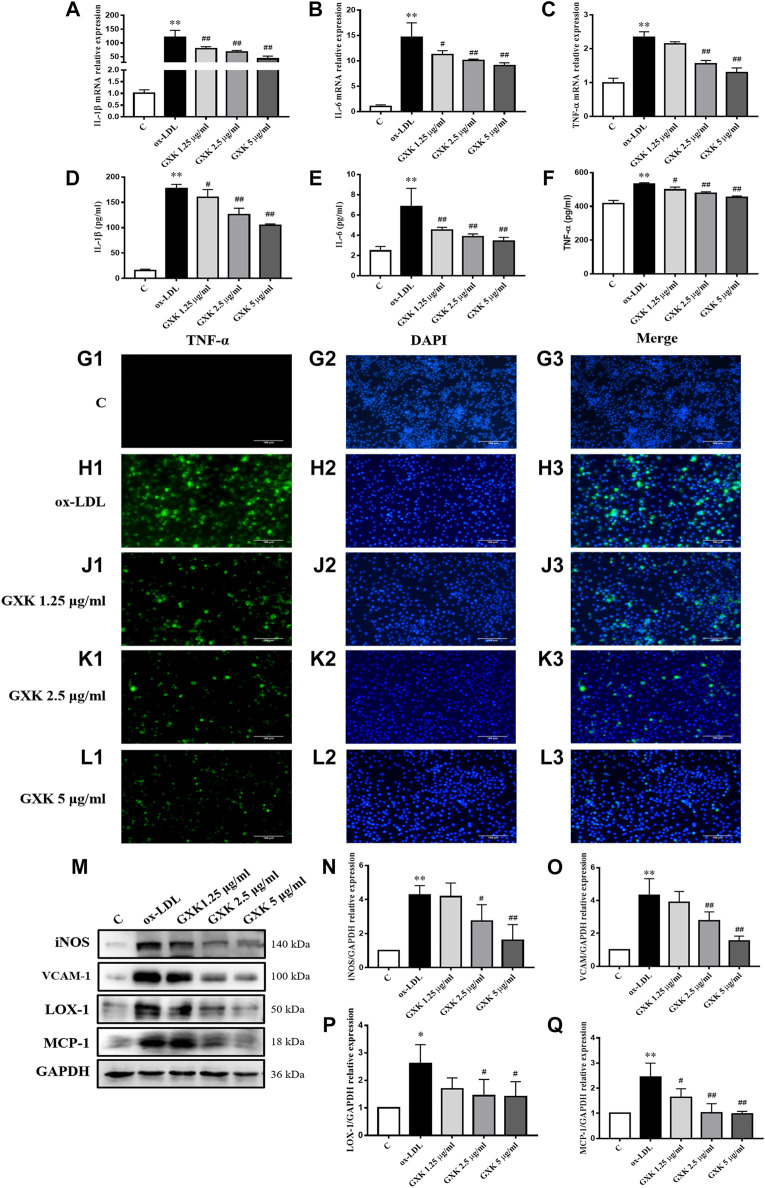
Inflammatory cytokine levels in RAW264.7 cells. **(A–C)** Gene expression of IL-1β, IL-6 and TNF-α was measured by RT-qPCR method and **(D–F)** production of these three cytokines were explored by ELISA kits, n = 3. **(G1–L3)** Immunofluorescence staining method was used to show TNF-α. **(M)** Protein was extracted from RAW264.7 cells. Then iNOS, VCAM-1, LOX-1 and MCP-1 were tested by western blotting assay, n = 3. **(N–Q)** The quantitative results were depicted. C means control group. MFGE8, milk fat globule-epidermal growth factor. Data are expressed as mean ± SD. *p < 0.05, **p < 0.01 vs. C group; #*p* < 0.05, ##*p* < 0.01 vs. ox-LDL-stimulated group.

IIn order to explain the effect and mechanism of GXK, we used ox-LDL-induced RAW264.7 cells as an *in vitro* experimental model. Since 5 μg/ml GXK did not exert significant cytotoxicity in RAW264.7 cells, 1.25, 2.5, and 5 μg/ml concentrations were used for subsequent experiments. RAW 264.7 cells were pretreated with GXK for 0.5 h and then stimulated with ox-LDL for 24 h. The expression levels of IL-1β, IL-6 and TNF-α mRNA in RAW264.7 cell were detected to confirm the results previously observed. As shown in Figures 5A–C, the mRNA levels of IL-1β, IL-6 and TNF-α expression increased in the ox-LDL group (*p* < 0.01). GXK intervention downregulated the mRNA levels of IL-1β, IL-6 and TNF-α expression (*p* < 0.05). And these results were consistent with the changes in protein levels of IL-1β, IL-6 and TNF-α in RAW264.7 cell supernatants, as revealed by ELISA (*p* < 0.05, Figures 5D,E). Moreover, immunofluorescence staining revealed that the TNF-α level in RAW264.7 cells was increased in ox-LDL-stimulated group, compared to those in the control group. After GXK intervention, TNF-α level decreased, suggesting that GXK had anti-inflammatory effects (Figures 5G–L).

We also detected the protein levels of iNOS, vascular cell adhesion molecule-1 (VCAM-1), LOX-1 and MCP-1 by Western Blot assays (Figure 5M). As the results showed, the protein levels of iNOS, VCAM-1, LOX-1 and MCP-1 significantly increased in ox-LDL-stimulated group, compared to those in the control group (*p* < 0.05). GXK intervention decreased the expression levels of those proteins (*p* < 0.05, Figurs 5N–Q). The results suggested that GXK could reduce the inflammation in ox-LDL-induced RAW264.7 cells.

### GXK Enhances Efferocytosis in RAW264.7 Cells

**FIGURE 6:**
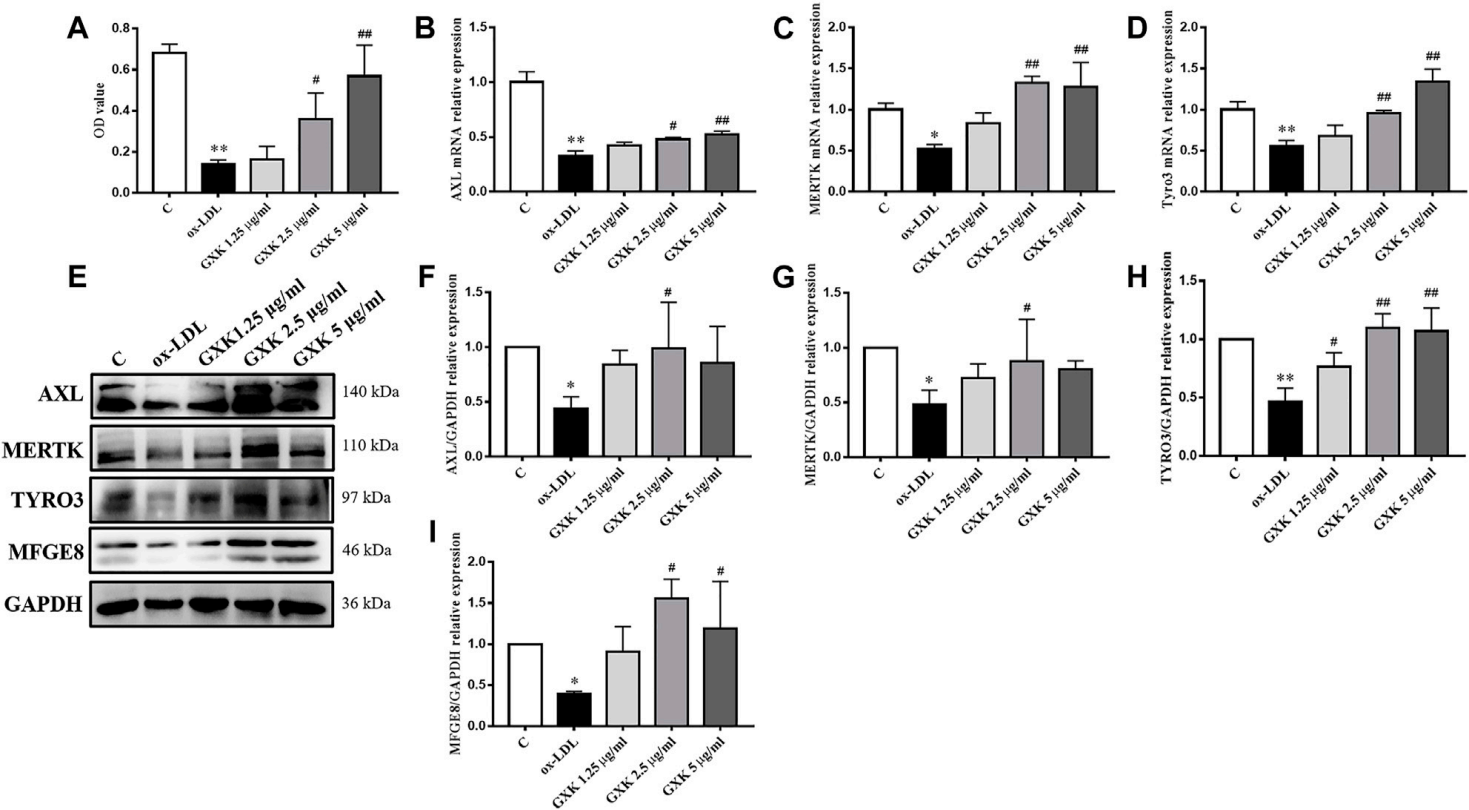
Effects of GXK on efferocytosis related molecules in RAW264.7 cells. **(A)** Neutral red uptake assay was used to detect phagocytic ability of macrophages, n = 3. **(B–D)** Gene expression of IL-1β, IL-6 and TNF-α was measured by RT-qPCR method, n = 3. **(E)** Protein was extracted from RAW264.7 cells. Then AXL, MERTK, TYRO3 and MFGE8 were tested by western blotting assay, n = 3. **(F–I)** The quantitative results were depicted. C means control group. MFGE8, milk fat globule-epidermal growth factor. Data are expressed as mean ± SD. *p < 0.05, **p < 0.01 vs. C group; #*p* < 0.05, ##*p* < 0.01 vs. ox-LDL-stimulated group.

The effects of GXK on the phagocytic ability of cells was determined using Neutral red uptake assay. As shown in Figure 6A, the phagocytic ability in ox-LDL-stimulated group was significantly decreased (*p* < 0.01). GXK weakened the effect of ox-LDL and increased the phagocytic ability (*p* < 0.05). The efferocytosis-related molecules in RAW264.7 cells were also investigated. As shown in Figures 6B–D, the mRNA levels of AXL, MERTK and TYRO3 in ox-LDL-stimulated group decreased compared with those in the control group (*p* < 0.05). GXK intervention could increase the mRNA levels of those genes (*p* < 0.05). These results were consistent with the changes in protein levels in RAW264.7 cells, as revealed by Western Blot (*p* < 0.05, Figures 6E–H). In addition, GXK also increased MFGE8 protein expression (*p* < 0.05, Figure 6I). These results suggested that GXK could improve the efferocytosis in ox-LDL-induced RAW264.7 cells.

### GXK Inhibites MAPKs-Related Protein Expression Levels in RAW264.7 Cells

**FIGURE 7:**
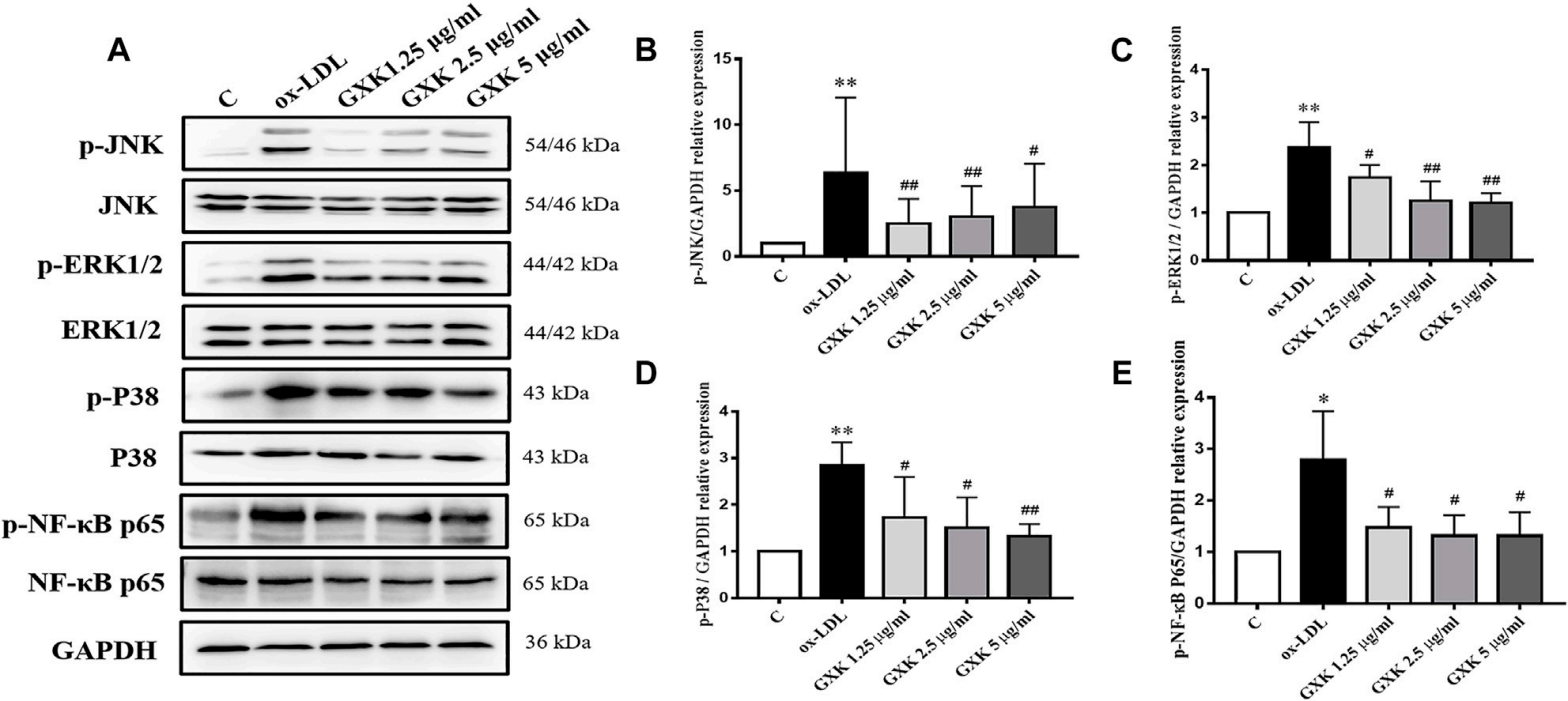
Further investigation of effects of GXK on MAPKs signaling pathway in RAW264.7 cells. **(A)** Protein was extracted from RAW264.7 cells. Then the total and phosphorylated levels of JNK, ERK1/2, p38 and NF-κB P65 were determined by western blotting assay, n = 3. **(B–E)** The quantitative results were depicted. C means control group Data are expressed as mean ± SD. *p < 0.05, **p < 0.01 vs. C group; #*p* < 0.05, ##*p* < 0.01 vs. ox-LDL-stimulated group.

To testify the effect of GXK on MAPKs/NF-κB signaling pathway, phosphorylation of p38, JNK, ERK1/2 and NF-κB p65 was examined by Western Blot assays (Figure 7A). As Figures 7B–E showed, the expression level of MAPKs/NF-κB in ox-LDL-stimulated group was higher than that in the control group (*p* < 0.05). But a significant reduction in the phosphorylation levels of these kinases was observed in the GXK group (*p* < 0.05). These results indicated that GXK could inhibit the activation of MAPKs/NF-κB pathways induced by ox-LDL.

### GXK Decreases Inflammatory Cytokine Levels and Enhances Efferocytosis Through the MAPKs Signaling Pathway

**FIGURE 8:**
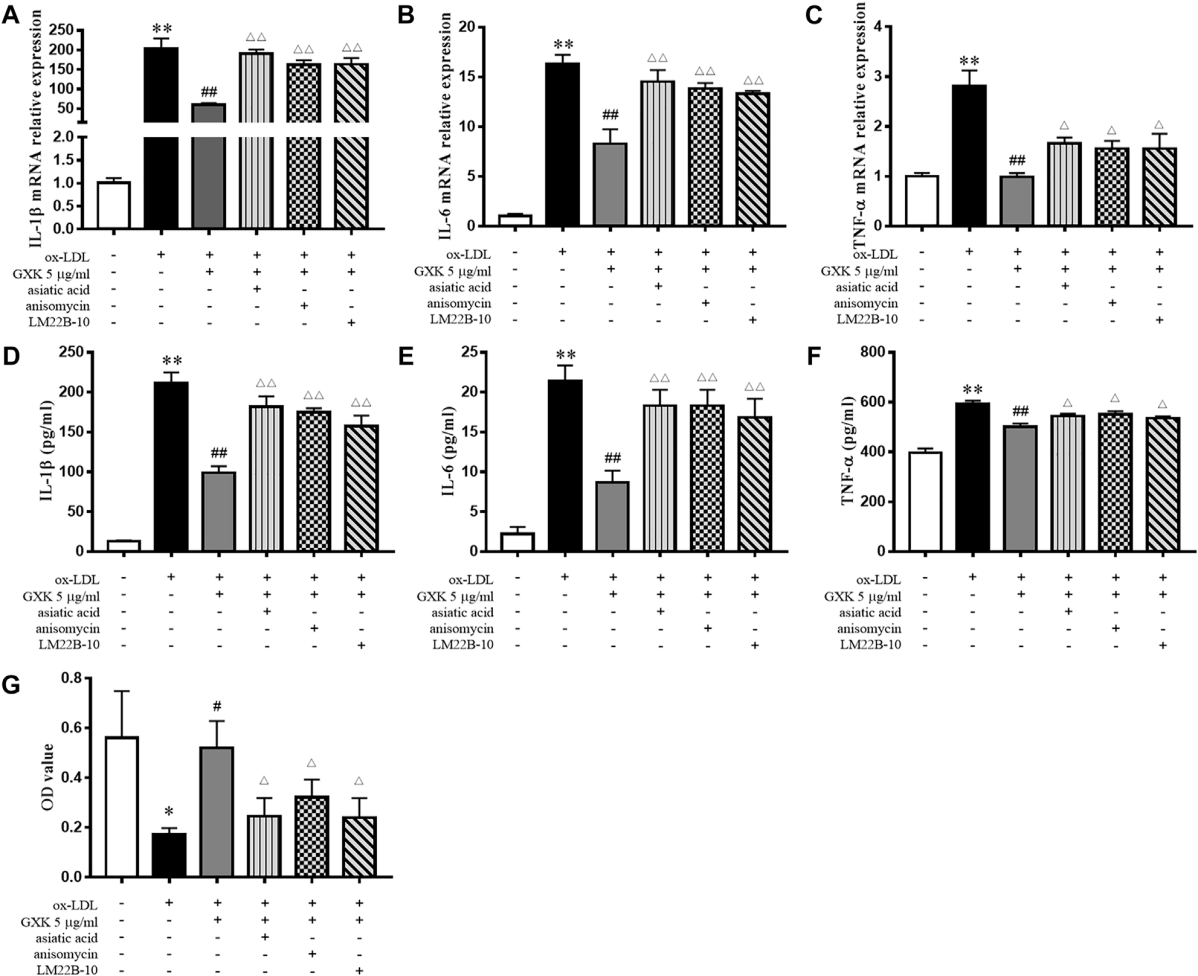
GXK functions mainly through the MAPKs signaling pathway. Cells were pretreated with 10 μM asiatic acid, 10 μM anisomycin, or 10 μM LM22B-10 for 0.5 h, then treated with oxLDL for 24 h. **(A–C)** Then gene expression of IL-1β, IL-6 and TNF-α was measured by RT-qPCR method and **(D–F)** production of these three cytokines were explored by ELISA kits, n = 3. **(G)** The phagocytic ability of macrophages was detected by neutral red uptake assay, n = 3. Data are expressed as mean ± SD. *p < 0.05, **p < 0.01 vs. group without ox-LDL or GXK; #*p* < 0.05, ##*p* < 0.01 vs. group with ox-LDL; △*p* < 0.05, △△*p* < 0.01 vs. group with ox-LDL and GXK.

To verify the role of MAPKs in the function of GXK, three specific activators (asiatic acid, a p38 activator; anisomycin, a JNK activator; LM22B-10, an ERK1/2 activator) were employed. As shown in Figures 8A–F, the expression levels of IL-1β, IL-6 and TNF-α were increased after treatment of three activators, compared to those in GXK 5 μg/ml-treated group (*p* < 0.05). Additionally, these activators significantly promoted the production of IL-1β, IL-6, and TNF-α, as shown in ELISA analysis (*p* < 0.05). In addition, these activators reversed the effect of GXK to promote the phagocytic ability of the cell, as shown in Neutral red uptake assay (*p* < 0.05) (Figure 8G). These results suggested that GXK could regulate inflammation and efferocytosis in ox-LDL-induced RAW264.7 cells through MAPKs pathway.

## Discussion

Lipid metabolism is dysregulated during the development of AS (Poznyak et al., 2020). Accumulated LDL can be oxidized into ox-LDL which binds to Toll-like receptors (TLRs) to induce foam cell formation and inflammation (Miller et al., 2011). Therefore, lipid-lowering therapy remains an effective strategy against AS. The results of *in vivo* experiments showed that GXK intervention significantly decreased serum lipid (TC, TG and LDL-C) levels and plaque accumulation in the aortic roots (Figures 1A–E). These results indicate that GXK counters AS by restoring lipid metabolism.

A large amount of pro-inflammatory cytokines is released by macrophages after lipid uptake, which may result in strong inflammation (Tousoulis et al., 2016). Accumulated evidence has demonstrated that ox-LDL produces pathophysiological effects, including the release of proinflammatory cytokines, overexpression of cell adhesion molecules (VCAM-1, ICAM-1) and MCP-1, all enhancing inflammatory processes (Di et al., 2017). High-level ox-LDL also leads to persist activation of the scavenger receptor LOX-1, subsequently activating NF-κB. This process in turn increases the level of iNOS, thus contributing to the development of AS (Gliozzi et al., 2019). Current researches support that the inflammatory response has an important role in the progression of AS. Thus, anti-inflammatory strategies should be designed to prevent the progression of atherosclerotic disease (Gaudino and Crea, 2019). The main finding of this study is that GXK could inhibit atherosclerotic changes by triggering anti-inflammatory response. The protein levels of iNOS, VCAM-1, LOX-1 and MCP-1 significantly decreased after GXK treatment. GXK decreased mRNA and protein levels of IL-1β, IL-6 and TNF-α in the mice and cells (Figures 2, 5), indicating that GXK inhibits inflammation in HFD-induced LDLR^−/−^ mice and ox-LDL-induced RAW264.7 cells.

Recent studies have shown that MAPKs signaling pathway is activated to attenuate inflammation in AS (Wang et al., 2020a; Wang et al., 2020b; Lu et al., 2020), making MAPKs an ideal target of drug therapy. NF-κB, a critical transcription factor in inflammatory responses, can be activated by ox-LDL to promote AS (Chávez-Sánchez et al., 2010). The main active compounds of GXK include astragaloside IV, stachydrine hydrochloride, tanshinone IIA, hesperetin, butane diacid, and 3,29-Dibenzoyl karounitriol. There have been many researches about the effect of these active compounds on the MAPK signaling pathway. Aastragaloside IV has an anti-inflammatory via regulated MAPK signaling pathway in human bronchial epithelial cells (Hsieh et al., 2020). Sequencing analysis and bioinformatics tools found tanshinone IIA down-regulated genes mainly participate in the MAPK signaling pathway in atherosclerotic mice (Chen et al., 2020). And hesperetin inhibits microglia-mediated neuroinflammation by down-regulating the phosphorylation of MAPK signaling pathway (Jo et al., 2019). To explore the anti-inflammatory mechanism of GXK, we detected the total and phosphorylated JNK, ERK1/2, p38 and NF-κB p65 expression in the mice and cells. As shown by the results, GXK decreased phospho-MAPKs/NF-κB in the mice and cells (Figures 4, 7). And MAPKs activators reversed the effects of GXK in repressing inflammation (Figures 8A–F). These suggest that GXK achieves its anti-inflammatory function via MAPKs signaling pathway.

Efferocytosis is a physiological process in which phagocytic cells (such as macrophages) clear ACs to maintain body homeostasis (Boada-Romero et al., 2020). The efferocytosis consists of four stages: recognition (“find me”), capture (“eat me”), endocytosis, digestion and degradation of ACs by phagocytes (Boada-Romero et al., 2020). This process involves many signaling molecules. The “find me” molecules include signaling molecules, such as sphingosine-1-phosphate (S1P) (Gude et al., 2008) and CX3C chemokine ligand 1 (CX3CL1) protein, CX3C chemokine receptor 1 (CX3CR1) (Truman et al., 2008) and TAM receptors (Tyro3, Axl, and Mer) (Geng et al., 2017); and bridging molecules, such as milk fat globule-epidermal growth factor (MFGE8) (Trahtemberg and Mevorach, 2017). Effective efferocytosis prevents secondary necrosis and terminate inflammatory responses in AS, increasing plaque stability (Yurdagul et al., 2017). Increased vulnerability of plaque can easily lead to plaque rupture, resulting in serious cardiovascular events (Seneviratne et al., 2017). In this study, GXK significantly promoted the expression of α-SMA, a benign component in the plaque, and reduced necrotic core area, indicating that GXK significantly reduces the plaque vulnerability (Figures 1F–K).

In AS, efferocytosis-related molecules may function to resolve inflammation. However, the effect of efferocytosis is gradually impaired with the formation of atherosclerotic plaques. Schrijvers et al. have found that the rate of ACs clearance is higher in human tonsils than in atherosclerotic plaques (Schrijvers et al., 2005). Thus, stoking up efferocytosis could be a new treatment option for AS. TAM receptors (Tyro3, Axl, and Mer) belong to a family of receptor tyrosine kinases that can suppress inflammation (Rothlin et al., 2015). Axl attenuates inflammation via inhibiting the activation of Toll-like receptors (TLRs)/nuclear factor-kappa B (NF-κB) pathway and nucleotide-binding domain leucine-rich repeat and pyrin domain containing receptor 3 (NLRP3) (Han et al., 2016; Wu et al., 2018). Previous studies have found that inflammation enhances and AS aggravates in mice lacking MERTK and MFGE8 (Ait-Oufella et al., 2007; Cai et al., 2017; Triantafyllou et al., 2018). Several studies have shown that MFGE8 is a novel anti-inflammatory factor in multisystem diseases, such as neonatal sepsis, hepatic steatosis, rheumatoid arthritis and kidney injury (Albus et al., 2016; Hansen et al., 2017; Zhao et al., 2019; Zhang et al., 2021). Additionally, MFGE8 level decreases in the CHD group and is negatively associated with the severity of coronary artery stenosis and the risk of clinical events (Dai et al., 2014). Our results are in accordance with these reports: the mRNA and protein levels of AXL, MERTK, TYRO3 and MFGE8 expression in mouse aortas and RAW264.7 cells decreased in the model group; by contrast, GXK up-regulated the expression of these molecules (Figures 3, 6) and increased the phagocytic ability of ox-LDL-induced RAW264.7 cells. The results indicate that GXK improves AS through enhancing efferocytosis.

Moreover, MAPKs is significantly repressed in the absence of TAM (Zheng et al., 2015). And the phosphorylation of MAPKs signaling proteins is also reversed by MFGE8 (Xu et al., 2018). Previous researches have shown that p38 MAPK phosphorylation promotes MERTK shedding, thus leading to defective efferocytosis and atherosclerotic progression (Zhang et al., 2019). A clinical research has demonstrated that blocking p38 MAPK elevation restores efferocytosis and resolves inflammation in the elderly (De Maeyer et al., 2020). In summary, the efferocytosis interacts with the MAPKs signaling pathway to resolve inflammation. Our results showed that the MAPKs activators reversed the ability of GXK to promote phagocytosis, suggesting that GXK promotes efferocytosis via MAPK signaling pathway (Figure 8G).

In conclusion, this article explored the primary anti-inflammatory mechanism of GXK in AS. GXK can promote efferocytosis and suppress MAPKs signaling pathway, thus decreasing the production of pro-inflammatory cytokines in LDLR^−/−^ mice and ox-LDL-stimulated RAW264.7 cells. GXK exerts its anti-inflammatory effect to counter AS, the underlying mechanism of which needs further studies.

## Data Availability

The original contributions presented in the study are included in the article/Supplementary Material, further inquiries can be directed to the corresponding author.
