# Supplementary figures and images for: Guanxinkang Decoction Attenuates the Inflammation in Atherosclerosis by Regulating Efferocytosis and MAPKs Signaling Pathway in LDLR−/− Mice and RAW264.7 Cells (part 1 of 3)
Source: Front Pharmacol. 2021 Dec 7;12:731769. doi: 10.3389/fphar.2021.731769 (PMC8688952; doi:10.3389/fphar.2021.731769)

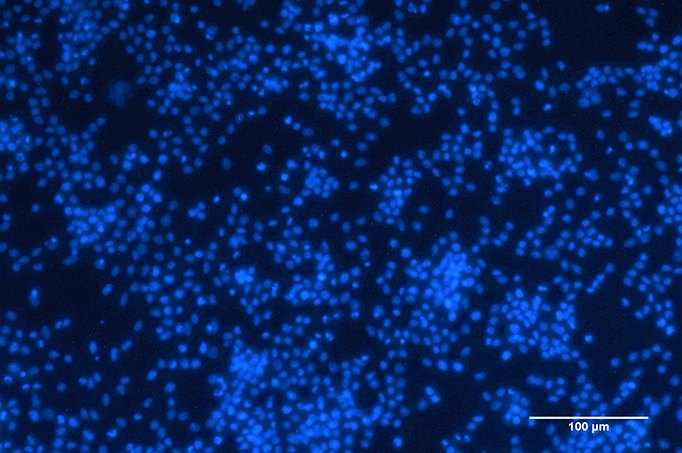

Supplement: Supplementary file 1 [file DataSheet3.ZIP › Immunofluorescence Staining/C1-DAPI.tif]

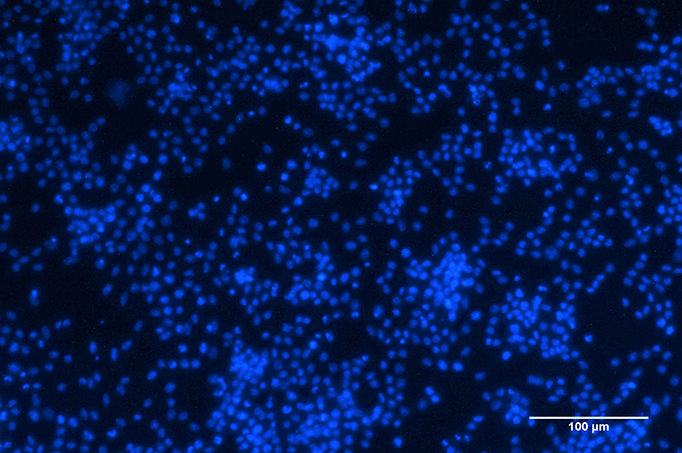

Supplement: Supplementary file 1 [file DataSheet3.ZIP › Immunofluorescence Staining/C1-MERGE.tif]

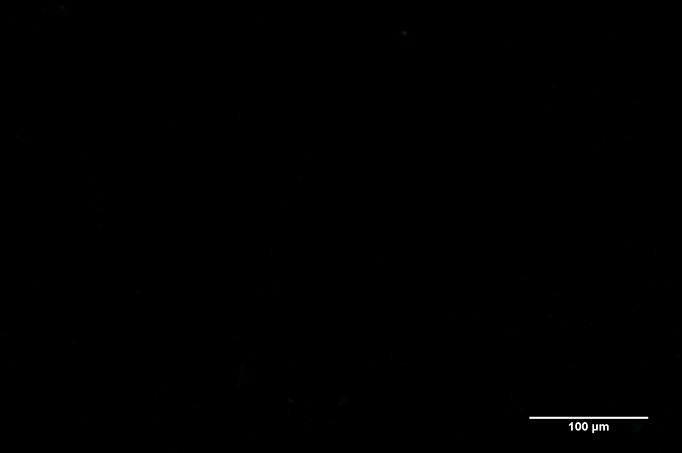

Supplement: Supplementary file 1 [file DataSheet3.ZIP › Immunofluorescence Staining/C1-TNF.tif]

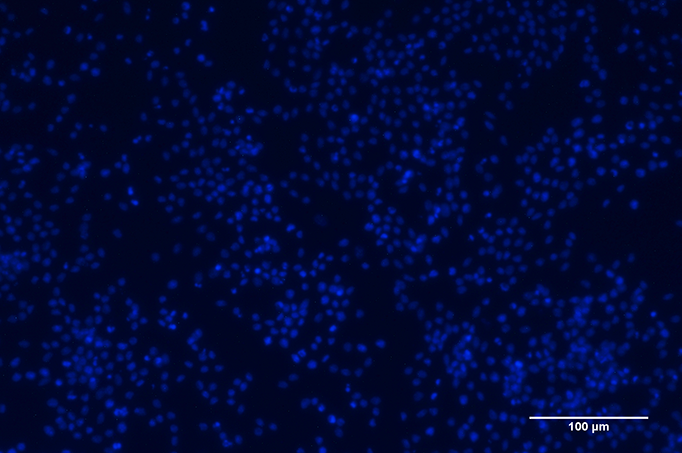

Supplement: Supplementary file 1 [file DataSheet3.ZIP › Immunofluorescence Staining/C2-DAPI.tif]

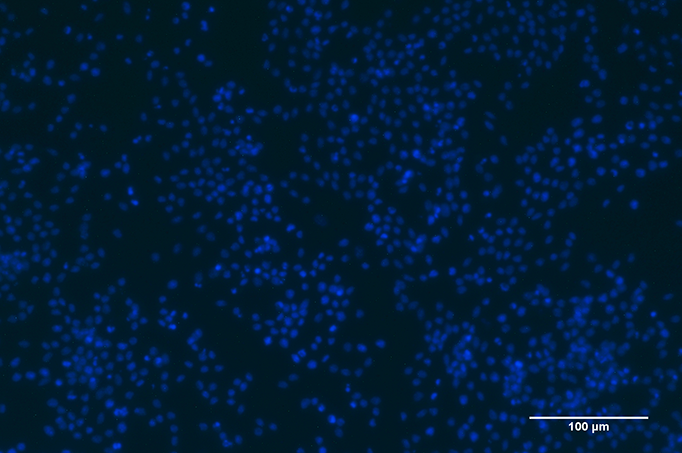

Supplement: Supplementary file 1 [file DataSheet3.ZIP › Immunofluorescence Staining/C2-MERGE.tif]

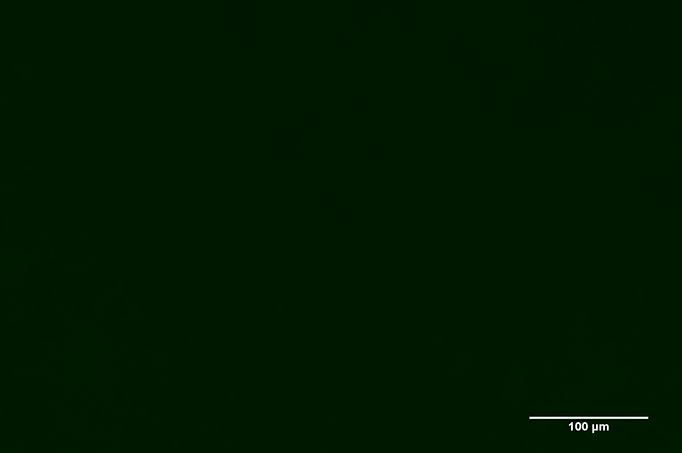

Supplement: Supplementary file 1 [file DataSheet3.ZIP › Immunofluorescence Staining/C2-TNF.tif]

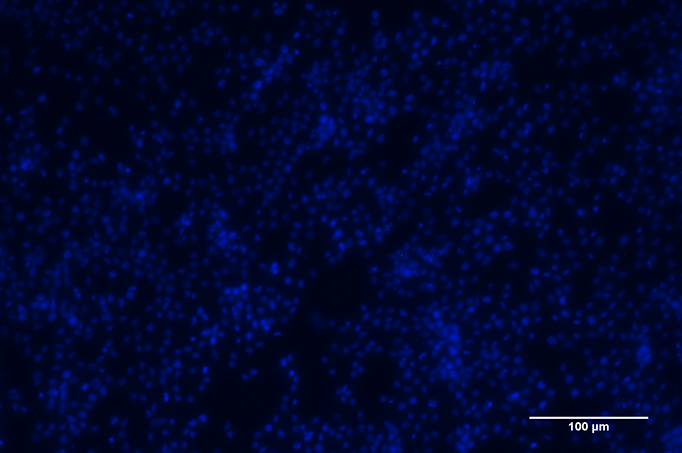

Supplement: Supplementary file 1 [file DataSheet3.ZIP › Immunofluorescence Staining/C3-DAPI.tif]

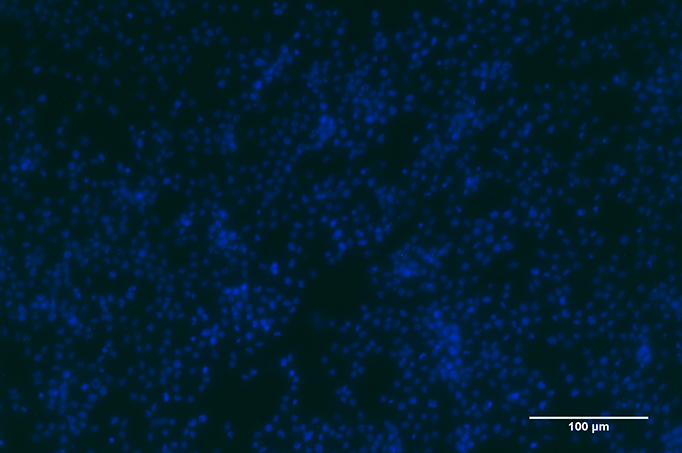

Supplement: Supplementary file 1 [file DataSheet3.ZIP › Immunofluorescence Staining/C3-MERGE.tif]

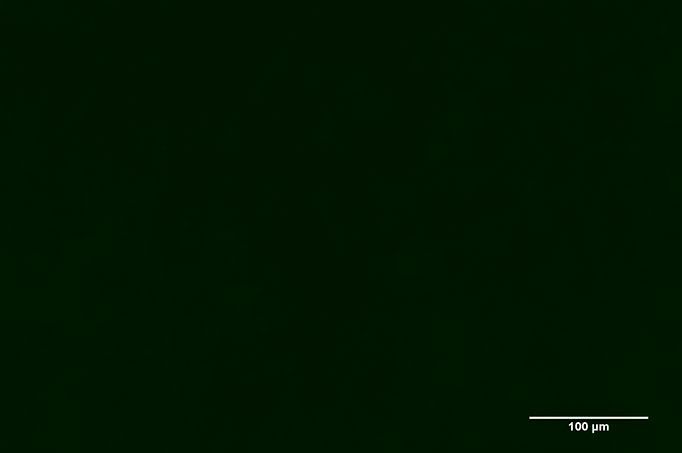

Supplement: Supplementary file 1 [file DataSheet3.ZIP › Immunofluorescence Staining/C3-TNF.tif]

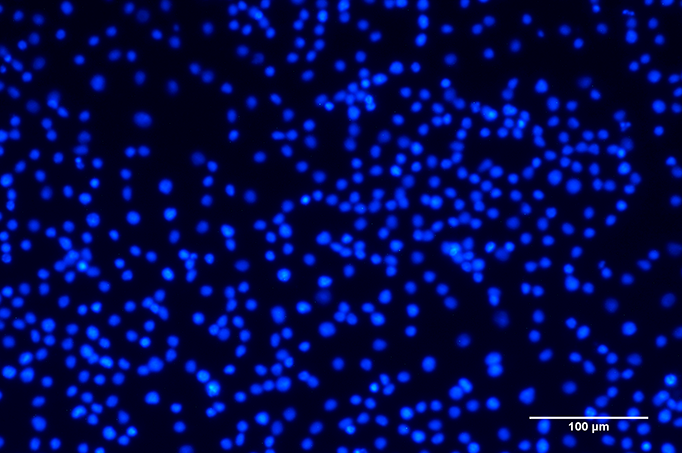

Supplement: Supplementary file 1 [file DataSheet3.ZIP › Immunofluorescence Staining/FH1-DAPI.tif]

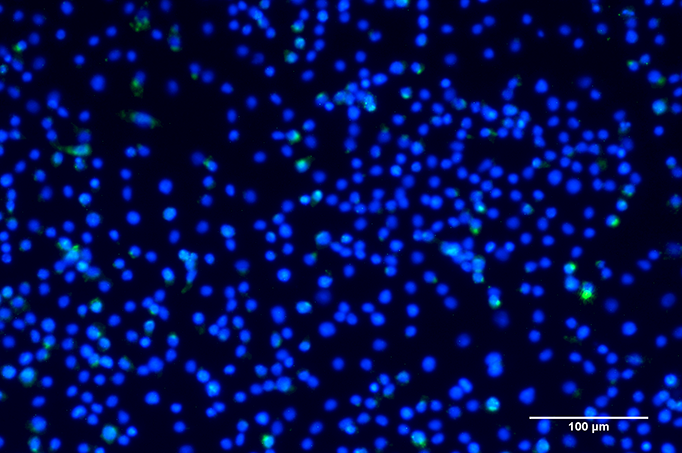

Supplement: Supplementary file 1 [file DataSheet3.ZIP › Immunofluorescence Staining/FH1-MERGE.tif]

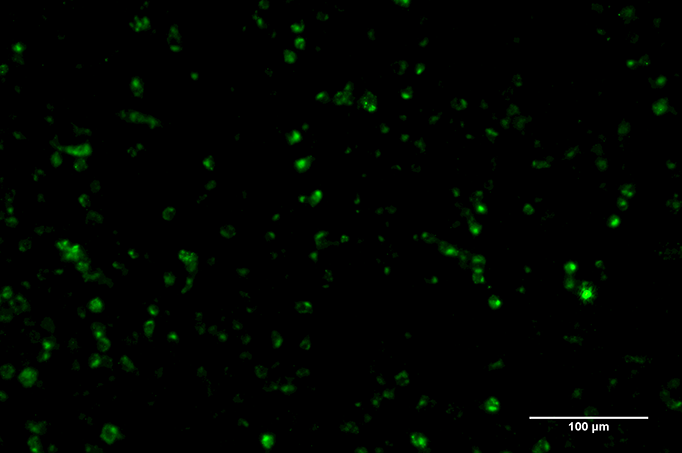

Supplement: Supplementary file 1 [file DataSheet3.ZIP › Immunofluorescence Staining/FH1-TNF.tif]

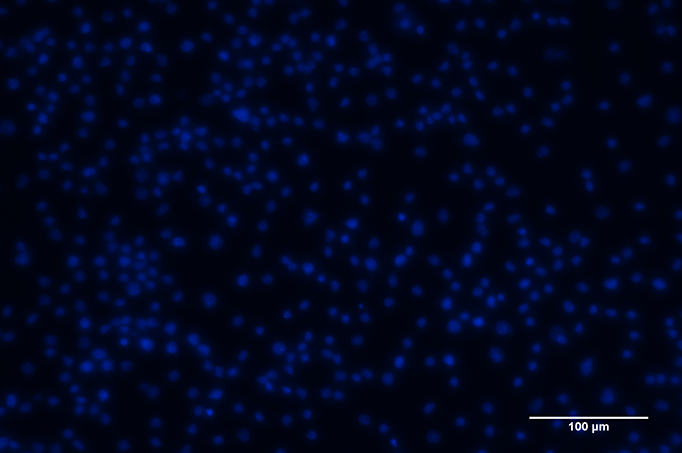

Supplement: Supplementary file 1 [file DataSheet3.ZIP › Immunofluorescence Staining/FH2-DAPI.tif]

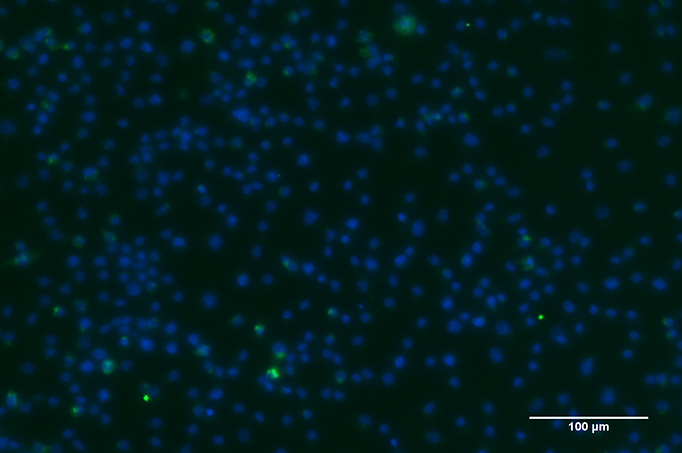

Supplement: Supplementary file 1 [file DataSheet3.ZIP › Immunofluorescence Staining/FH2-MERGE.tif]

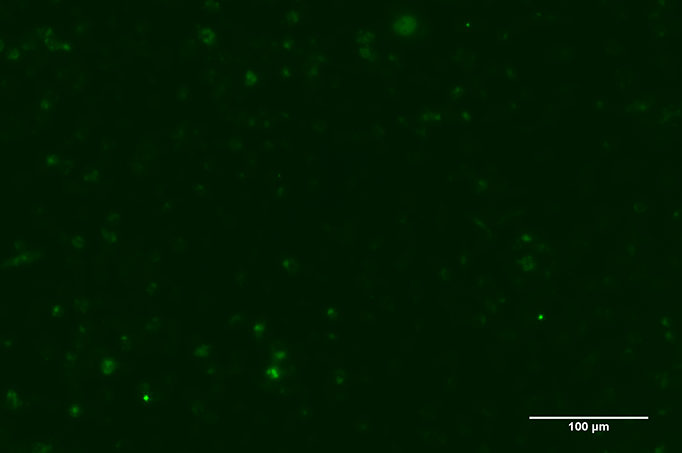

Supplement: Supplementary file 1 [file DataSheet3.ZIP › Immunofluorescence Staining/FH2-TNF.tif]

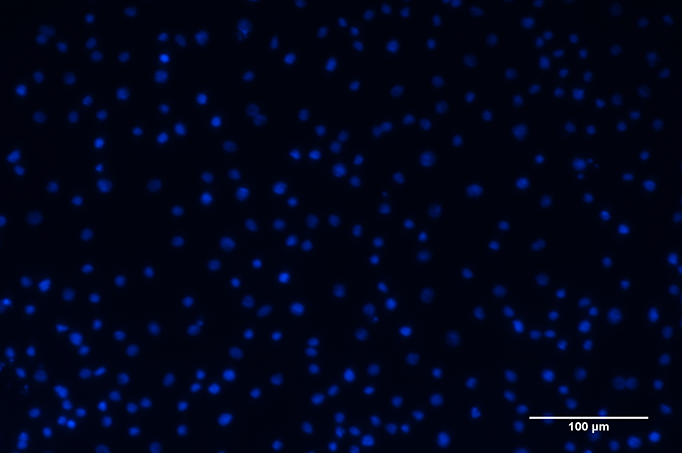

Supplement: Supplementary file 1 [file DataSheet3.ZIP › Immunofluorescence Staining/FH3-DAPI.tif]

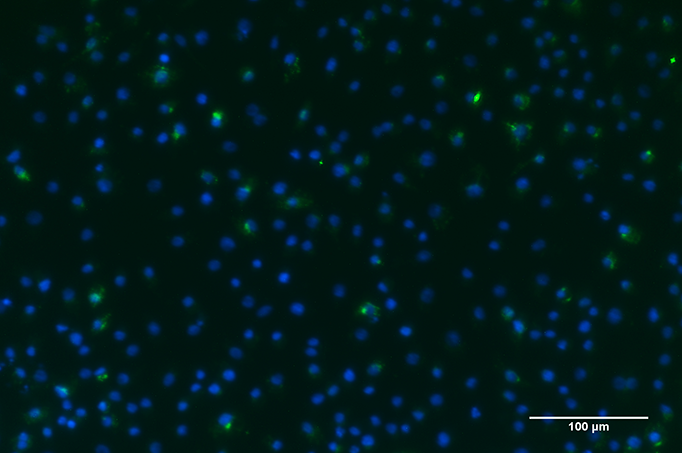

Supplement: Supplementary file 1 [file DataSheet3.ZIP › Immunofluorescence Staining/FH3-MERGE.tif]

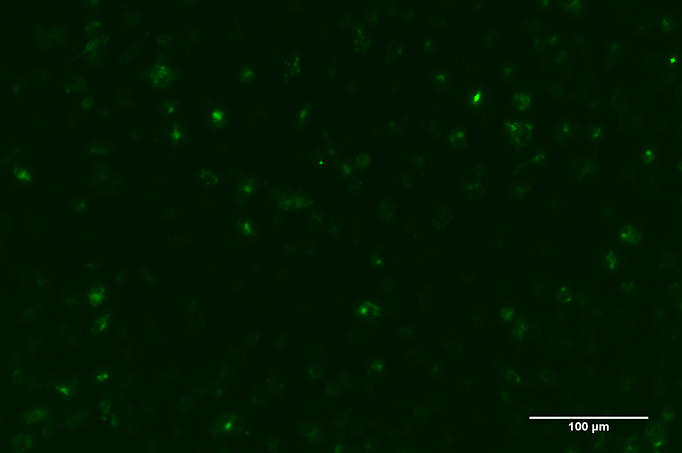

Supplement: Supplementary file 1 [file DataSheet3.ZIP › Immunofluorescence Staining/FH3-TNF.tif]

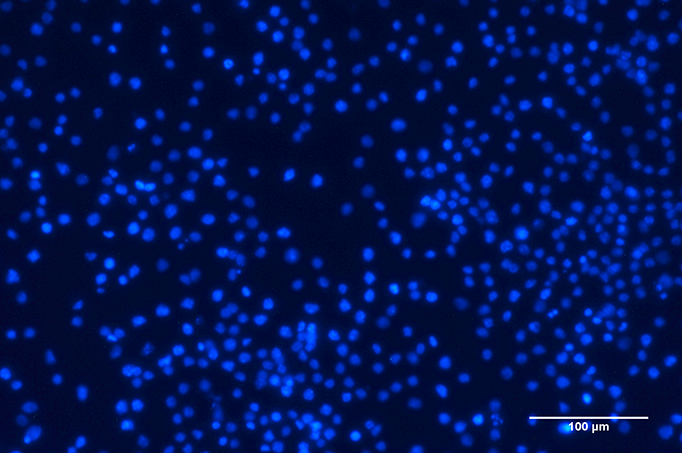

Supplement: Supplementary file 1 [file DataSheet3.ZIP › Immunofluorescence Staining/FL1-DAPI.tif]

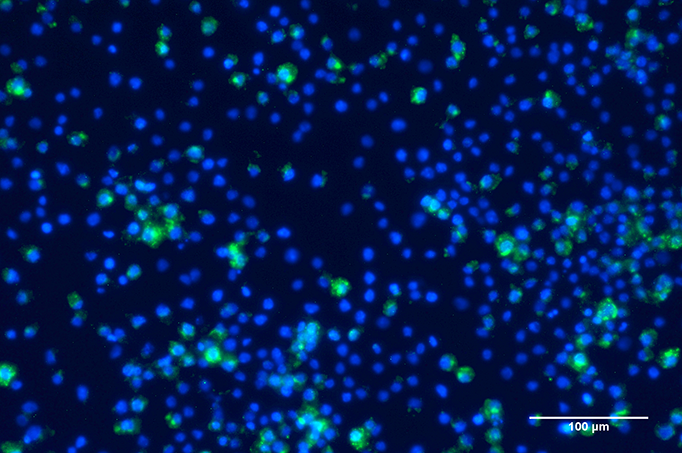

Supplement: Supplementary file 1 [file DataSheet3.ZIP › Immunofluorescence Staining/FL1-MERGE.tif]

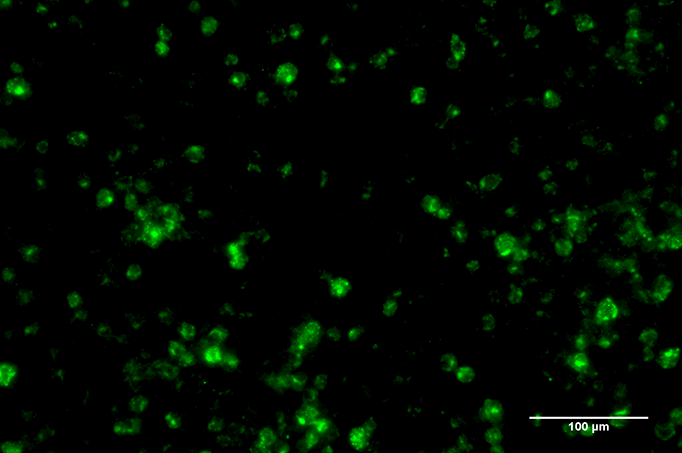

Supplement: Supplementary file 1 [file DataSheet3.ZIP › Immunofluorescence Staining/FL1-TNF.tif]

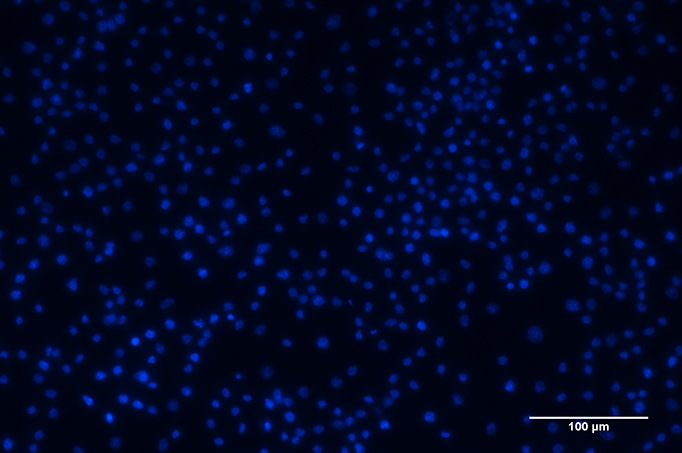

Supplement: Supplementary file 1 [file DataSheet3.ZIP › Immunofluorescence Staining/FL2-DAPI.tif]

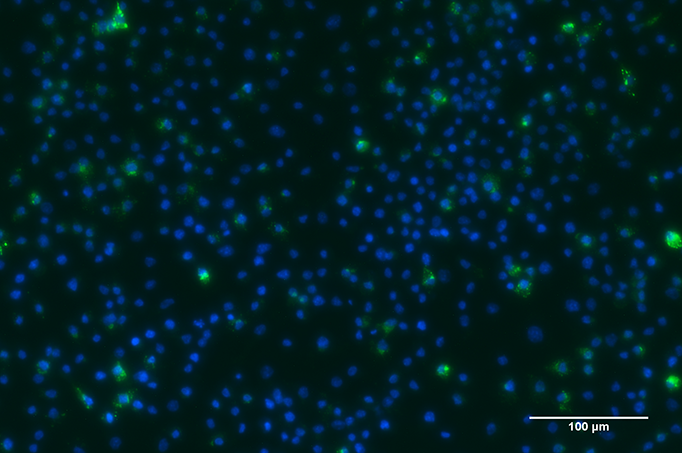

Supplement: Supplementary file 1 [file DataSheet3.ZIP › Immunofluorescence Staining/FL2-MERGE.tif]

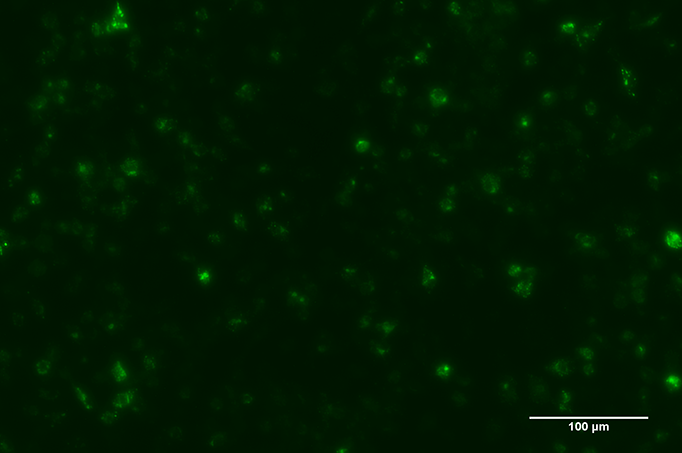

Supplement: Supplementary file 1 [file DataSheet3.ZIP › Immunofluorescence Staining/FL2-TNF.tif]

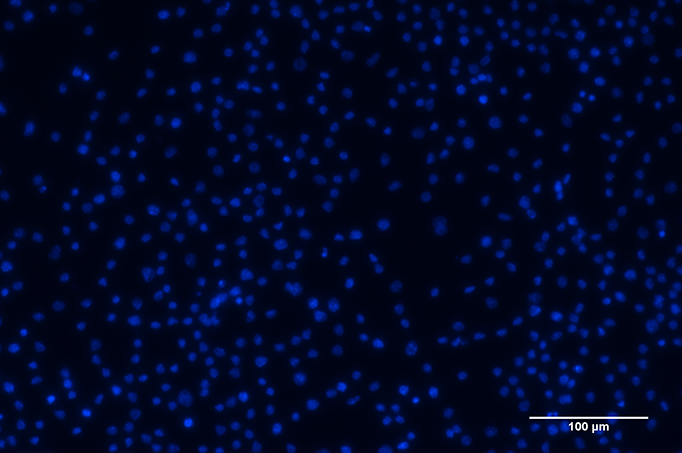

Supplement: Supplementary file 1 [file DataSheet3.ZIP › Immunofluorescence Staining/FL3-DAPI.tif]

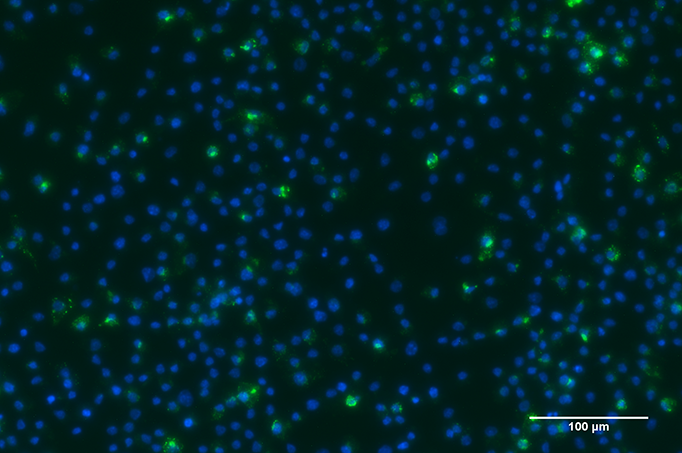

Supplement: Supplementary file 1 [file DataSheet3.ZIP › Immunofluorescence Staining/FL3-MERGE.tif]

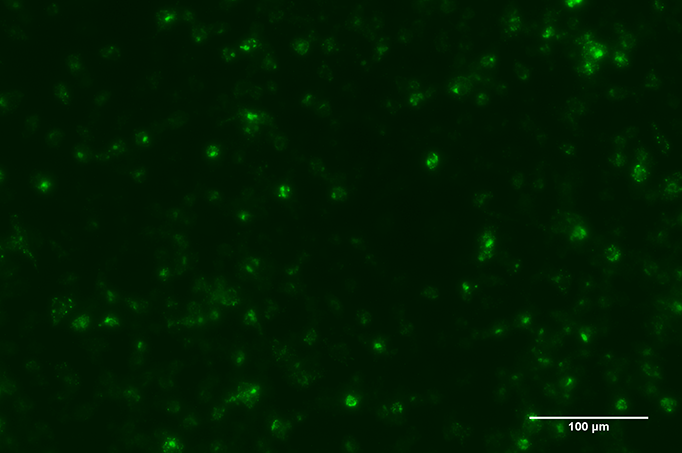

Supplement: Supplementary file 1 [file DataSheet3.ZIP › Immunofluorescence Staining/FL3-TNF.tif]

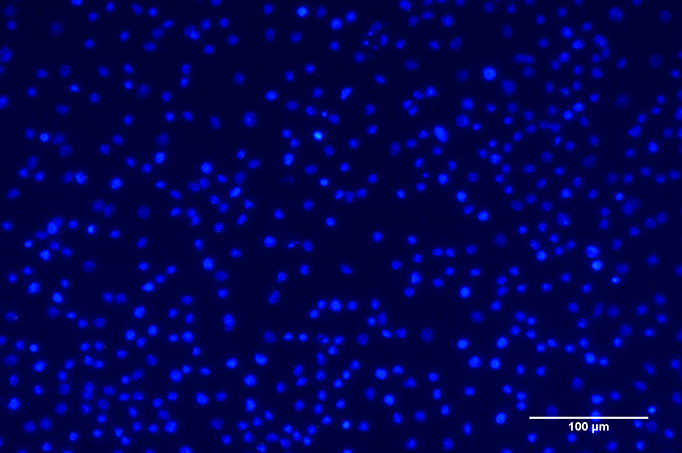

Supplement: Supplementary file 1 [file DataSheet3.ZIP › Immunofluorescence Staining/FM1-DAPI.tif]

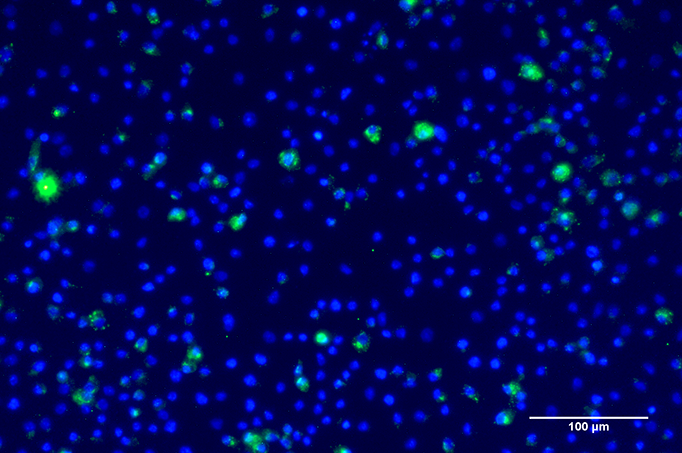

Supplement: Supplementary file 1 [file DataSheet3.ZIP › Immunofluorescence Staining/FM1-MERGE.tif]

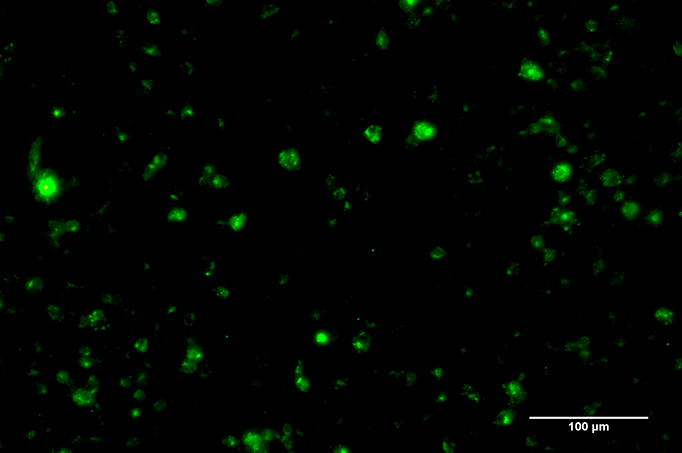

Supplement: Supplementary file 1 [file DataSheet3.ZIP › Immunofluorescence Staining/FM1-TNF.tif]

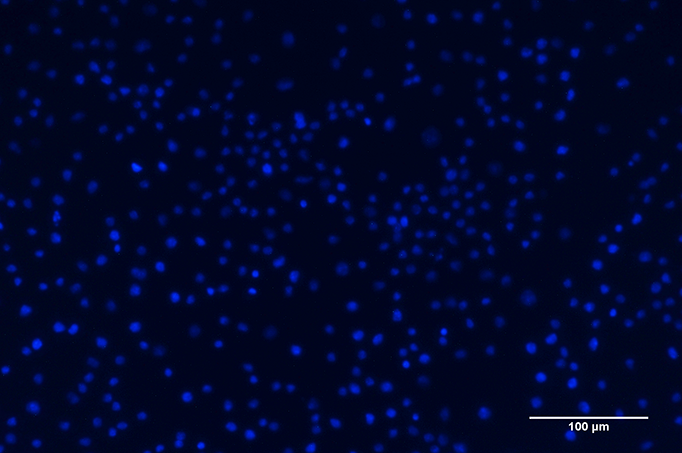

Supplement: Supplementary file 1 [file DataSheet3.ZIP › Immunofluorescence Staining/FM2-DAPI.tif]

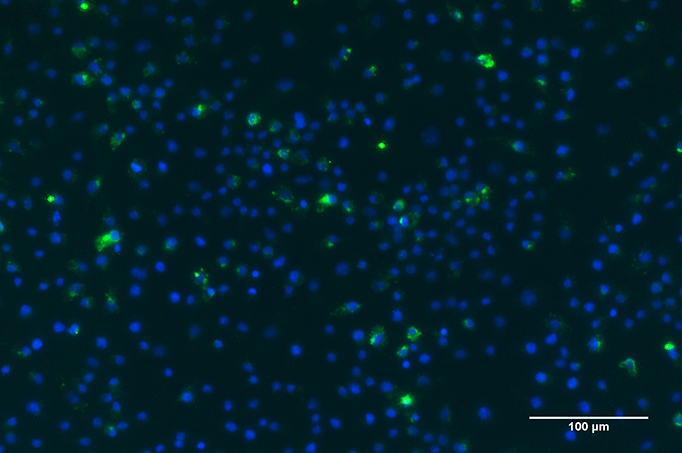

Supplement: Supplementary file 1 [file DataSheet3.ZIP › Immunofluorescence Staining/FM2-MERGE.tif]

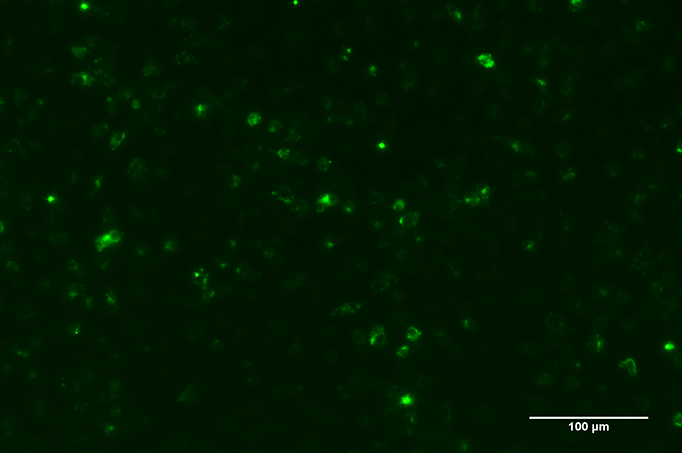

Supplement: Supplementary file 1 [file DataSheet3.ZIP › Immunofluorescence Staining/FM2-TNF.tif]

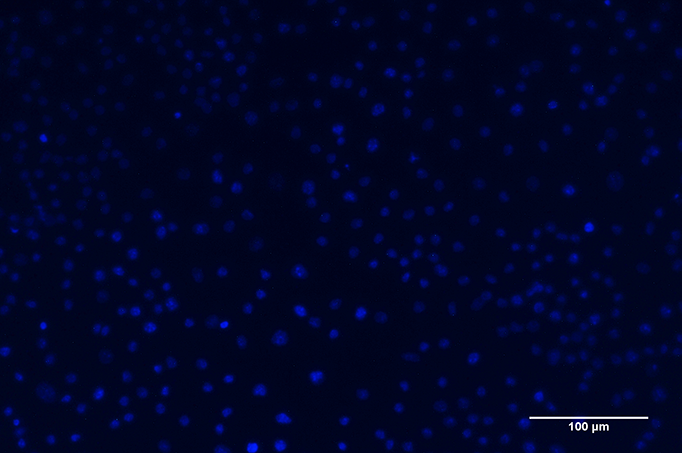

Supplement: Supplementary file 1 [file DataSheet3.ZIP › Immunofluorescence Staining/FM3-DAPI.tif]

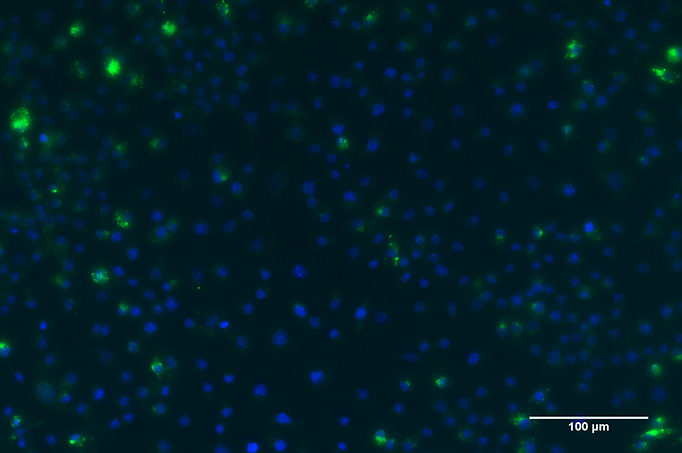

Supplement: Supplementary file 1 [file DataSheet3.ZIP › Immunofluorescence Staining/FM3-MERGE.tif]

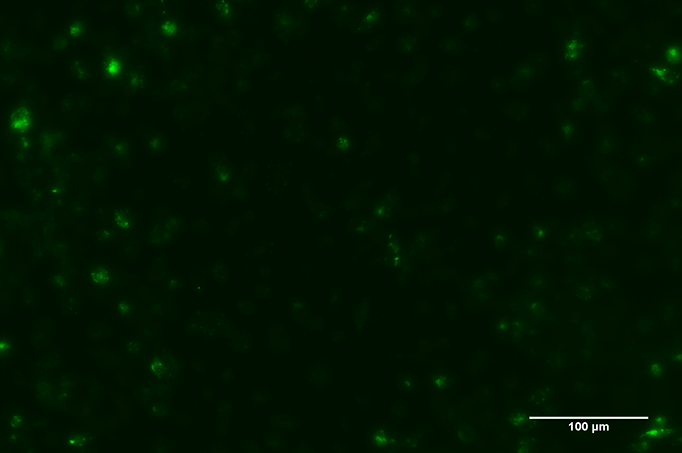

Supplement: Supplementary file 1 [file DataSheet3.ZIP › Immunofluorescence Staining/FM3-TNF.tif]

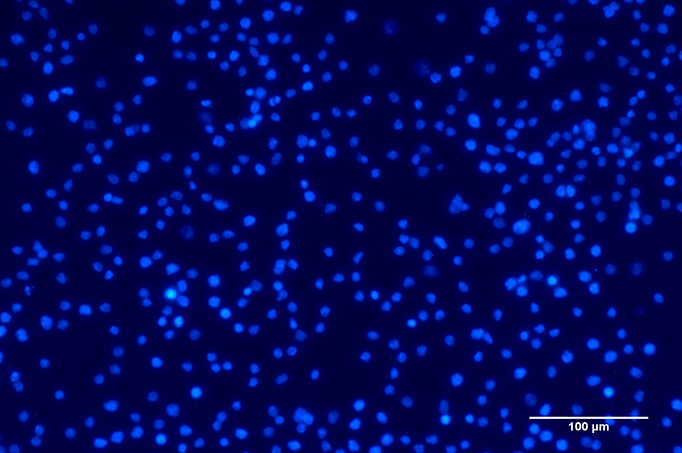

Supplement: Supplementary file 1 [file DataSheet3.ZIP › Immunofluorescence Staining/M1-DAPI.tif]

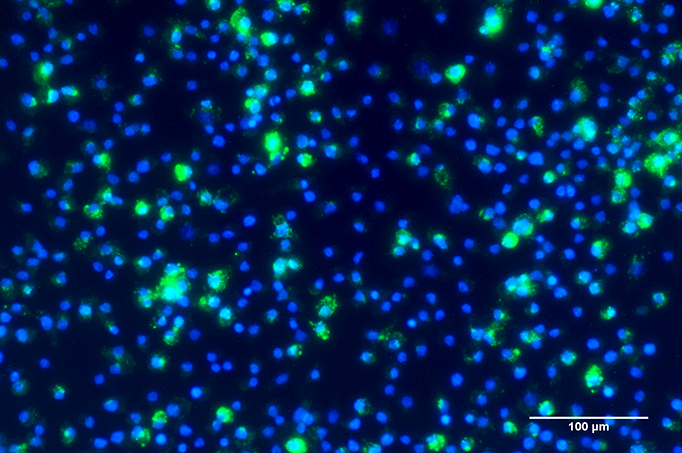

Supplement: Supplementary file 1 [file DataSheet3.ZIP › Immunofluorescence Staining/M1-MERGE.tif]

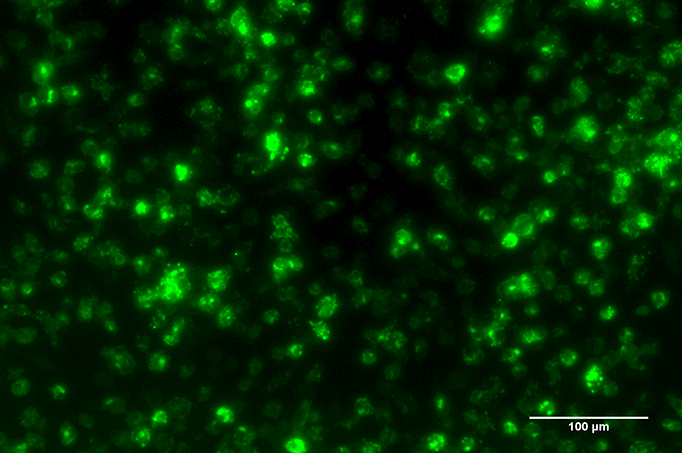

Supplement: Supplementary file 1 [file DataSheet3.ZIP › Immunofluorescence Staining/M1-TNF.tif]

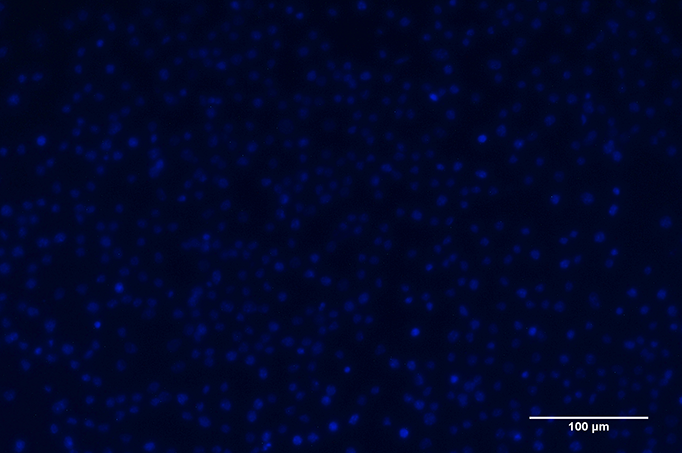

Supplement: Supplementary file 1 [file DataSheet3.ZIP › Immunofluorescence Staining/M2-DAPI.tif]

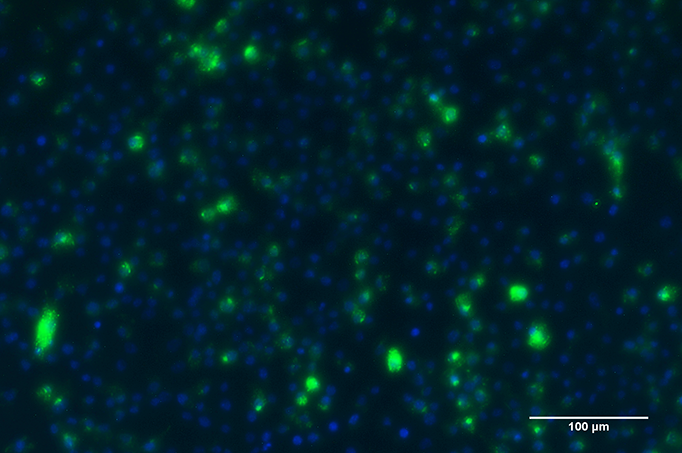

Supplement: Supplementary file 1 [file DataSheet3.ZIP › Immunofluorescence Staining/M2-MERGE.tif]

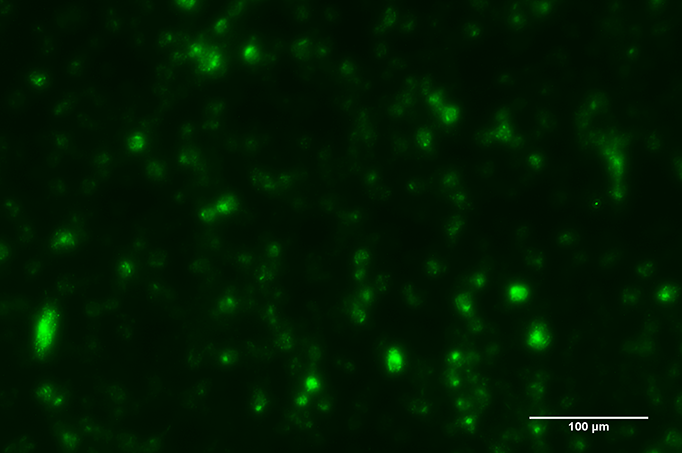

Supplement: Supplementary file 1 [file DataSheet3.ZIP › Immunofluorescence Staining/M2-TNF.tif]

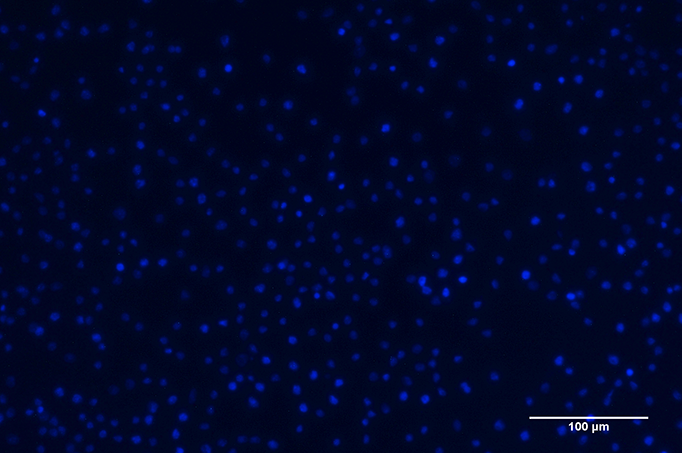

Supplement: Supplementary file 1 [file DataSheet3.ZIP › Immunofluorescence Staining/M3-DAPI.tif]

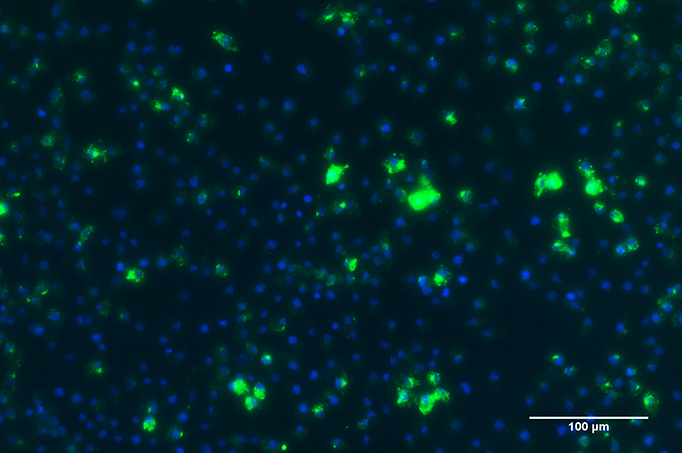

Supplement: Supplementary file 1 [file DataSheet3.ZIP › Immunofluorescence Staining/M3-MERGE.tif]

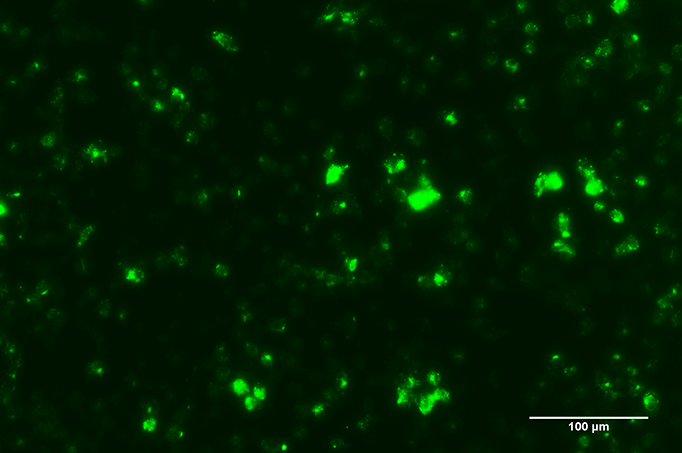

Supplement: Supplementary file 1 [file DataSheet3.ZIP › Immunofluorescence Staining/M3-TNF.tif]

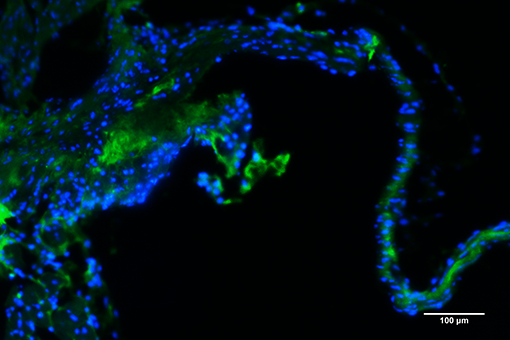

Supplement: Supplementary file 3 [file DataSheet4.ZIP › figure 1-F(p65)/ATO-1.tif]

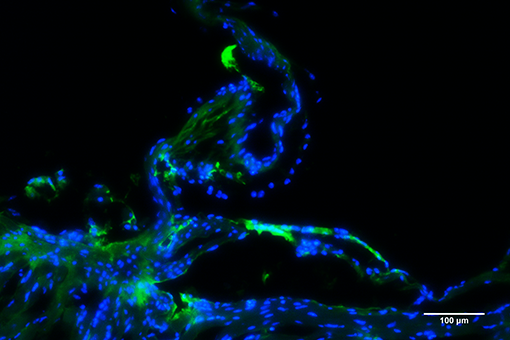

Supplement: Supplementary file 3 [file DataSheet4.ZIP › figure 1-F(p65)/ATO-2.tif]

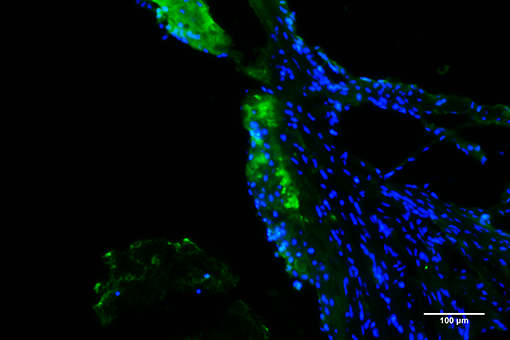

Supplement: Supplementary file 3 [file DataSheet4.ZIP › figure 1-F(p65)/ATO-3tif.tif]

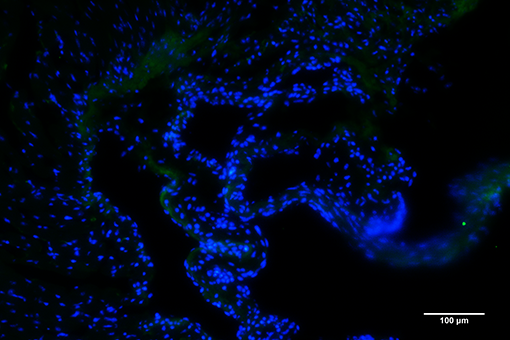

Supplement: Supplementary file 3 [file DataSheet4.ZIP › figure 1-F(p65)/C-1.tif]

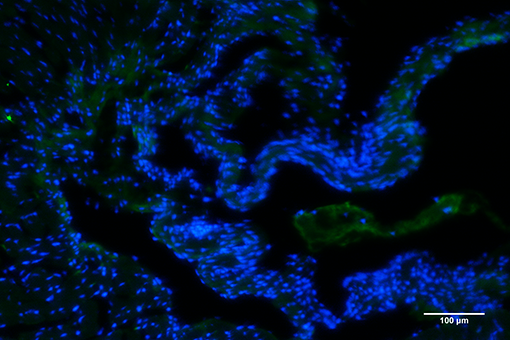

Supplement: Supplementary file 3 [file DataSheet4.ZIP › figure 1-F(p65)/C-2.tif]

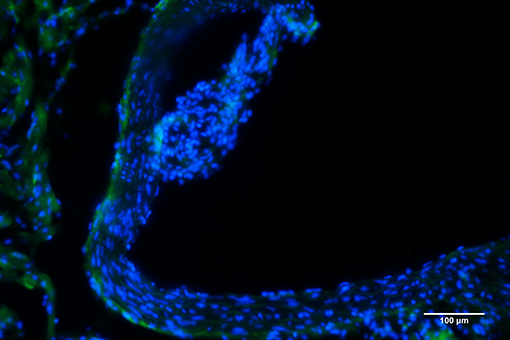

Supplement: Supplementary file 3 [file DataSheet4.ZIP › figure 1-F(p65)/C-3.tif]

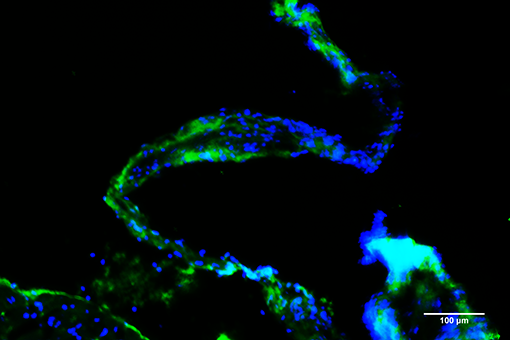

Supplement: Supplementary file 3 [file DataSheet4.ZIP › figure 1-F(p65)/GH-1.tif]

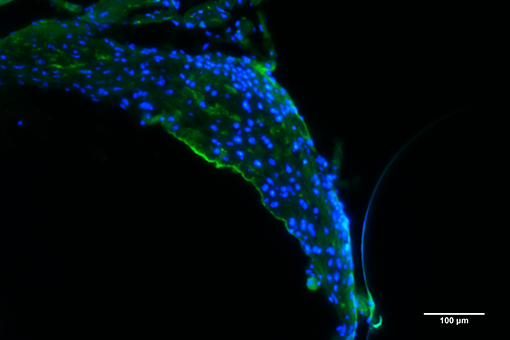

Supplement: Supplementary file 3 [file DataSheet4.ZIP › figure 1-F(p65)/GH-2.tif]

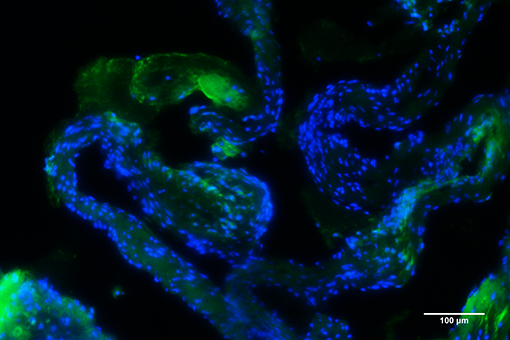

Supplement: Supplementary file 3 [file DataSheet4.ZIP › figure 1-F(p65)/GH-3.tif]

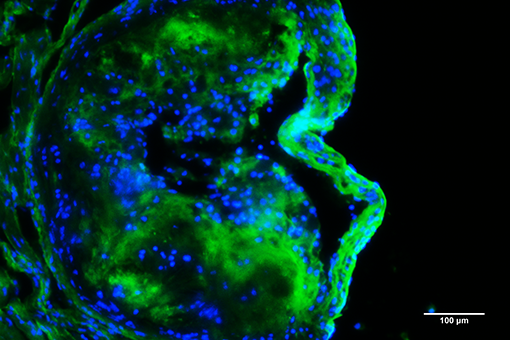

Supplement: Supplementary file 3 [file DataSheet4.ZIP › figure 1-F(p65)/GL-1.tif]

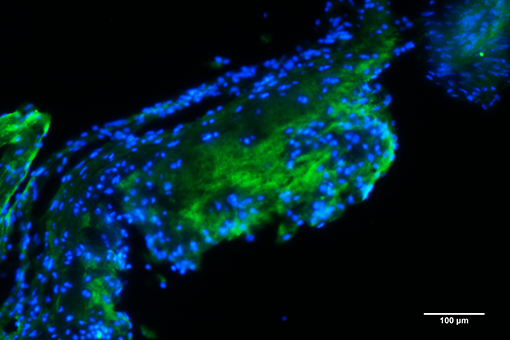

Supplement: Supplementary file 3 [file DataSheet4.ZIP › figure 1-F(p65)/GL-2.tif]

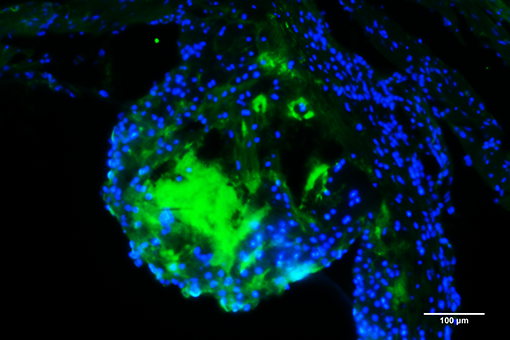

Supplement: Supplementary file 3 [file DataSheet4.ZIP › figure 1-F(p65)/GL-3.tif]

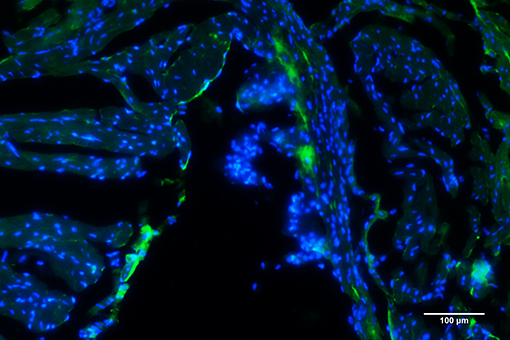

Supplement: Supplementary file 3 [file DataSheet4.ZIP › figure 1-F(p65)/GM-1.tif]

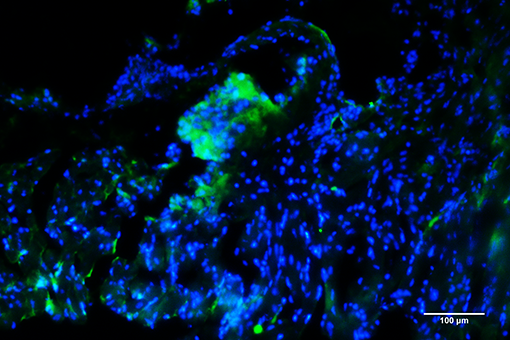

Supplement: Supplementary file 3 [file DataSheet4.ZIP › figure 1-F(p65)/GM-2.tif]

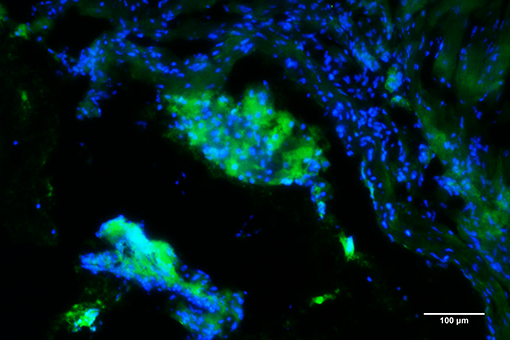

Supplement: Supplementary file 3 [file DataSheet4.ZIP › figure 1-F(p65)/GM-3.tif]

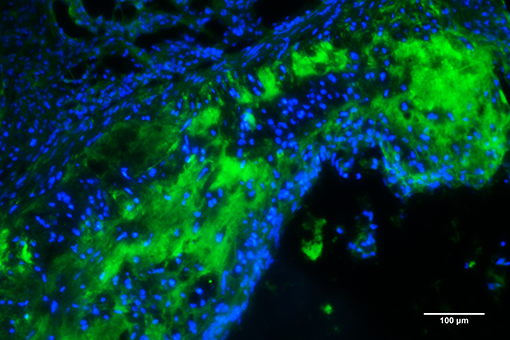

Supplement: Supplementary file 3 [file DataSheet4.ZIP › figure 1-F(p65)/M-1.tif]

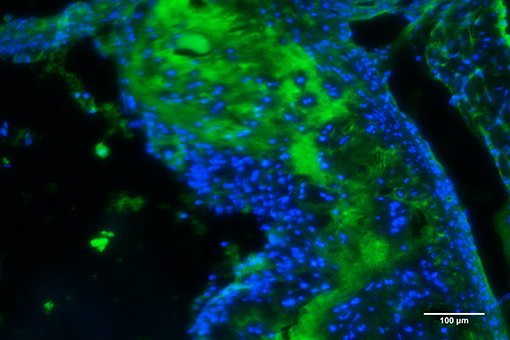

Supplement: Supplementary file 3 [file DataSheet4.ZIP › figure 1-F(p65)/M-2.tif]

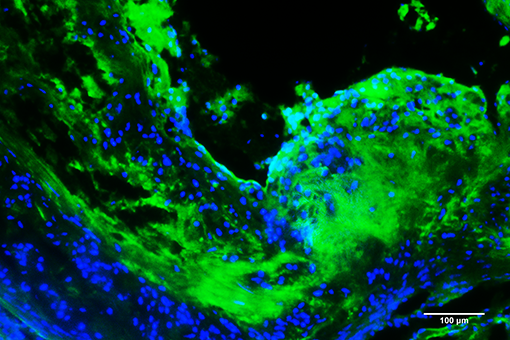

Supplement: Supplementary file 3 [file DataSheet4.ZIP › figure 1-F(p65)/M-3.tif]

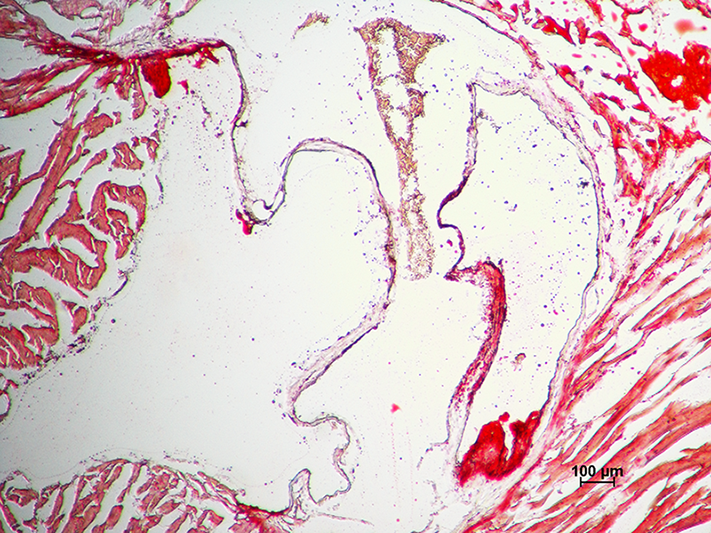

Supplement: Supplementary file 4 [file DataSheet1.ZIP › Oil Red O Staining/ATO-1.tif]

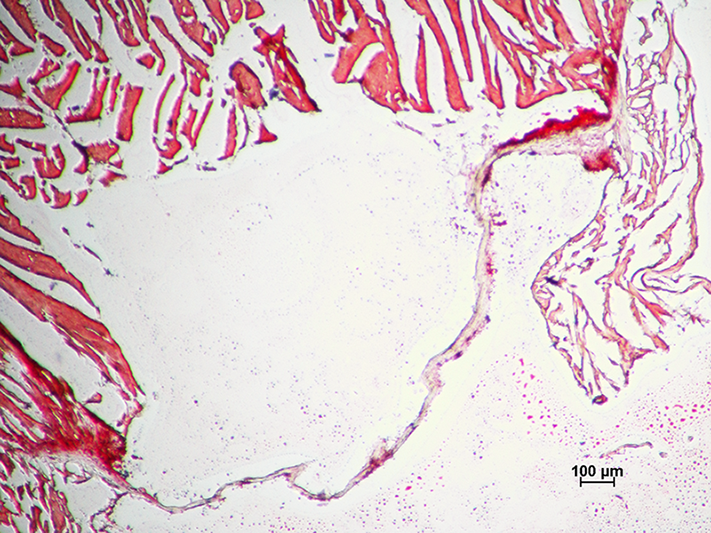

Supplement: Supplementary file 4 [file DataSheet1.ZIP › Oil Red O Staining/ATO-2.tif]

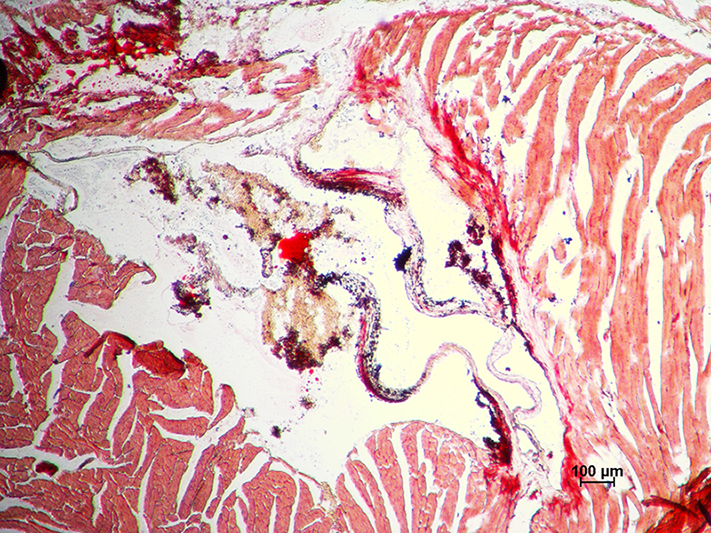

Supplement: Supplementary file 4 [file DataSheet1.ZIP › Oil Red O Staining/ATO-3.tif]

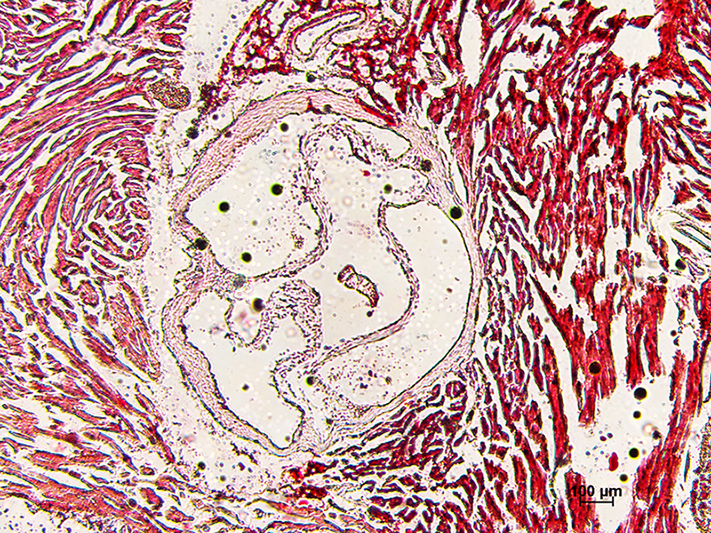

Supplement: Supplementary file 4 [file DataSheet1.ZIP › Oil Red O Staining/C1.tif]

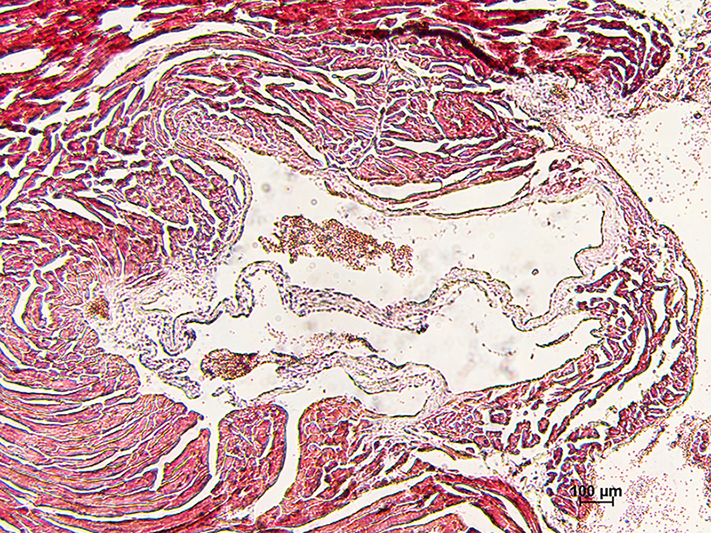

Supplement: Supplementary file 4 [file DataSheet1.ZIP › Oil Red O Staining/C2.tif]

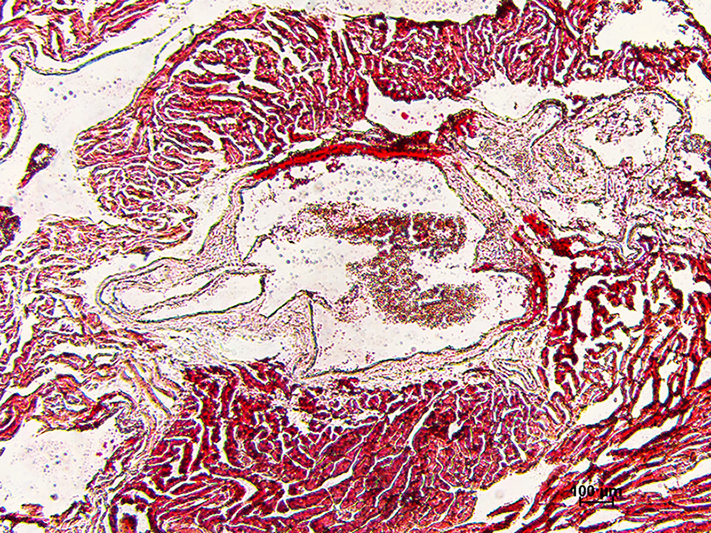

Supplement: Supplementary file 4 [file DataSheet1.ZIP › Oil Red O Staining/C3.tif]

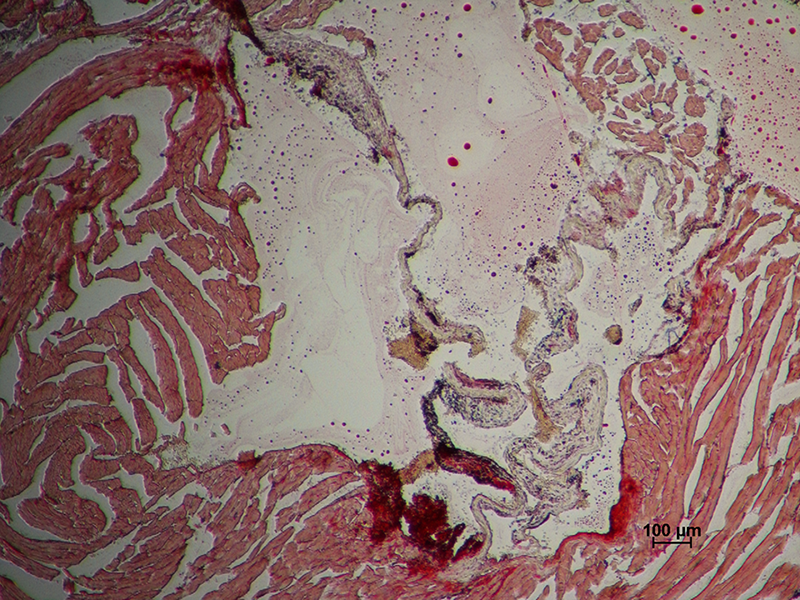

Supplement: Supplementary file 4 [file DataSheet1.ZIP › Oil Red O Staining/FH-1.tif]

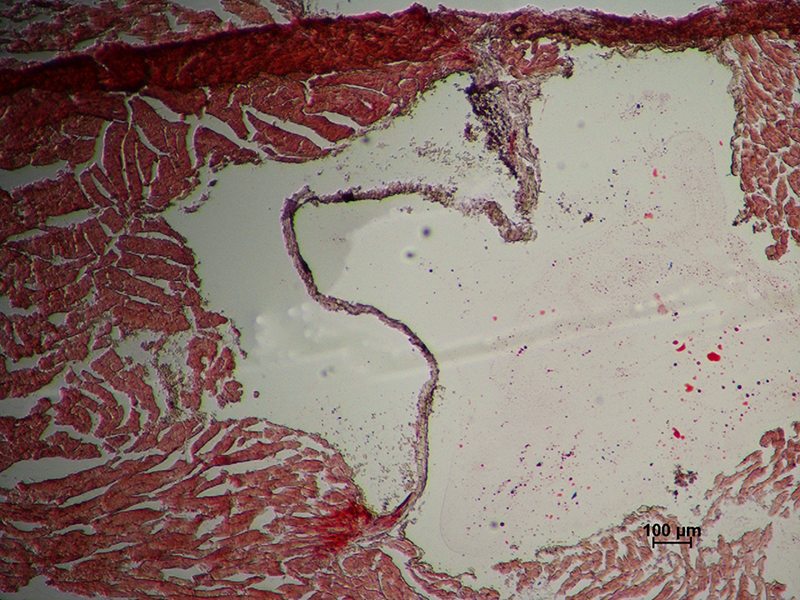

Supplement: Supplementary file 4 [file DataSheet1.ZIP › Oil Red O Staining/FH-2.tif]

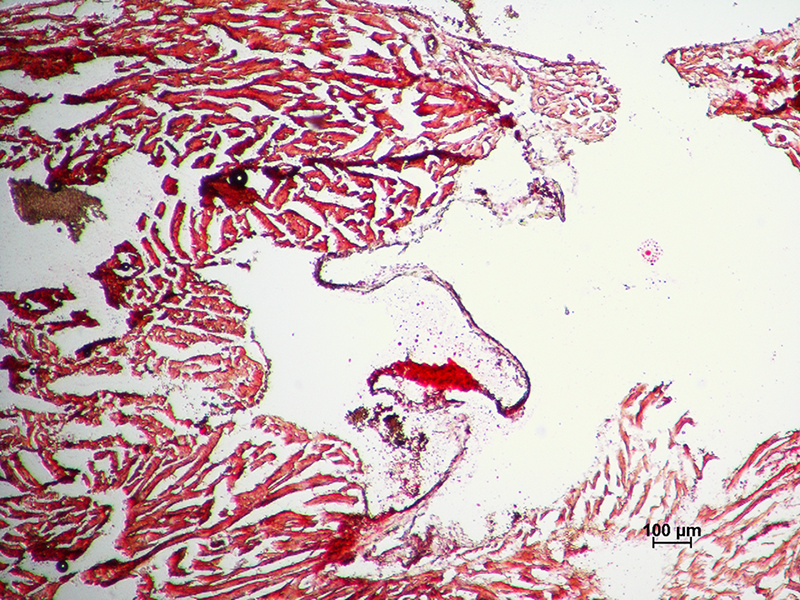

Supplement: Supplementary file 4 [file DataSheet1.ZIP › Oil Red O Staining/FH-3.tif]

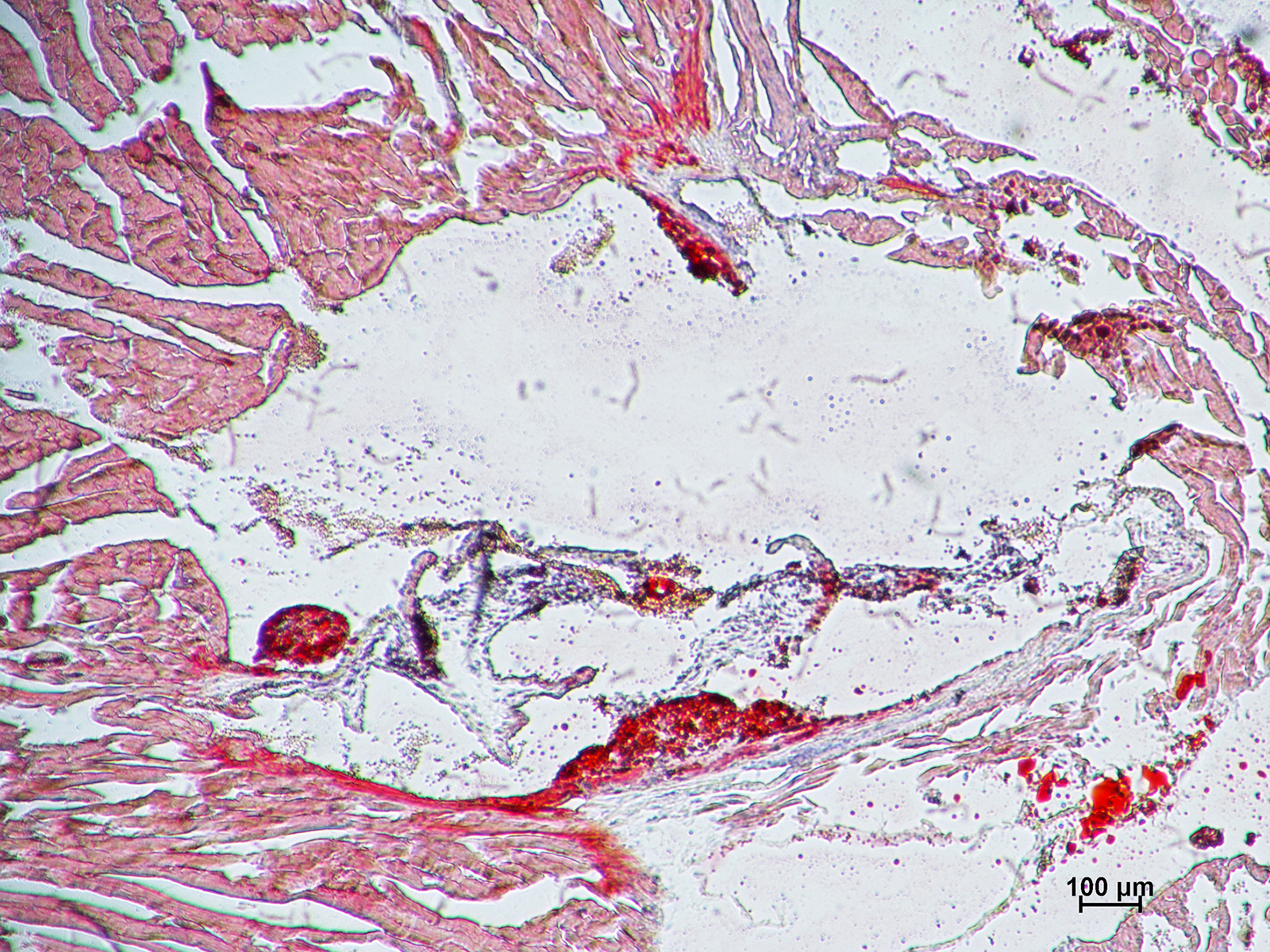

Supplement: Supplementary file 4 [file DataSheet1.ZIP › Oil Red O Staining/FL-1.tif]

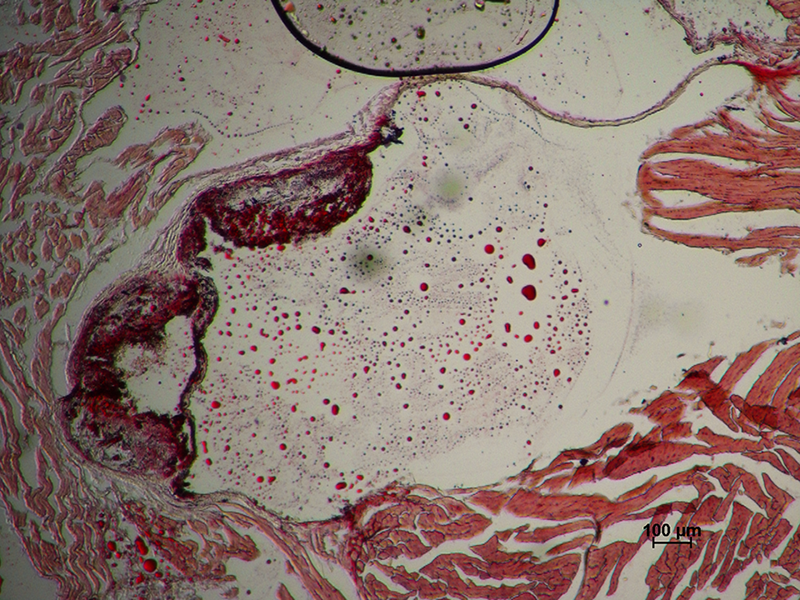

Supplement: Supplementary file 4 [file DataSheet1.ZIP › Oil Red O Staining/FL-2.tif]

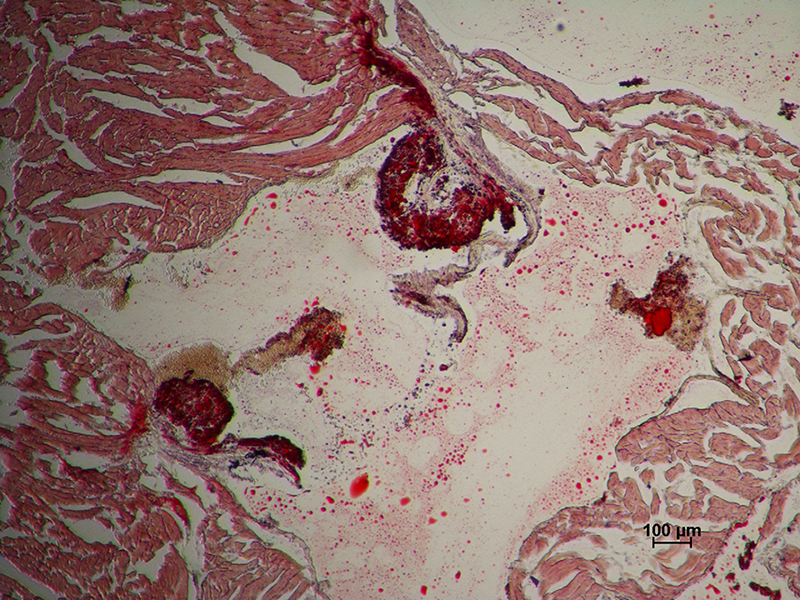

Supplement: Supplementary file 4 [file DataSheet1.ZIP › Oil Red O Staining/FL-3.tif]

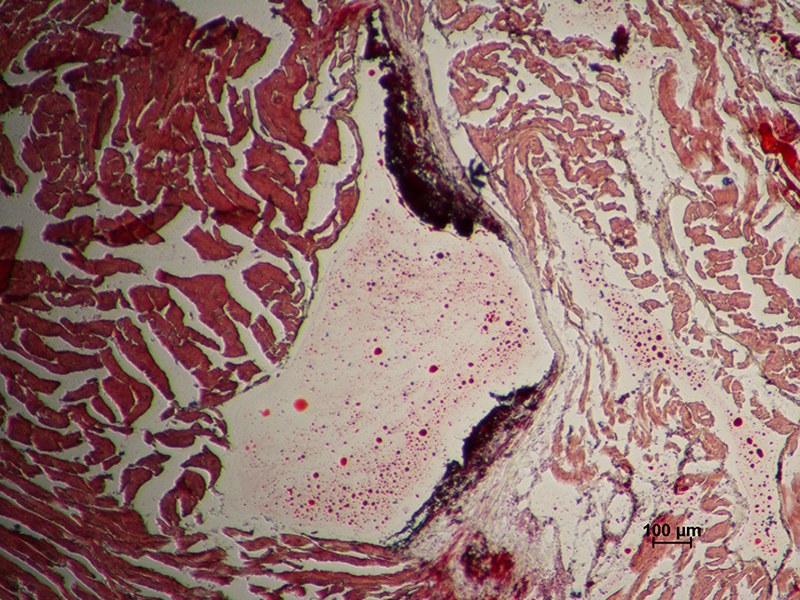

Supplement: Supplementary file 4 [file DataSheet1.ZIP › Oil Red O Staining/FM-1.tif]

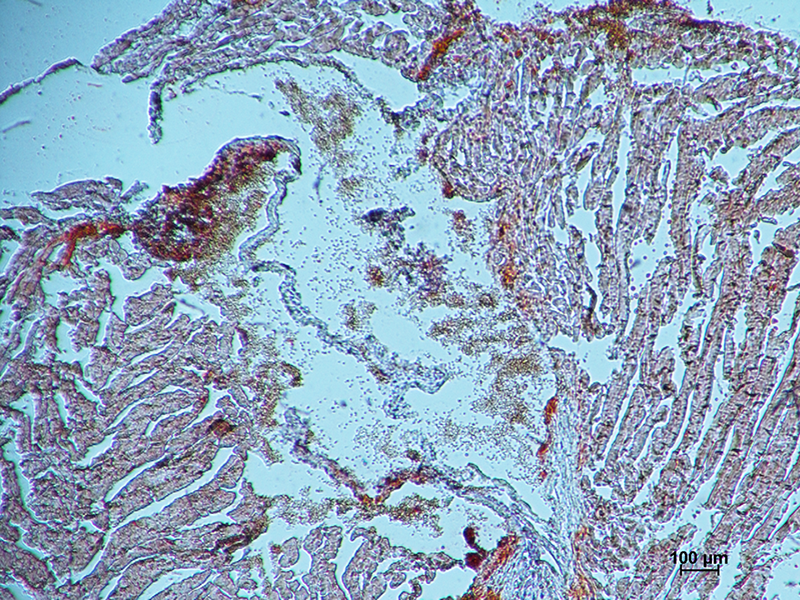

Supplement: Supplementary file 4 [file DataSheet1.ZIP › Oil Red O Staining/FM-2.tif]

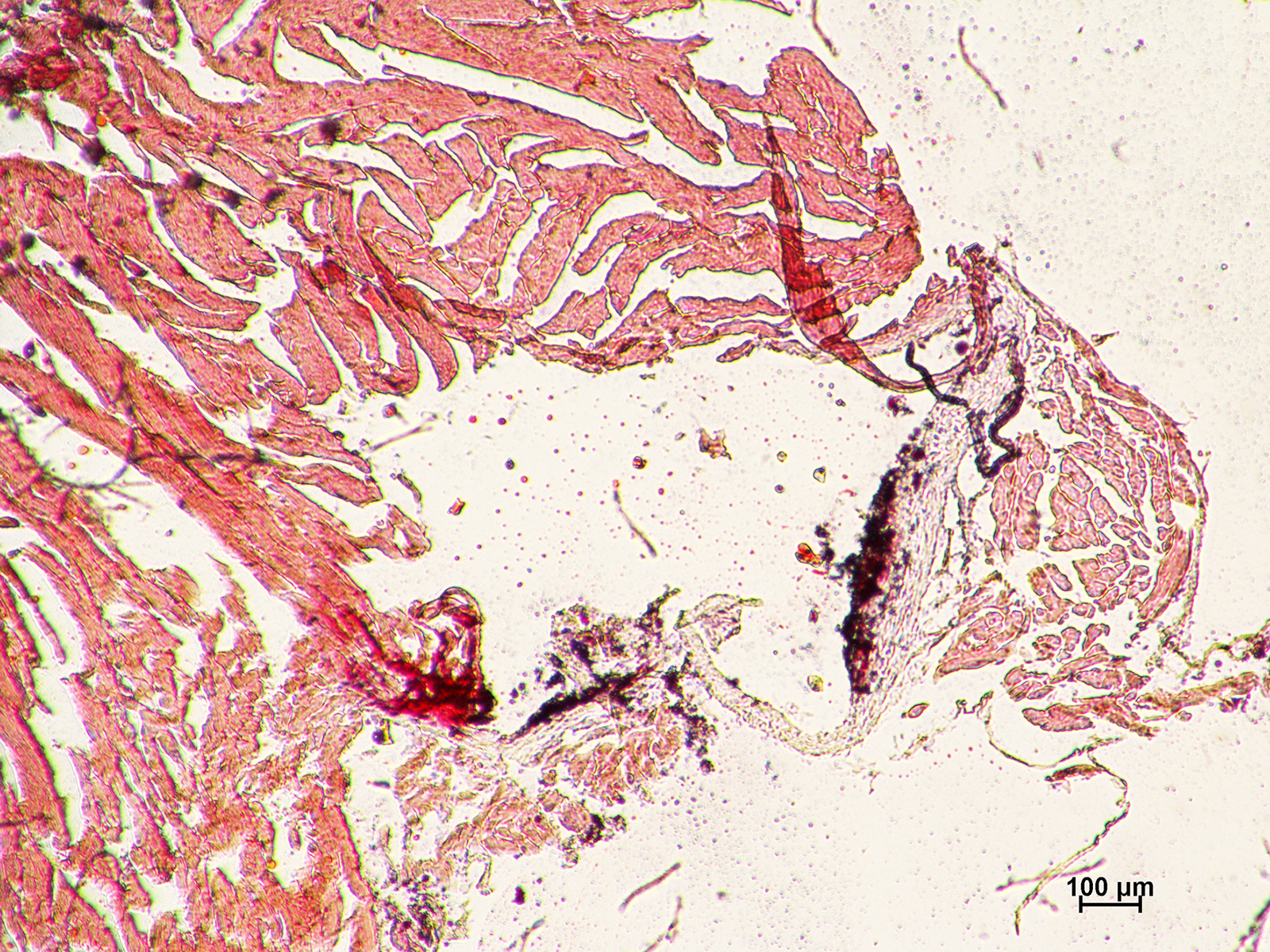

Supplement: Supplementary file 4 [file DataSheet1.ZIP › Oil Red O Staining/FM-3.tif]

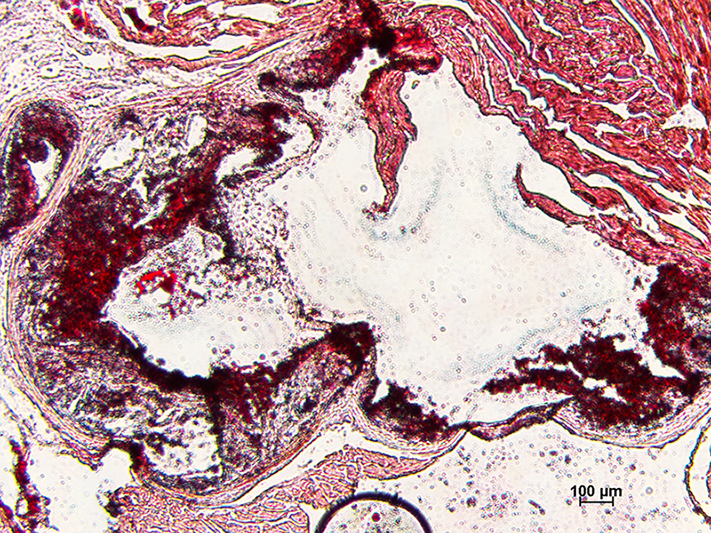

Supplement: Supplementary file 4 [file DataSheet1.ZIP › Oil Red O Staining/M1.tif]

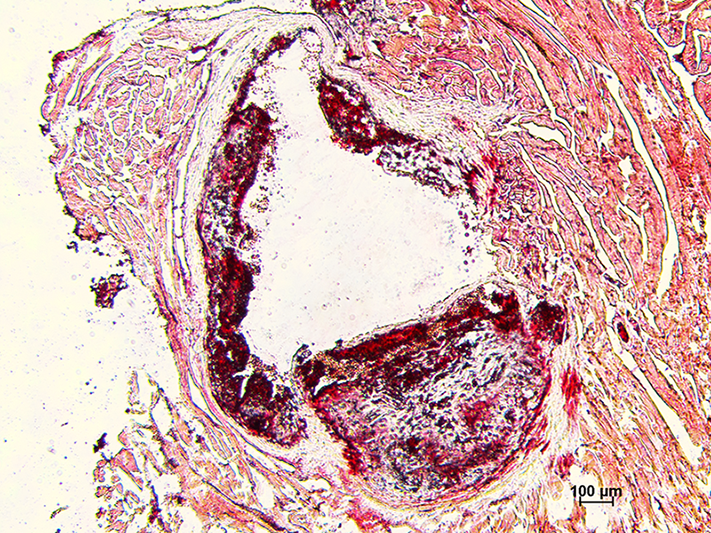

Supplement: Supplementary file 4 [file DataSheet1.ZIP › Oil Red O Staining/M2.tif]

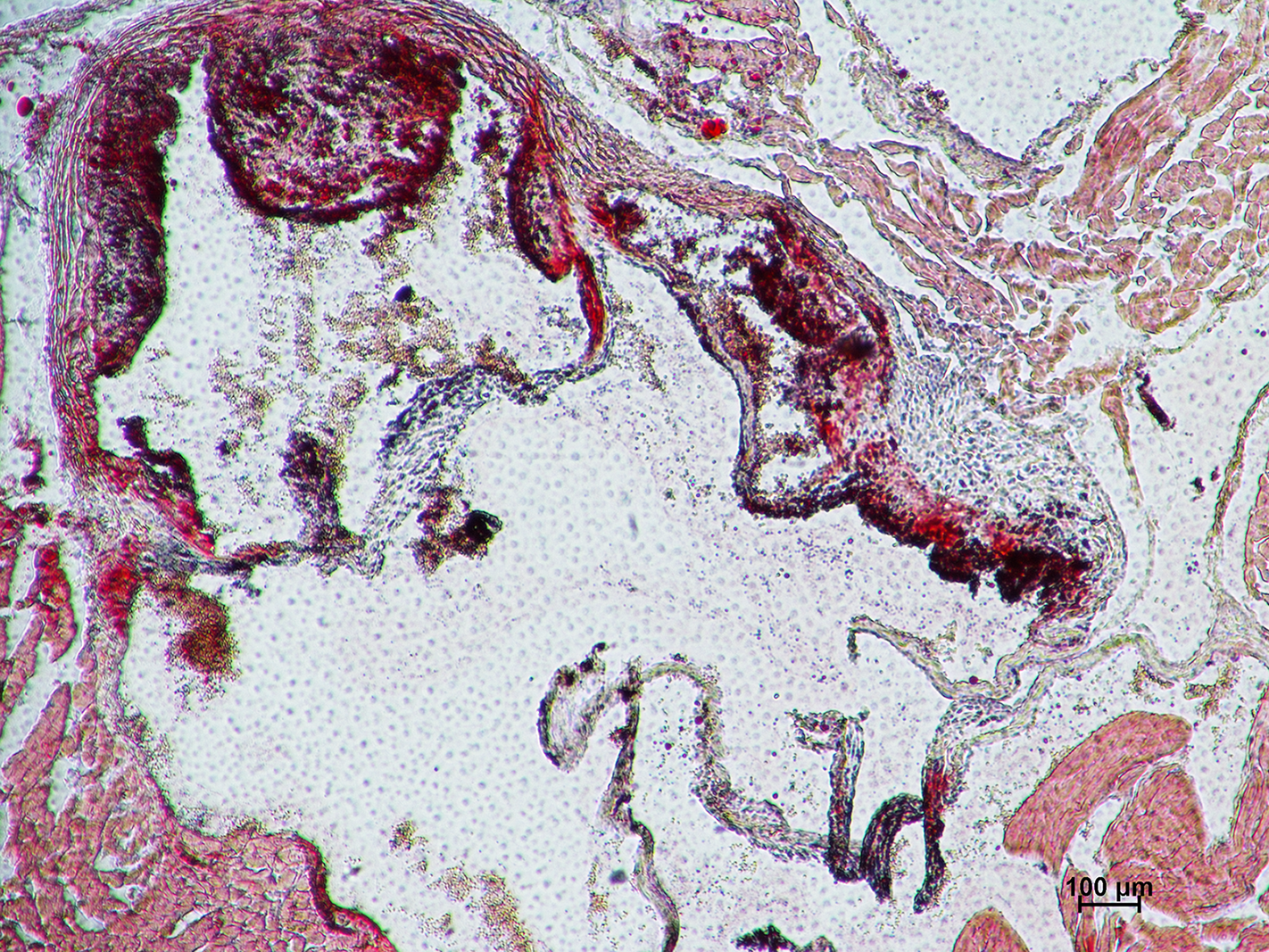

Supplement: Supplementary file 4 [file DataSheet1.ZIP › Oil Red O Staining/M3.tif]

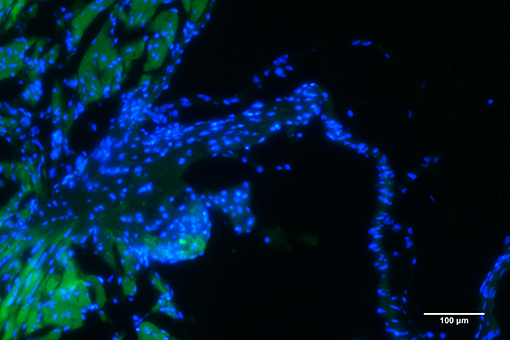

Supplement: Supplementary file 5 [file DataSheet6.ZIP › figure 1-J(caspase 3)/ATO-1.tif]

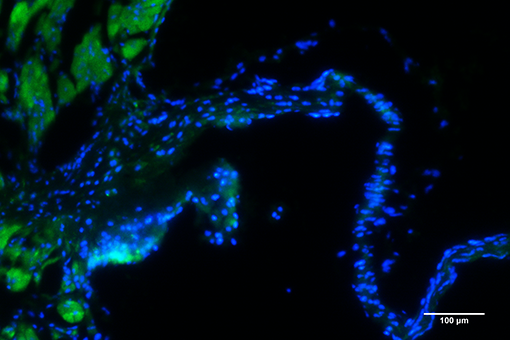

Supplement: Supplementary file 5 [file DataSheet6.ZIP › figure 1-J(caspase 3)/ATO-2.tif]

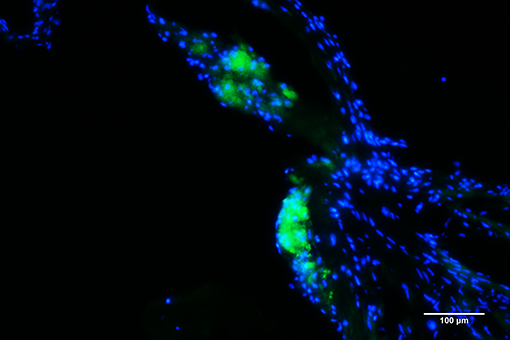

Supplement: Supplementary file 5 [file DataSheet6.ZIP › figure 1-J(caspase 3)/ATO-3.tif]

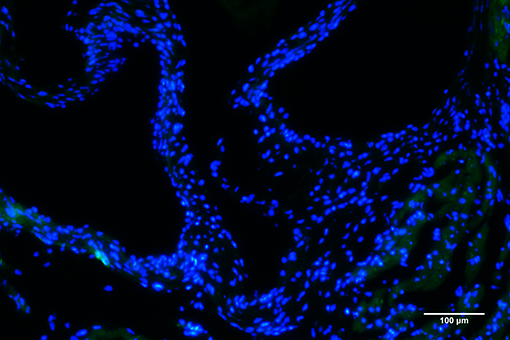

Supplement: Supplementary file 5 [file DataSheet6.ZIP › figure 1-J(caspase 3)/C-1.tif]

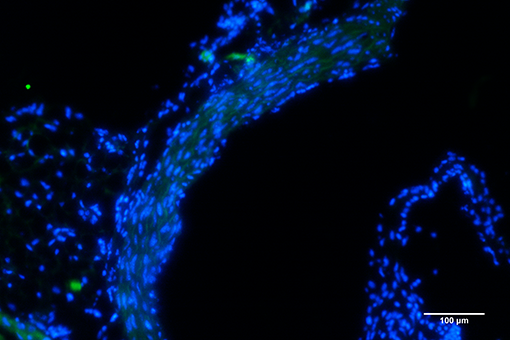

Supplement: Supplementary file 5 [file DataSheet6.ZIP › figure 1-J(caspase 3)/C-2.tif]

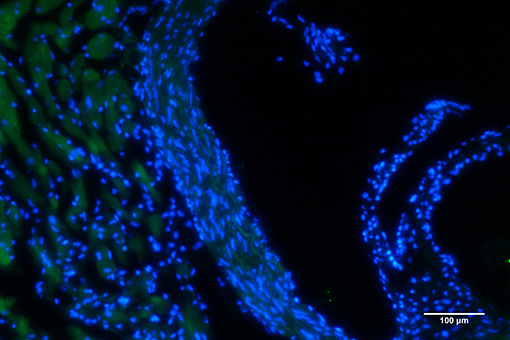

Supplement: Supplementary file 5 [file DataSheet6.ZIP › figure 1-J(caspase 3)/C-3.tif]

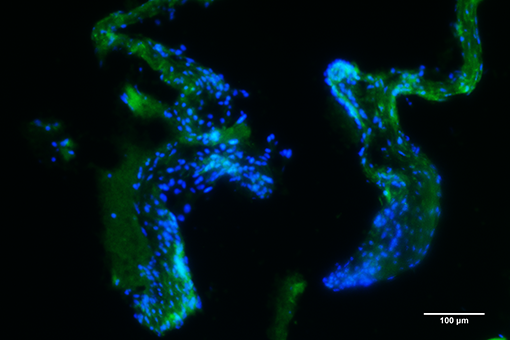

Supplement: Supplementary file 5 [file DataSheet6.ZIP › figure 1-J(caspase 3)/GH-1.tif]

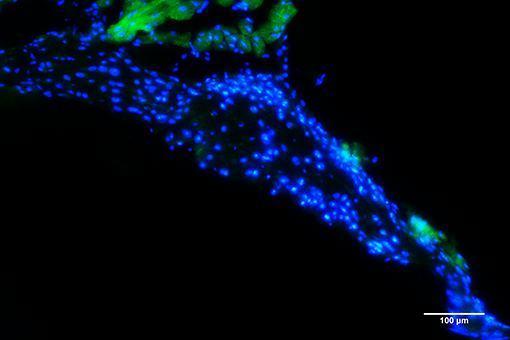

Supplement: Supplementary file 5 [file DataSheet6.ZIP › figure 1-J(caspase 3)/GH-2.tif]

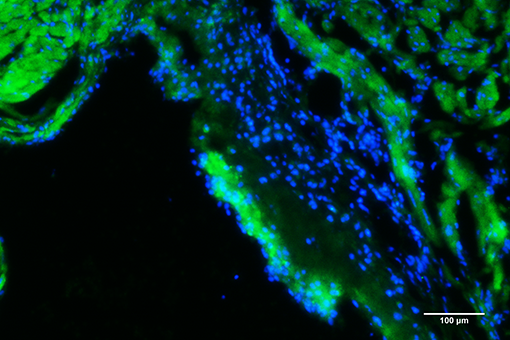

Supplement: Supplementary file 5 [file DataSheet6.ZIP › figure 1-J(caspase 3)/GH-3.tif]

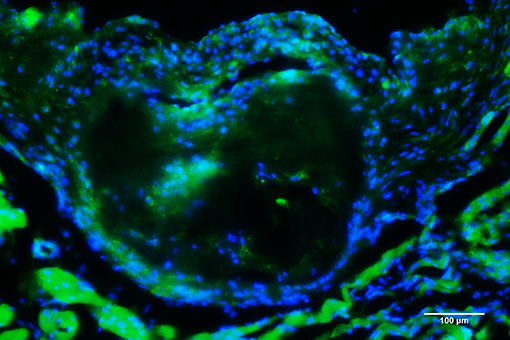

Supplement: Supplementary file 5 [file DataSheet6.ZIP › figure 1-J(caspase 3)/GL-1.tif]

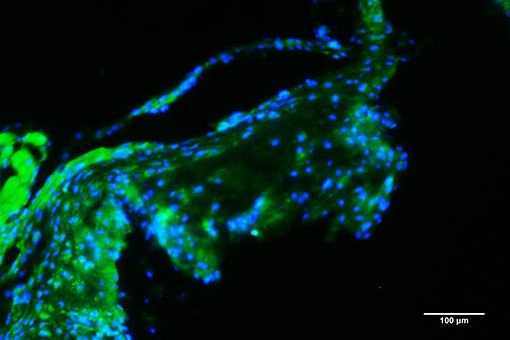

Supplement: Supplementary file 5 [file DataSheet6.ZIP › figure 1-J(caspase 3)/GL-2.tif]

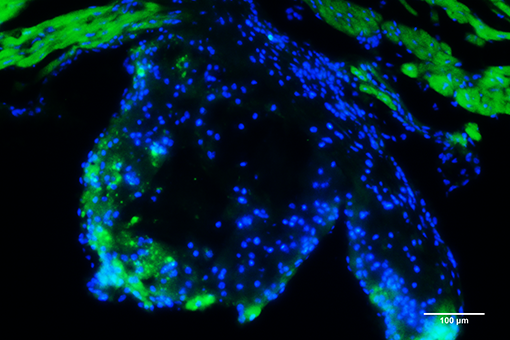

Supplement: Supplementary file 5 [file DataSheet6.ZIP › figure 1-J(caspase 3)/GL-3.tif]

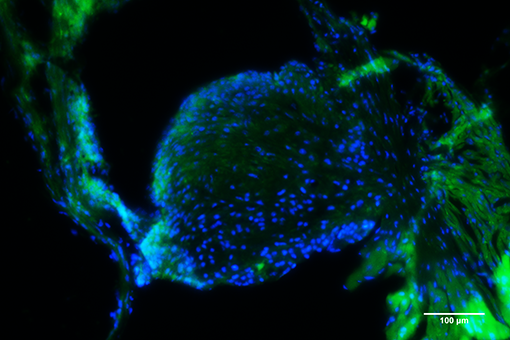

Supplement: Supplementary file 5 [file DataSheet6.ZIP › figure 1-J(caspase 3)/GM-1.tif]

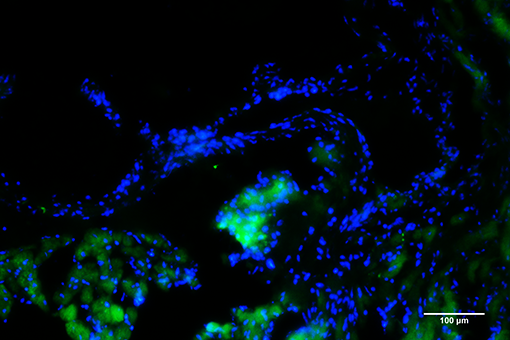

Supplement: Supplementary file 5 [file DataSheet6.ZIP › figure 1-J(caspase 3)/GM-2.tif]

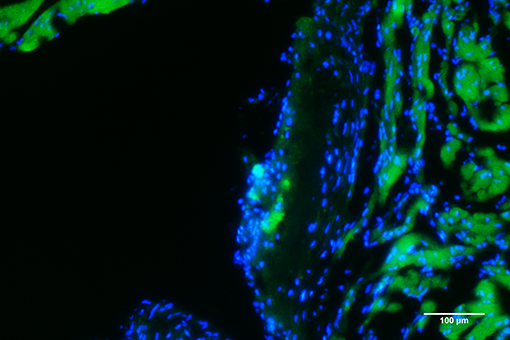

Supplement: Supplementary file 5 [file DataSheet6.ZIP › figure 1-J(caspase 3)/GM-3.tif]

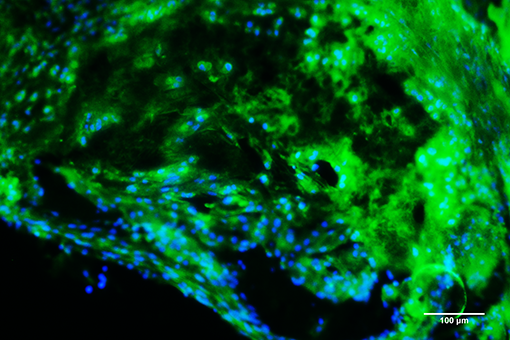

Supplement: Supplementary file 5 [file DataSheet6.ZIP › figure 1-J(caspase 3)/M-1.tif]

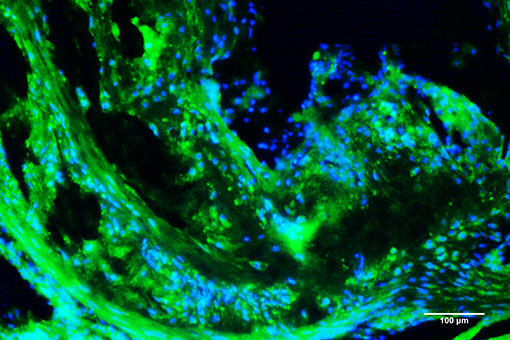

Supplement: Supplementary file 5 [file DataSheet6.ZIP › figure 1-J(caspase 3)/M-2.tif]

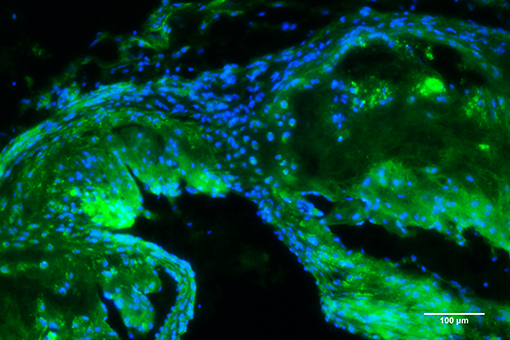

Supplement: Supplementary file 5 [file DataSheet6.ZIP › figure 1-J(caspase 3)/M-3.tif]

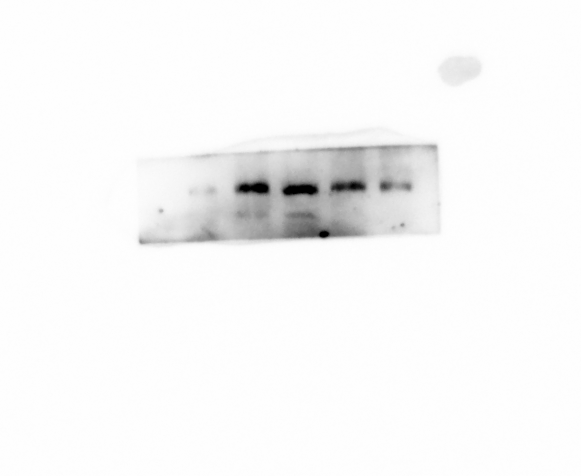

Supplement: Supplementary file 6 [file DataSheet2.ZIP › western blot/figure 2-G/iNOS_1.tif]
