# Supplementary figures and images for: Guanxinkang Decoction Attenuates the Inflammation in Atherosclerosis by Regulating Efferocytosis and MAPKs Signaling Pathway in LDLR−/− Mice and RAW264.7 Cells (part 3 of 3)
Source: Front Pharmacol. 2021 Dec 7;12:731769. doi: 10.3389/fphar.2021.731769 (PMC8688952; doi:10.3389/fphar.2021.731769)

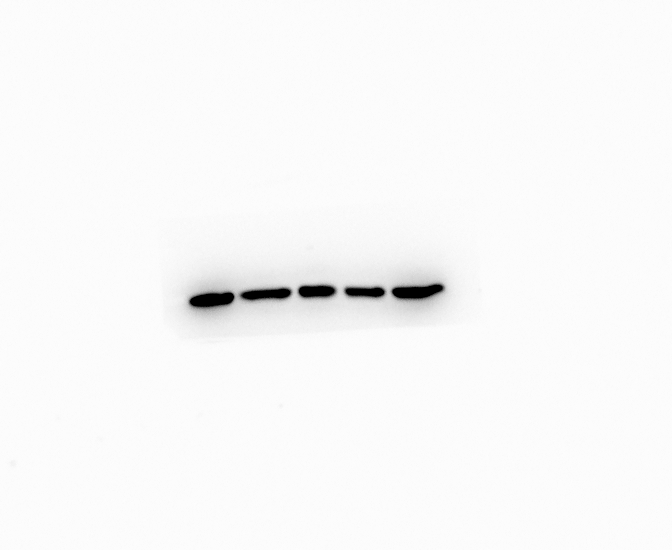

Supplement: Supplementary file 6 [file DataSheet2.ZIP › western blot/figure 7-A/P38-3.tif]

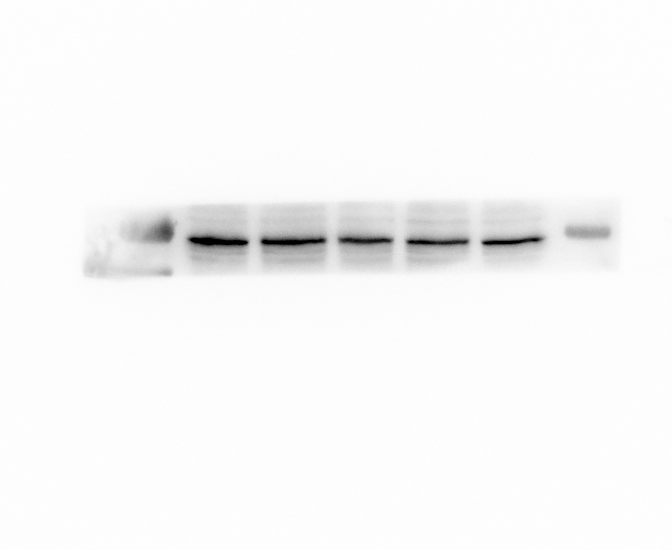

Supplement: Supplementary file 6 [file DataSheet2.ZIP › western blot/figure 7-A/P65-1.tif]

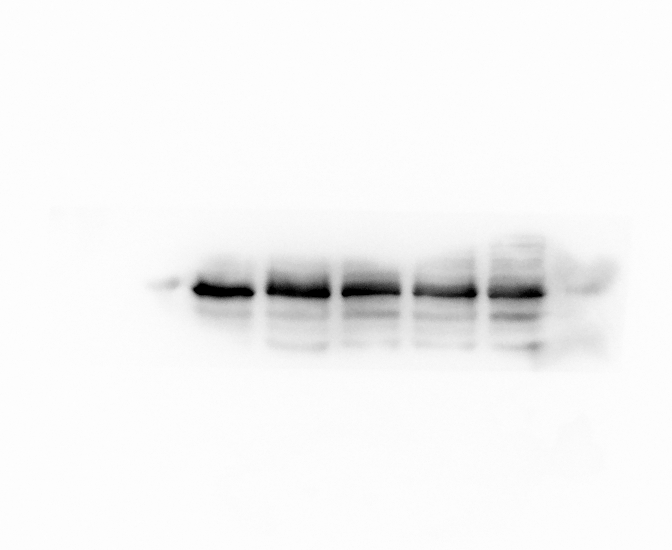

Supplement: Supplementary file 6 [file DataSheet2.ZIP › western blot/figure 7-A/P65-3.tif]

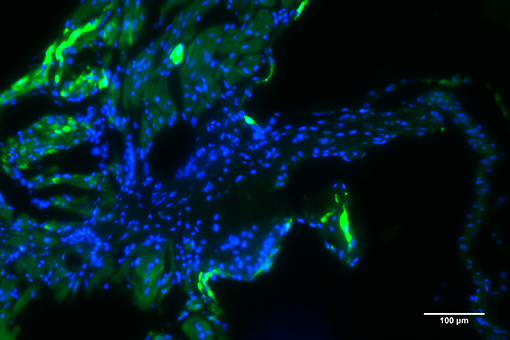

Supplement: Supplementary file 8 [file DataSheet5.ZIP › figure 1-H(SMA)/ATO-1.tif]

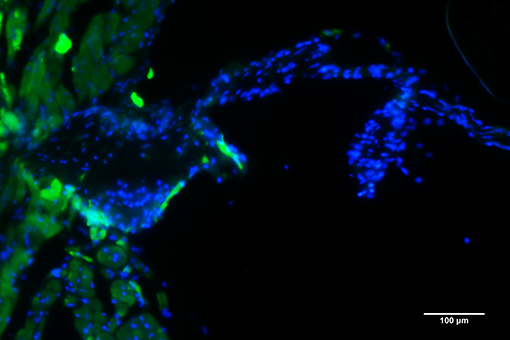

Supplement: Supplementary file 8 [file DataSheet5.ZIP › figure 1-H(SMA)/ATO-2.tif]

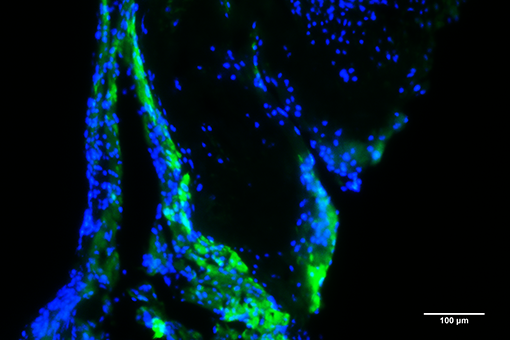

Supplement: Supplementary file 8 [file DataSheet5.ZIP › figure 1-H(SMA)/ATO-3.tif]

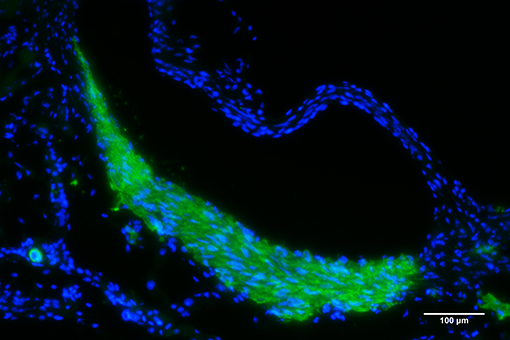

Supplement: Supplementary file 8 [file DataSheet5.ZIP › figure 1-H(SMA)/C-1.tif]

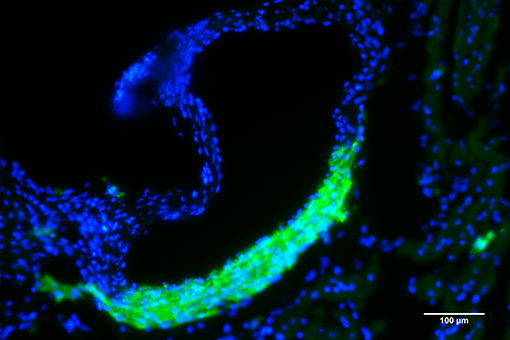

Supplement: Supplementary file 8 [file DataSheet5.ZIP › figure 1-H(SMA)/C-2.tif]

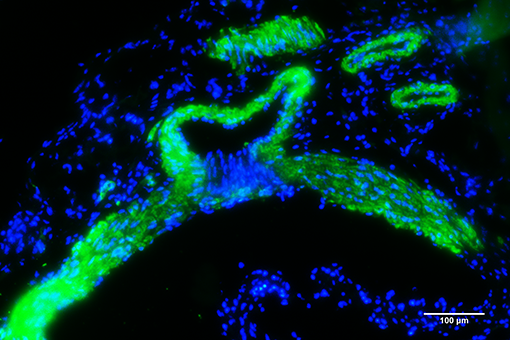

Supplement: Supplementary file 8 [file DataSheet5.ZIP › figure 1-H(SMA)/C-3.tif]

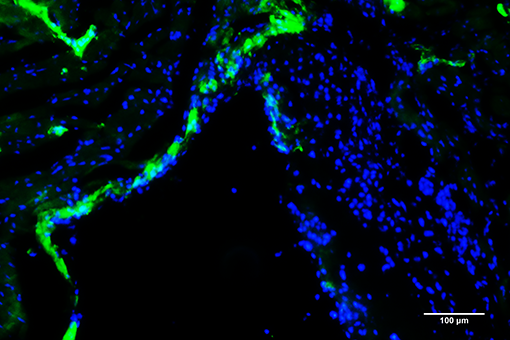

Supplement: Supplementary file 8 [file DataSheet5.ZIP › figure 1-H(SMA)/GH-1.tif]

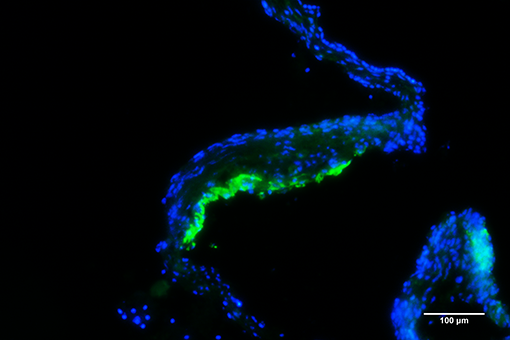

Supplement: Supplementary file 8 [file DataSheet5.ZIP › figure 1-H(SMA)/GH-2.tif]

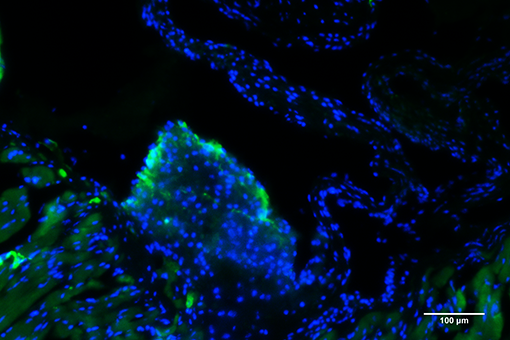

Supplement: Supplementary file 8 [file DataSheet5.ZIP › figure 1-H(SMA)/GH-3.tif]

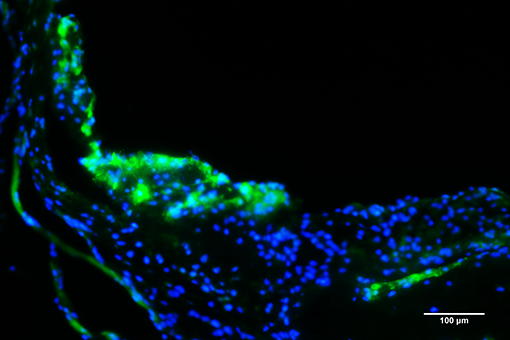

Supplement: Supplementary file 8 [file DataSheet5.ZIP › figure 1-H(SMA)/GL-1.tif]

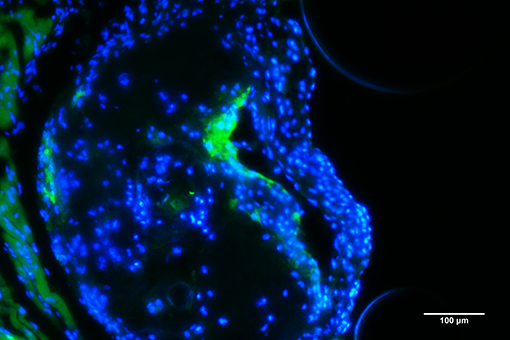

Supplement: Supplementary file 8 [file DataSheet5.ZIP › figure 1-H(SMA)/GL-2.tif]

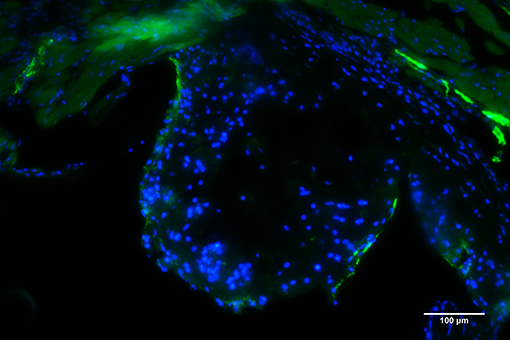

Supplement: Supplementary file 8 [file DataSheet5.ZIP › figure 1-H(SMA)/GL-3.tif]

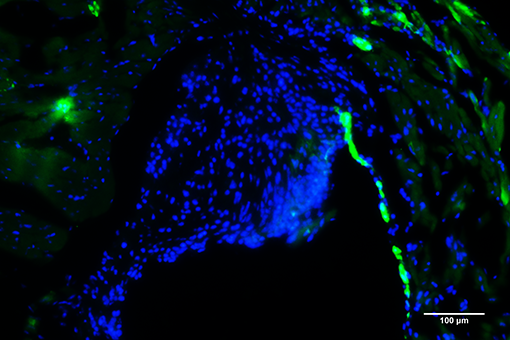

Supplement: Supplementary file 8 [file DataSheet5.ZIP › figure 1-H(SMA)/GM-1.tif]

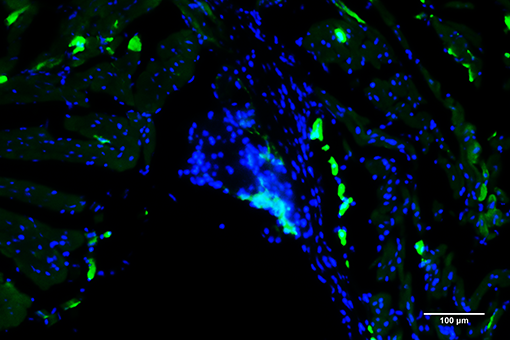

Supplement: Supplementary file 8 [file DataSheet5.ZIP › figure 1-H(SMA)/GM-2.tif]

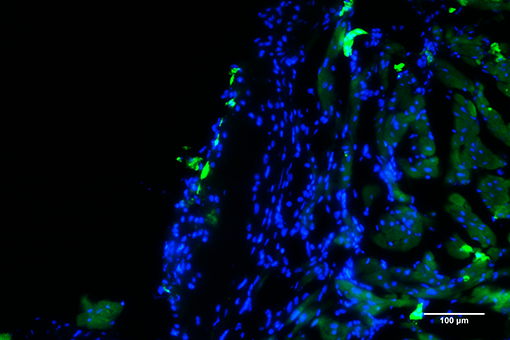

Supplement: Supplementary file 8 [file DataSheet5.ZIP › figure 1-H(SMA)/GM-3.tif]

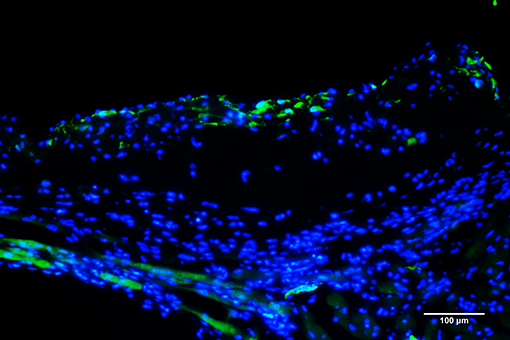

Supplement: Supplementary file 8 [file DataSheet5.ZIP › figure 1-H(SMA)/M-1.tif]

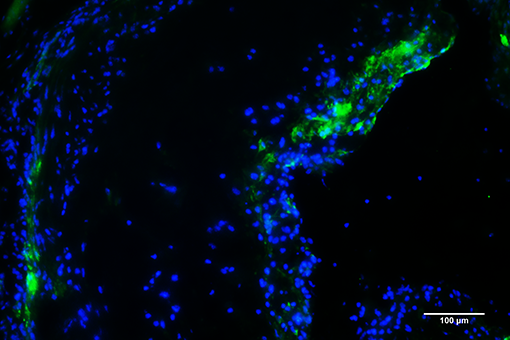

Supplement: Supplementary file 8 [file DataSheet5.ZIP › figure 1-H(SMA)/M-2.tif]

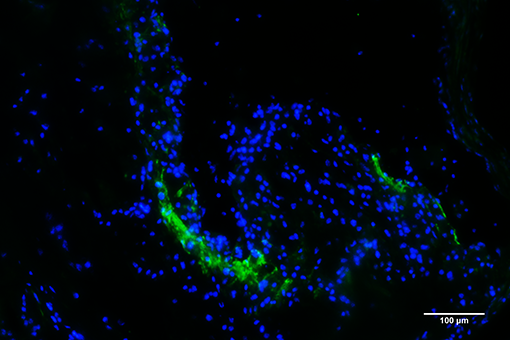

Supplement: Supplementary file 8 [file DataSheet5.ZIP › figure 1-H(SMA)/M-3.tif]
