# Supplementary figures and images for: Guanxinkang Decoction Attenuates the Inflammation in Atherosclerosis by Regulating Efferocytosis and MAPKs Signaling Pathway in LDLR−/− Mice and RAW264.7 Cells (part 2 of 3)
Source: Front Pharmacol. 2021 Dec 7;12:731769. doi: 10.3389/fphar.2021.731769 (PMC8688952; doi:10.3389/fphar.2021.731769)

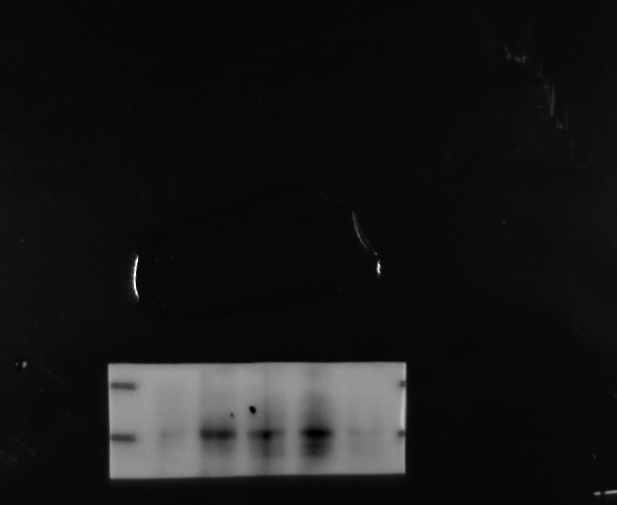

Supplement: Supplementary file 6 [file DataSheet2.ZIP › western blot/figure 2-G/iNOS_2.tif]

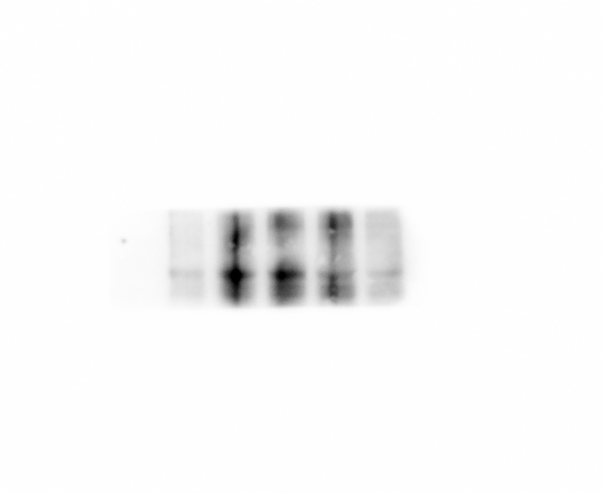

Supplement: Supplementary file 6 [file DataSheet2.ZIP › western blot/figure 2-G/iNOS_3.tif]

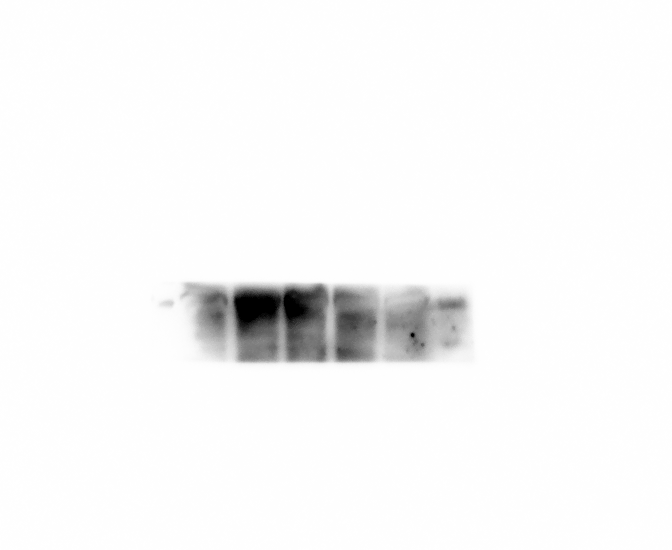

Supplement: Supplementary file 6 [file DataSheet2.ZIP › western blot/figure 2-G/LOX1-1.tif]

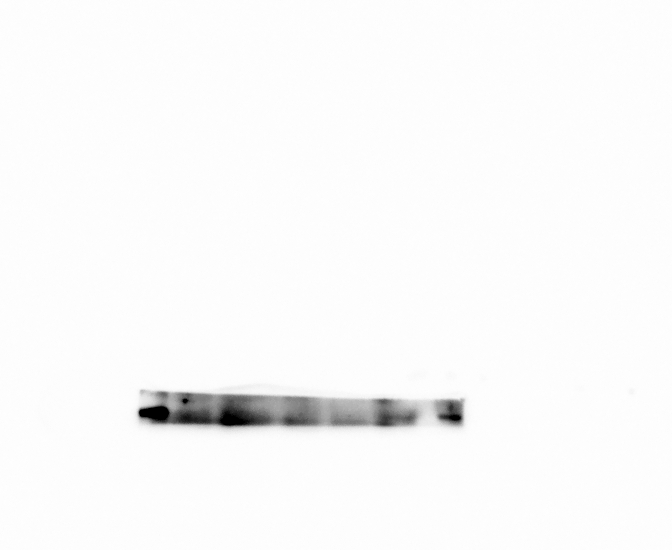

Supplement: Supplementary file 6 [file DataSheet2.ZIP › western blot/figure 2-G/LOX1-2.tif]

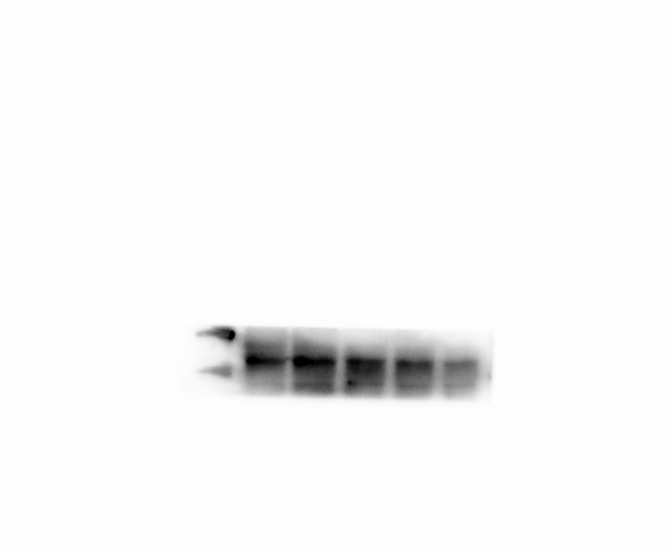

Supplement: Supplementary file 6 [file DataSheet2.ZIP › western blot/figure 2-G/LOX1-3.tif]

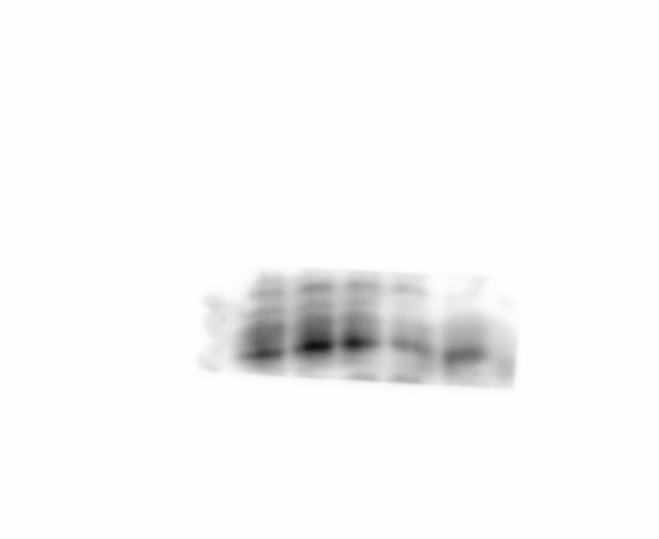

Supplement: Supplementary file 6 [file DataSheet2.ZIP › western blot/figure 2-G/MCP1-1.tif]

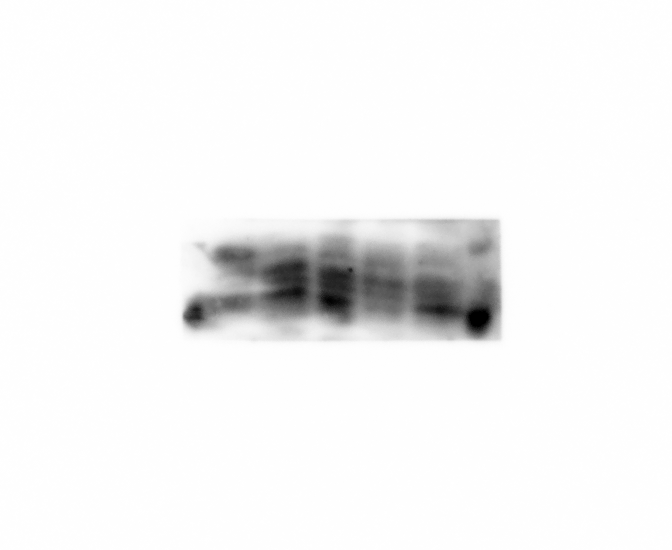

Supplement: Supplementary file 6 [file DataSheet2.ZIP › western blot/figure 2-G/MCP1-2.tif]

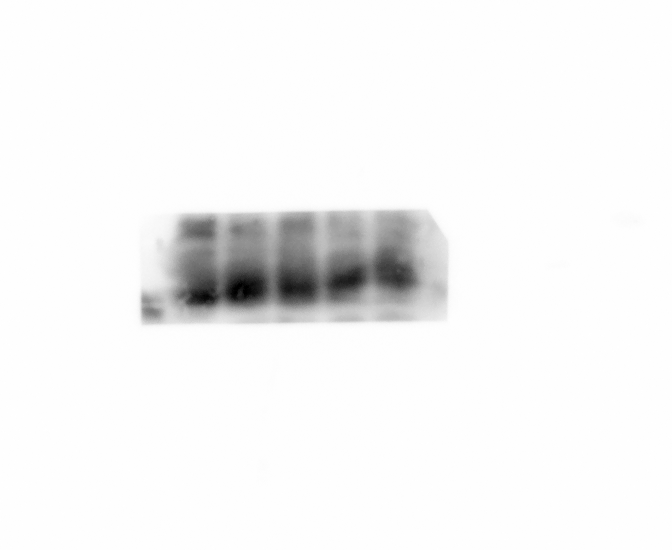

Supplement: Supplementary file 6 [file DataSheet2.ZIP › western blot/figure 2-G/MCP1-3.tif]

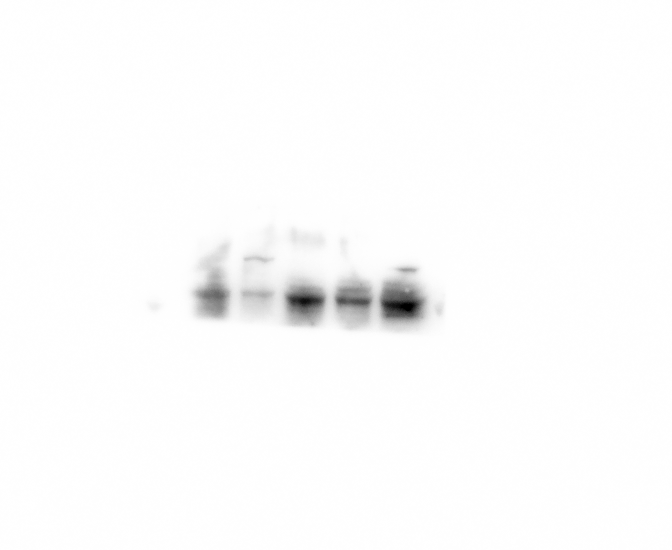

Supplement: Supplementary file 6 [file DataSheet2.ZIP › western blot/figure 3-D/AXL-1.tif]

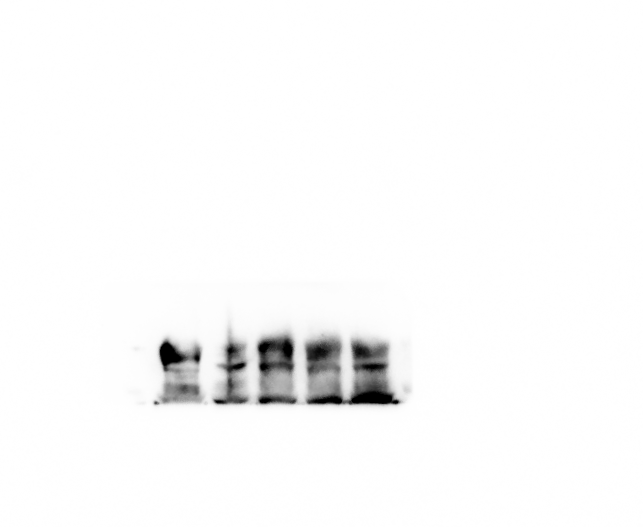

Supplement: Supplementary file 6 [file DataSheet2.ZIP › western blot/figure 3-D/AXL-2.tif]

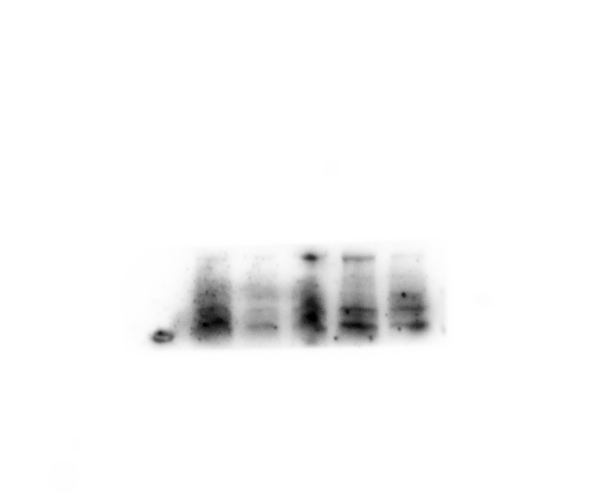

Supplement: Supplementary file 6 [file DataSheet2.ZIP › western blot/figure 3-D/AXL-3.tif]

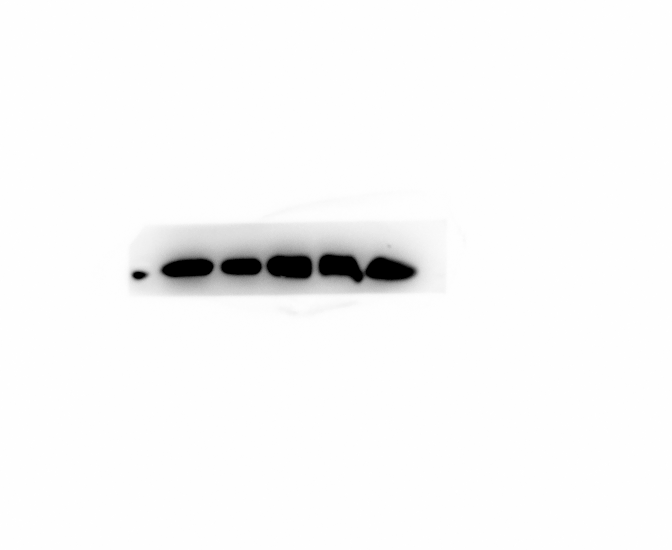

Supplement: Supplementary file 6 [file DataSheet2.ZIP › western blot/figure 3-D/GAPDH-1.tif]

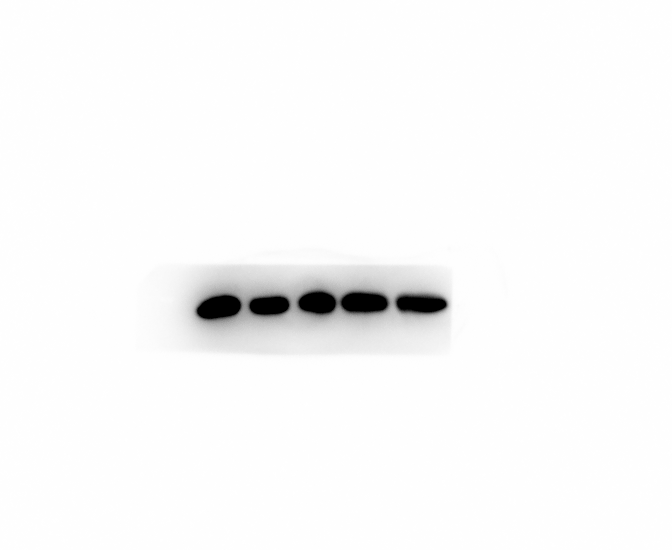

Supplement: Supplementary file 6 [file DataSheet2.ZIP › western blot/figure 3-D/GAPDH-2.tif]

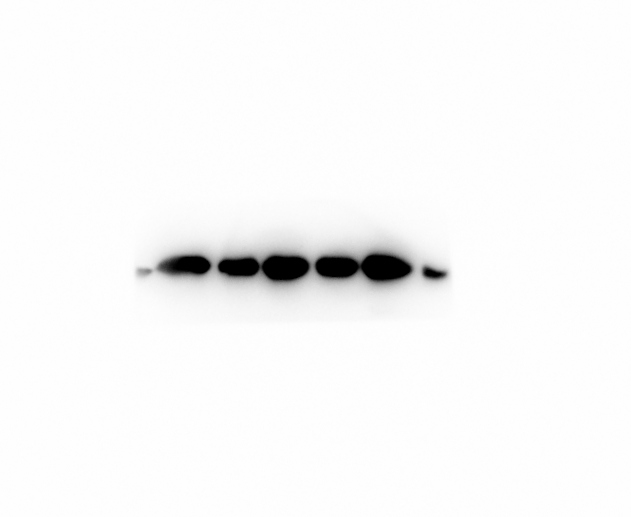

Supplement: Supplementary file 6 [file DataSheet2.ZIP › western blot/figure 3-D/GAPDH-3.tif]

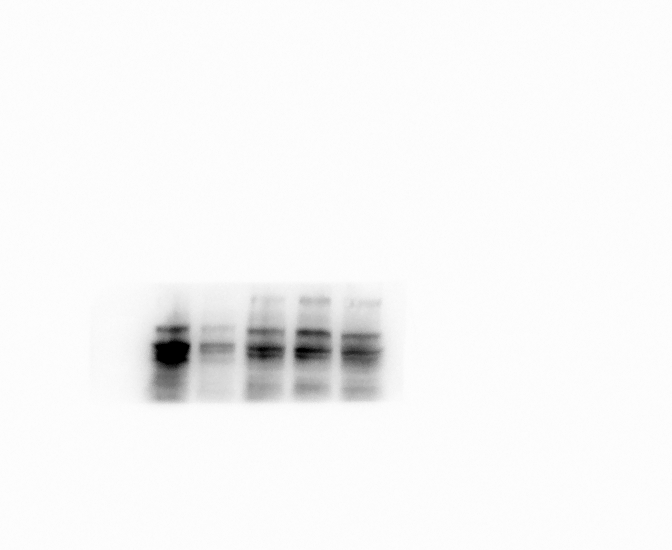

Supplement: Supplementary file 6 [file DataSheet2.ZIP › western blot/figure 3-D/MERTK-1.tif]

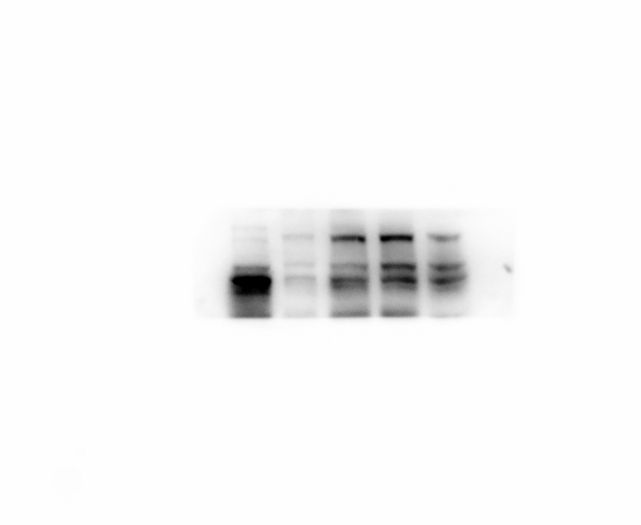

Supplement: Supplementary file 6 [file DataSheet2.ZIP › western blot/figure 3-D/MERTK-2.tif]

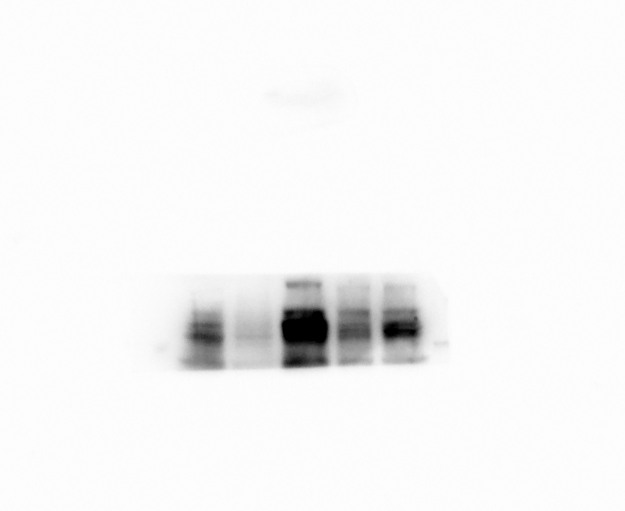

Supplement: Supplementary file 6 [file DataSheet2.ZIP › western blot/figure 3-D/MERTK-3.tif]

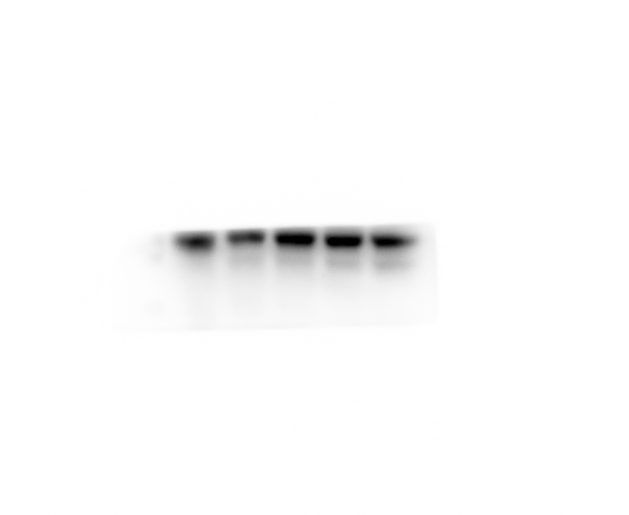

Supplement: Supplementary file 6 [file DataSheet2.ZIP › western blot/figure 3-D/MFGE8-1.tif]

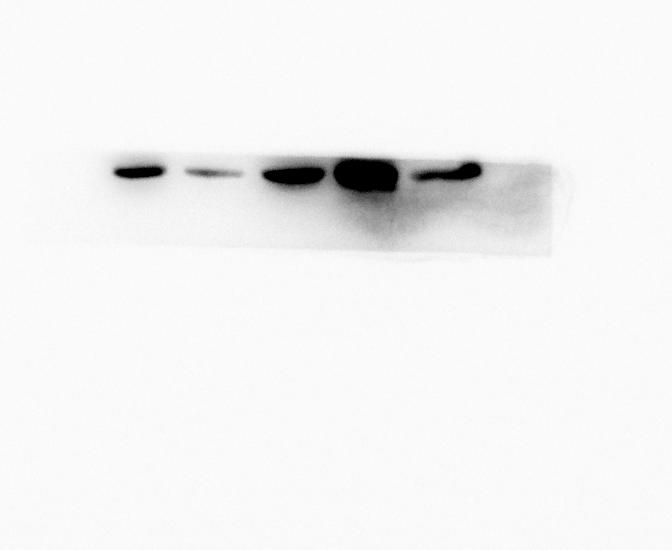

Supplement: Supplementary file 6 [file DataSheet2.ZIP › western blot/figure 3-D/MFGE8-2.tif]

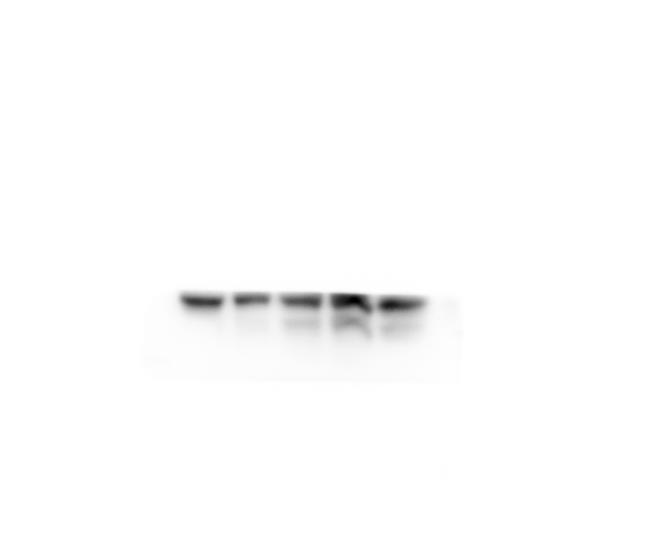

Supplement: Supplementary file 6 [file DataSheet2.ZIP › western blot/figure 3-D/MFGE8-3.tif]

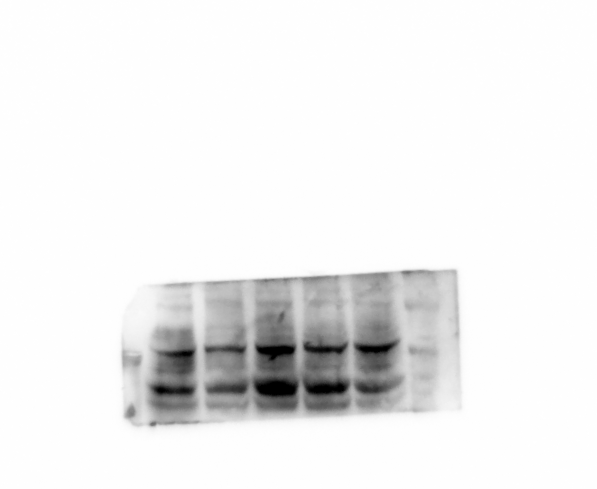

Supplement: Supplementary file 6 [file DataSheet2.ZIP › western blot/figure 3-D/TYRO-1.tif]

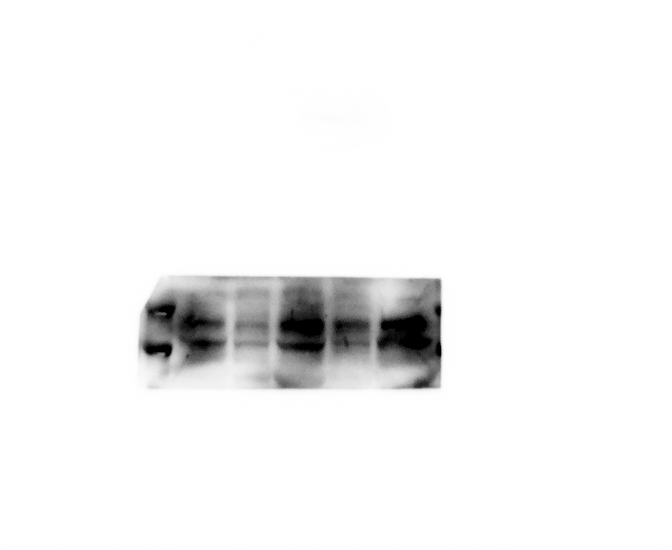

Supplement: Supplementary file 6 [file DataSheet2.ZIP › western blot/figure 3-D/TYRO-2.tif]

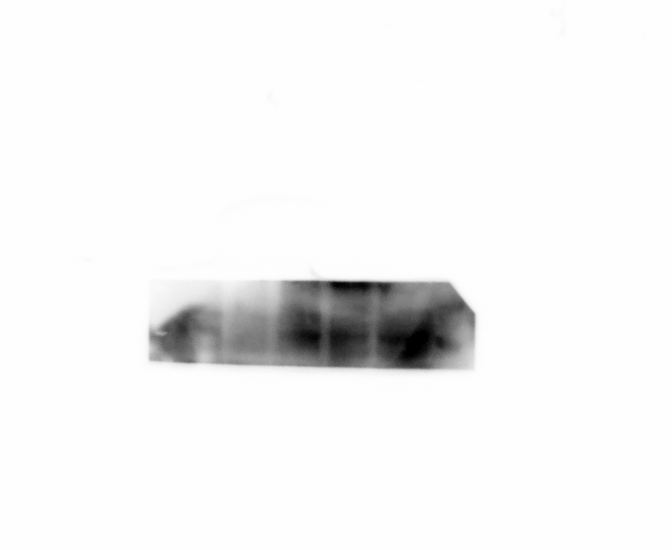

Supplement: Supplementary file 6 [file DataSheet2.ZIP › western blot/figure 3-D/TYRO-3.tif]

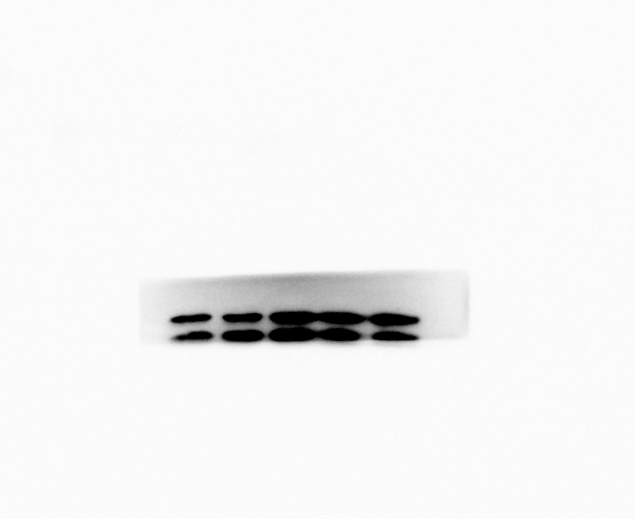

Supplement: Supplementary file 6 [file DataSheet2.ZIP › western blot/figure 4-A/ERK_1.tif]

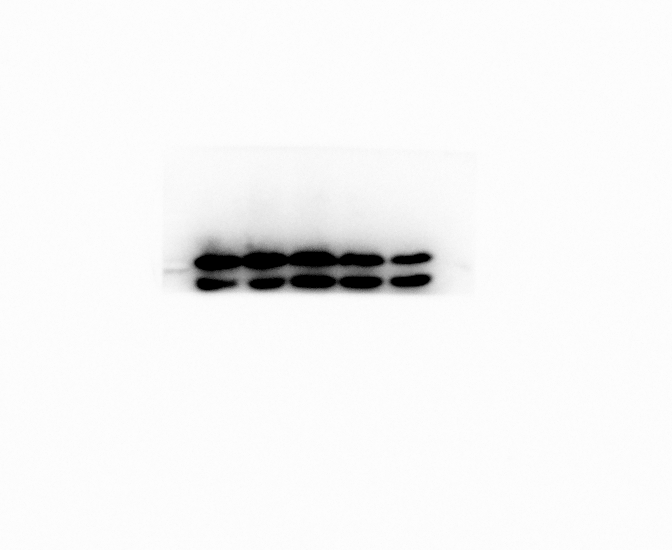

Supplement: Supplementary file 6 [file DataSheet2.ZIP › western blot/figure 4-A/ERK_2.tif]

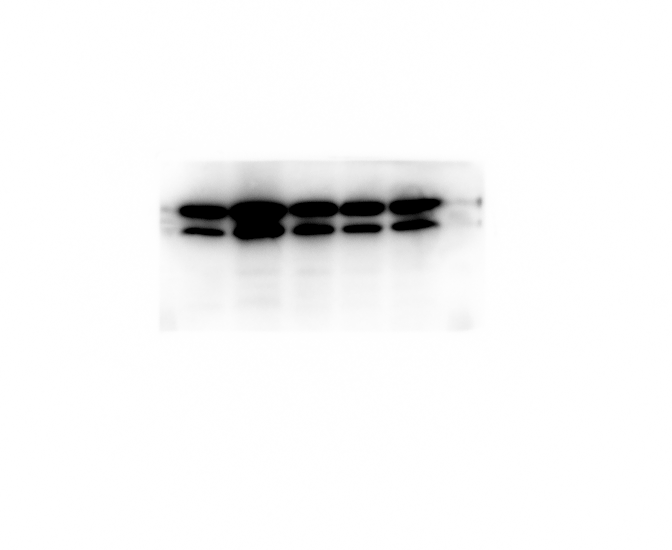

Supplement: Supplementary file 6 [file DataSheet2.ZIP › western blot/figure 4-A/ERK_3.tif]

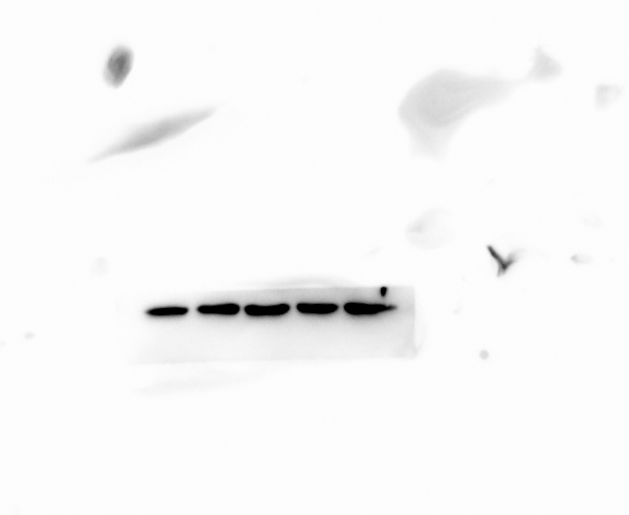

Supplement: Supplementary file 6 [file DataSheet2.ZIP › western blot/figure 4-A/GAPDH_1.tif]

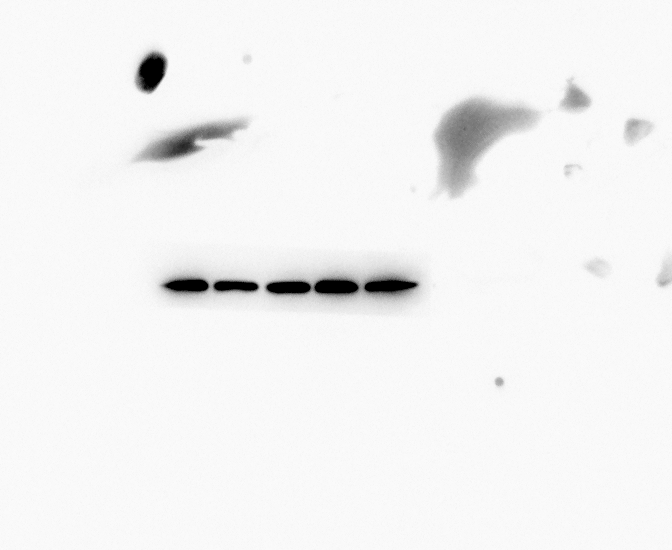

Supplement: Supplementary file 6 [file DataSheet2.ZIP › western blot/figure 4-A/GAPDH_2.tif]

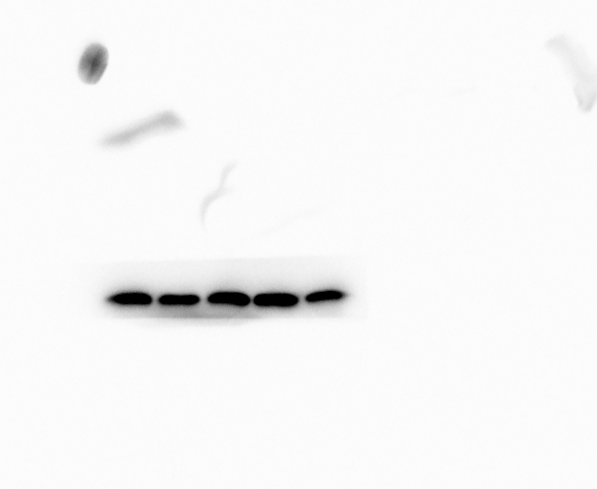

Supplement: Supplementary file 6 [file DataSheet2.ZIP › western blot/figure 4-A/GAPDH_3.tif]

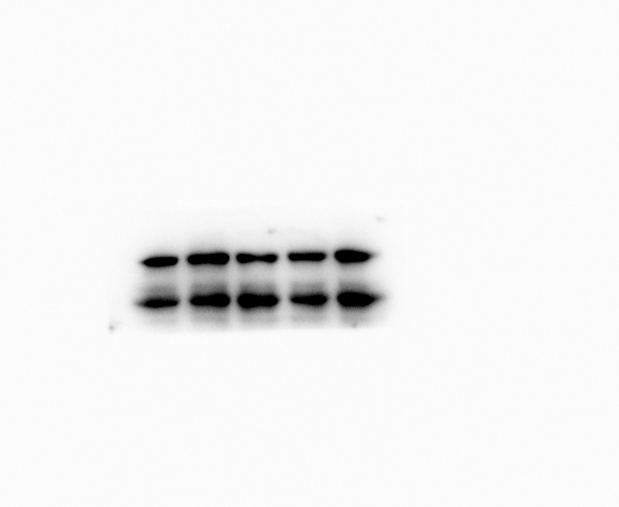

Supplement: Supplementary file 6 [file DataSheet2.ZIP › western blot/figure 4-A/JNK_1.tif]

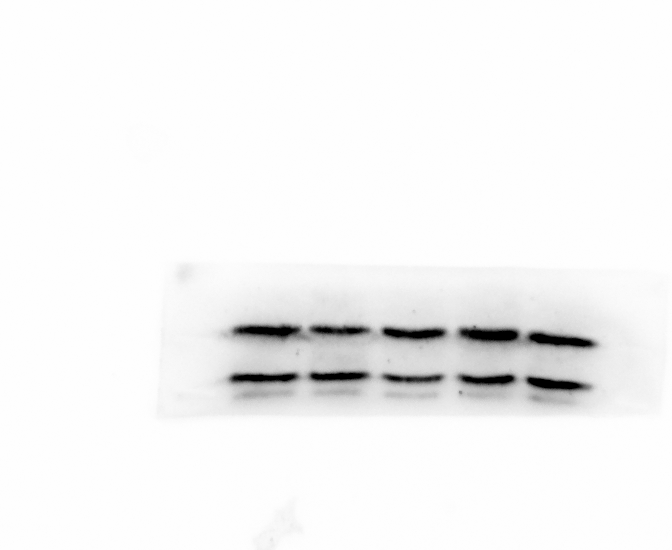

Supplement: Supplementary file 6 [file DataSheet2.ZIP › western blot/figure 4-A/JNK_2.tif]

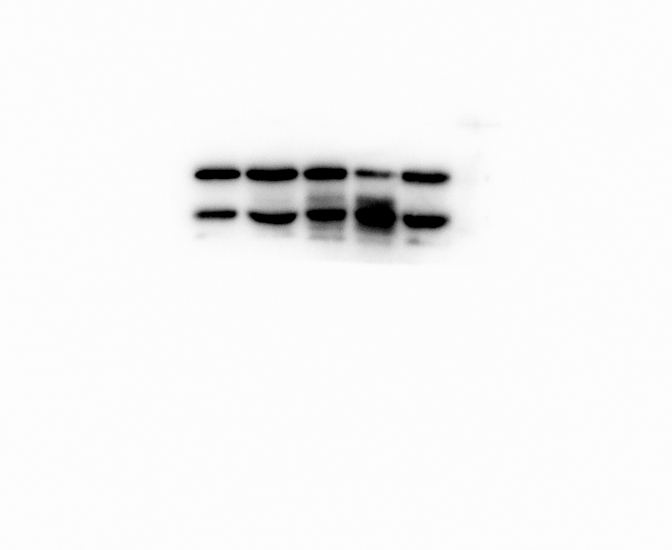

Supplement: Supplementary file 6 [file DataSheet2.ZIP › western blot/figure 4-A/JNK_3.tif]

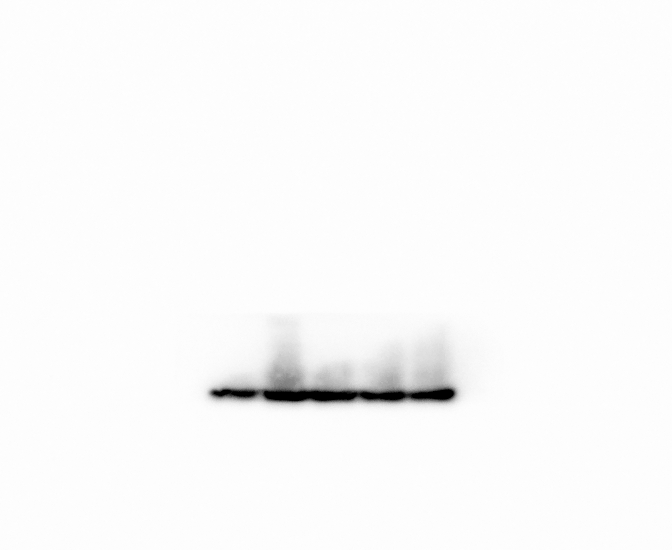

Supplement: Supplementary file 6 [file DataSheet2.ZIP › western blot/figure 4-A/P38_1.tif]

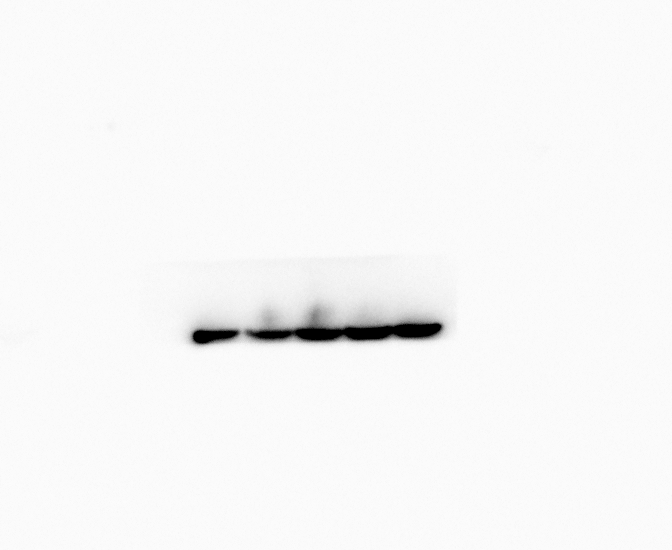

Supplement: Supplementary file 6 [file DataSheet2.ZIP › western blot/figure 4-A/P38_2.tif]

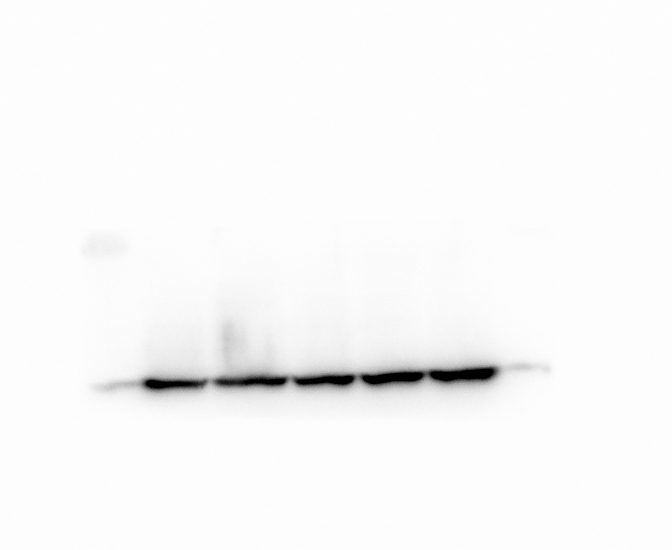

Supplement: Supplementary file 6 [file DataSheet2.ZIP › western blot/figure 4-A/P38_3.tif]

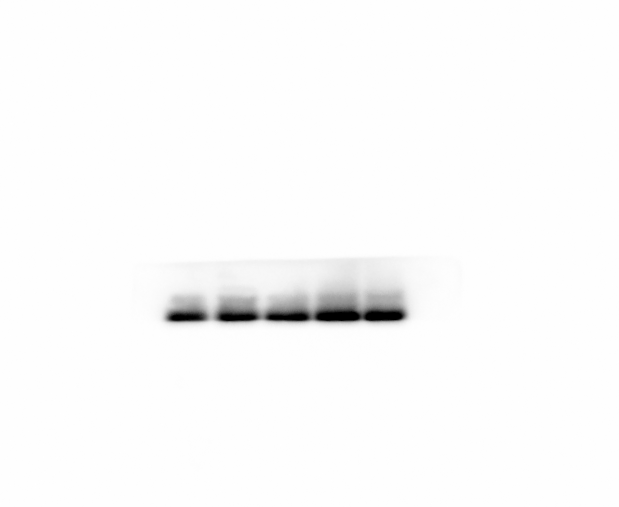

Supplement: Supplementary file 6 [file DataSheet2.ZIP › western blot/figure 4-A/P65-1.tif]

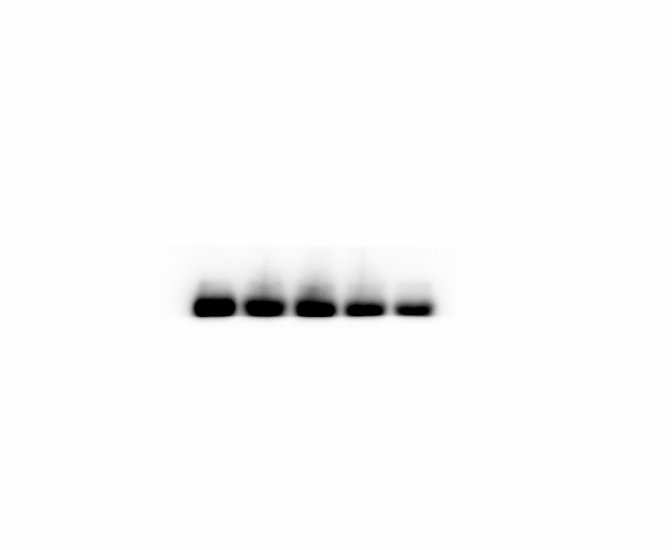

Supplement: Supplementary file 6 [file DataSheet2.ZIP › western blot/figure 4-A/P65-2.tif]

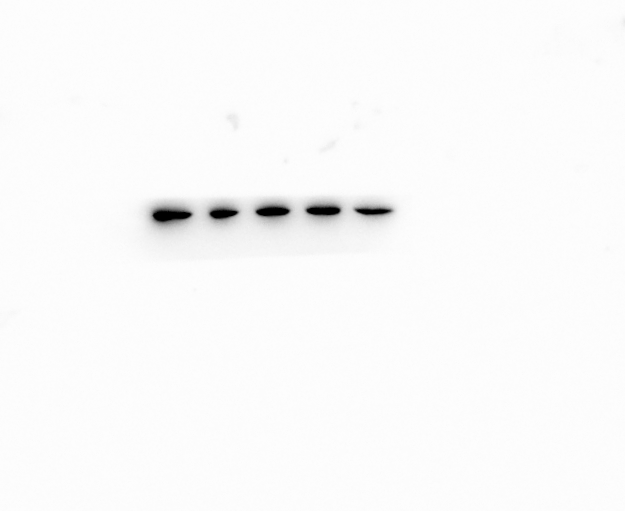

Supplement: Supplementary file 6 [file DataSheet2.ZIP › western blot/figure 4-A/P65-3.tif]

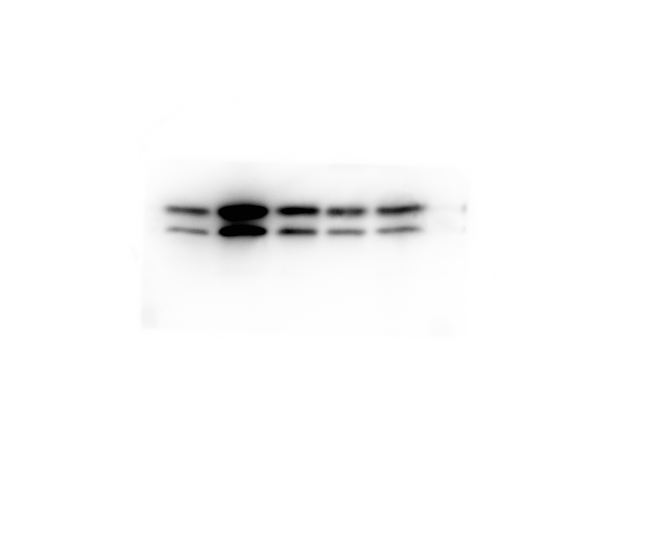

Supplement: Supplementary file 6 [file DataSheet2.ZIP › western blot/figure 4-A/PERK_1.tif]

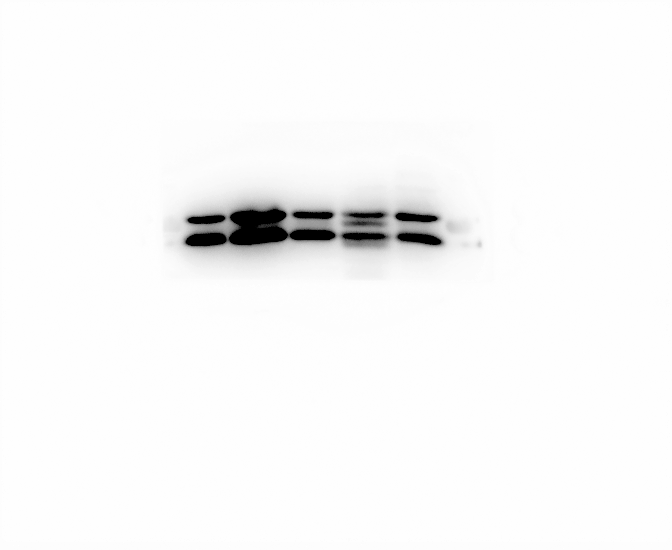

Supplement: Supplementary file 6 [file DataSheet2.ZIP › western blot/figure 4-A/PERK_2.tif]

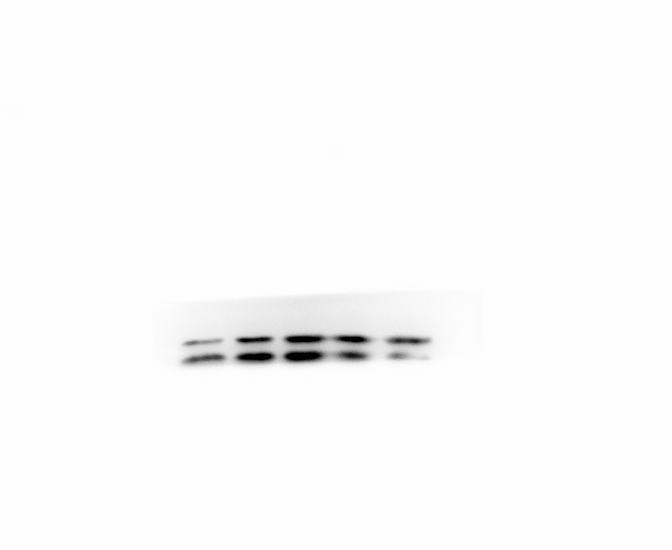

Supplement: Supplementary file 6 [file DataSheet2.ZIP › western blot/figure 4-A/PERK_3.tif]

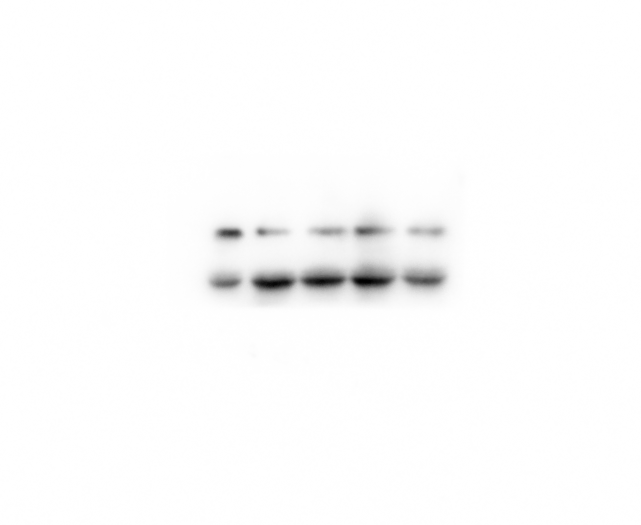

Supplement: Supplementary file 6 [file DataSheet2.ZIP › western blot/figure 4-A/PJNK_1.tif]

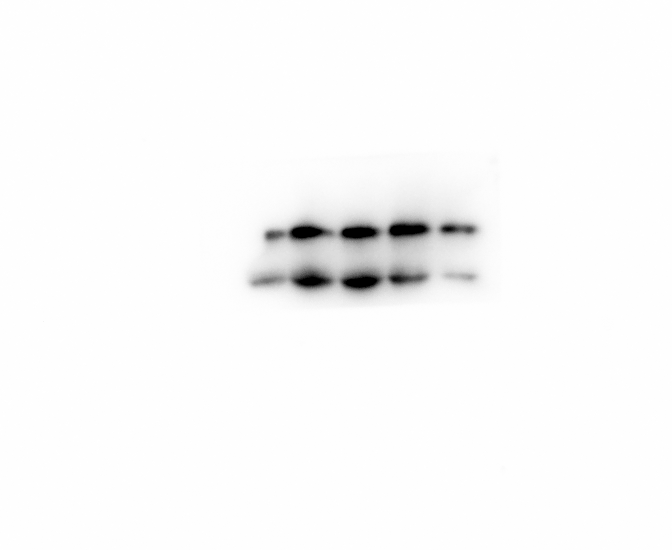

Supplement: Supplementary file 6 [file DataSheet2.ZIP › western blot/figure 4-A/PJNK_2.tif]

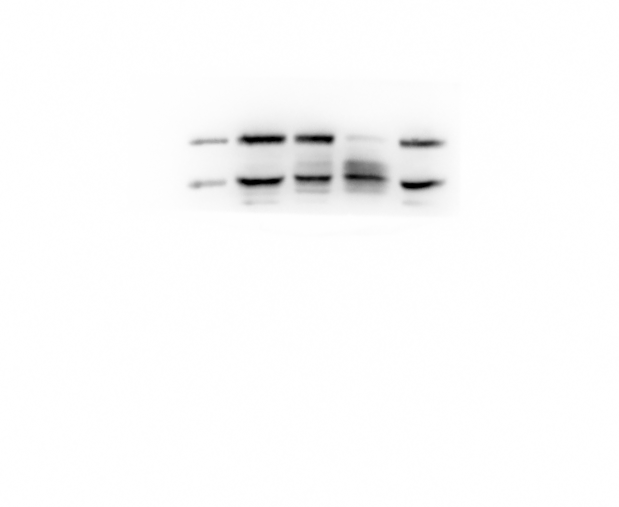

Supplement: Supplementary file 6 [file DataSheet2.ZIP › western blot/figure 4-A/PJNK_3.tif]

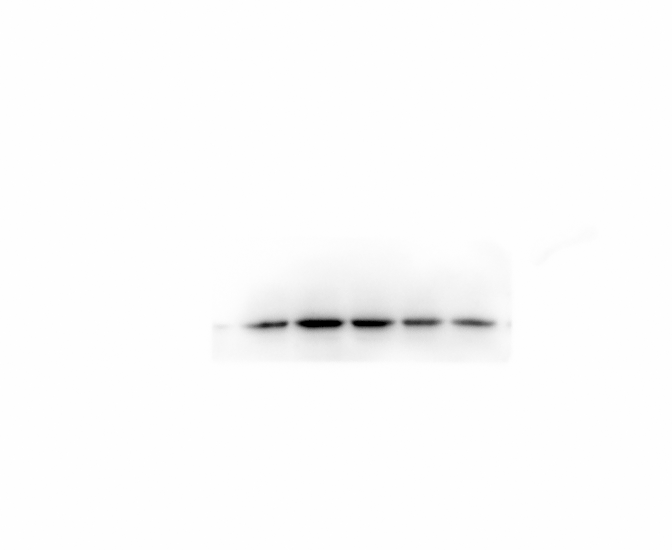

Supplement: Supplementary file 6 [file DataSheet2.ZIP › western blot/figure 4-A/PP38_1.tif]

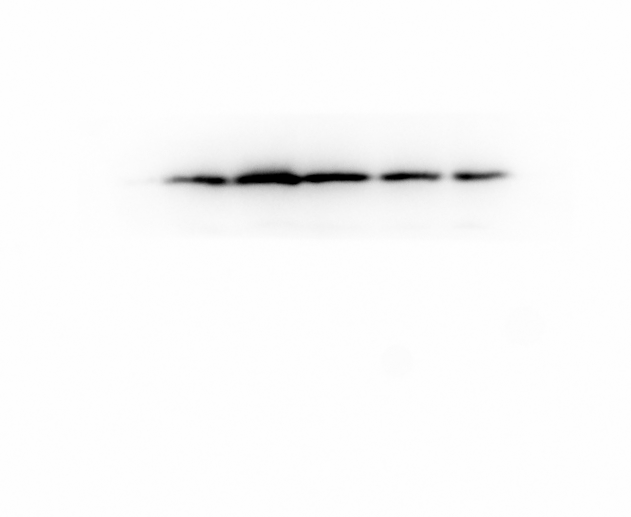

Supplement: Supplementary file 6 [file DataSheet2.ZIP › western blot/figure 4-A/PP38_2.tif]

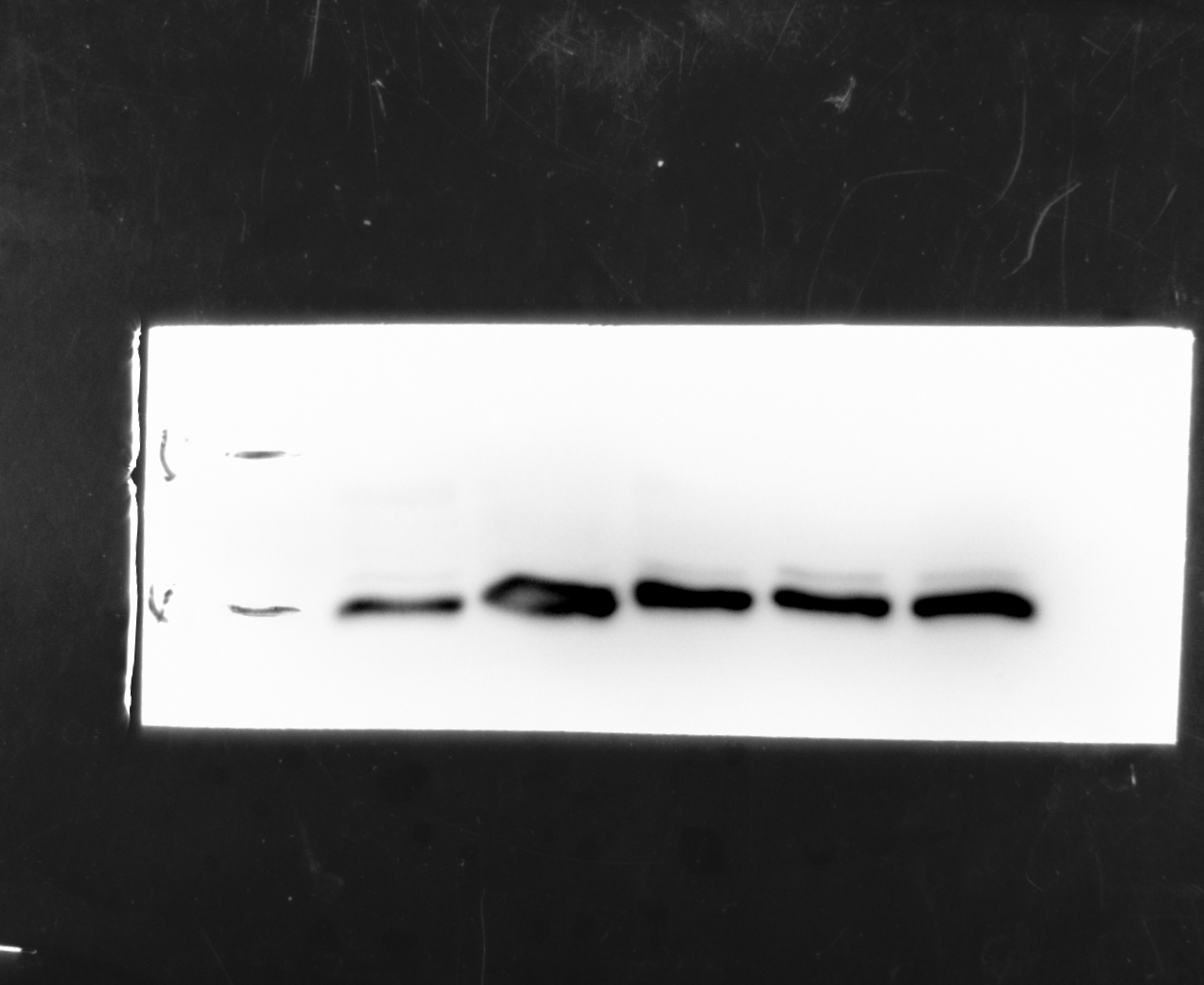

Supplement: Supplementary file 6 [file DataSheet2.ZIP › western blot/figure 4-A/PP38_3.tif]

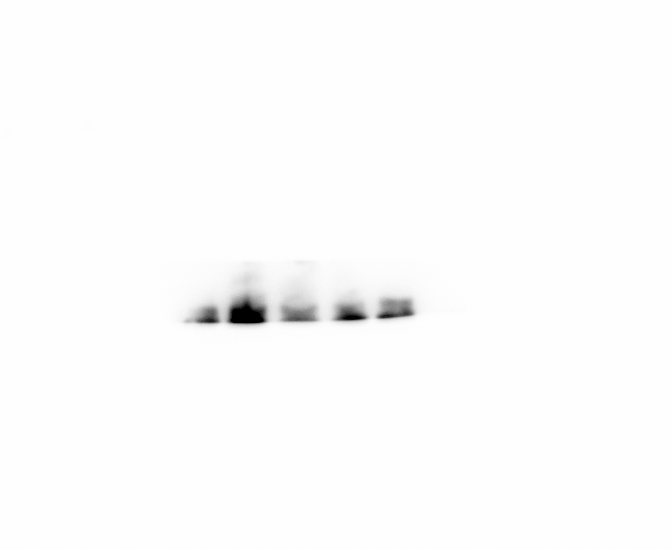

Supplement: Supplementary file 6 [file DataSheet2.ZIP › western blot/figure 4-A/PP65-1.tif]

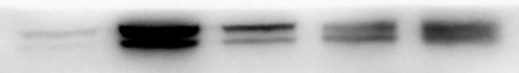

Supplement: Supplementary file 6 [file DataSheet2.ZIP › western blot/figure 4-A/PP65-2.tif]

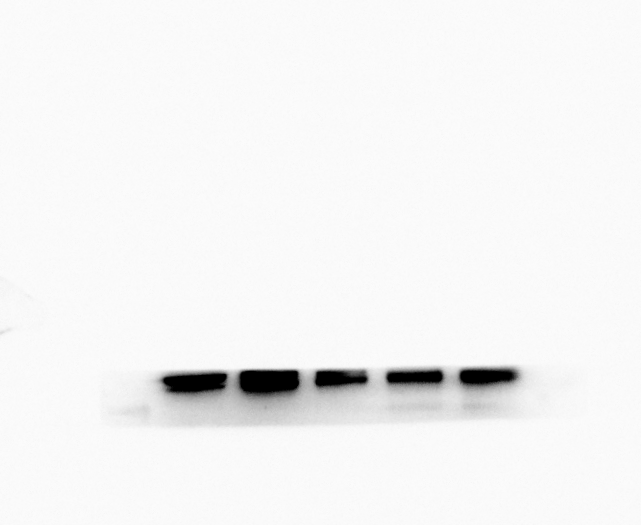

Supplement: Supplementary file 6 [file DataSheet2.ZIP › western blot/figure 4-A/PP65-3.tif]

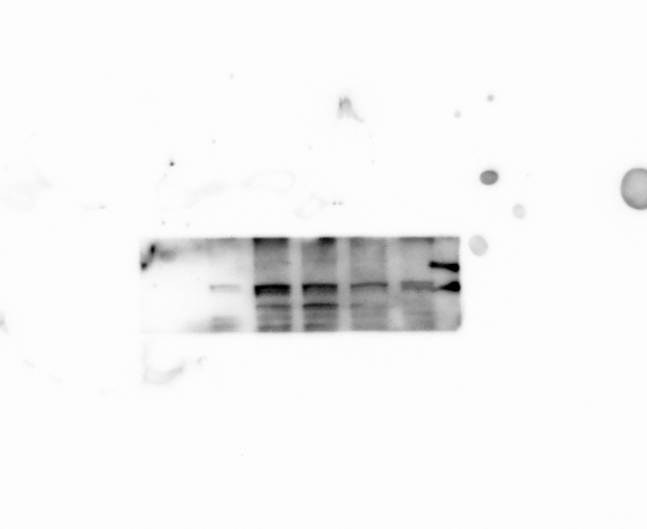

Supplement: Supplementary file 6 [file DataSheet2.ZIP › western blot/figure 5-M/iNOS_1.tif]

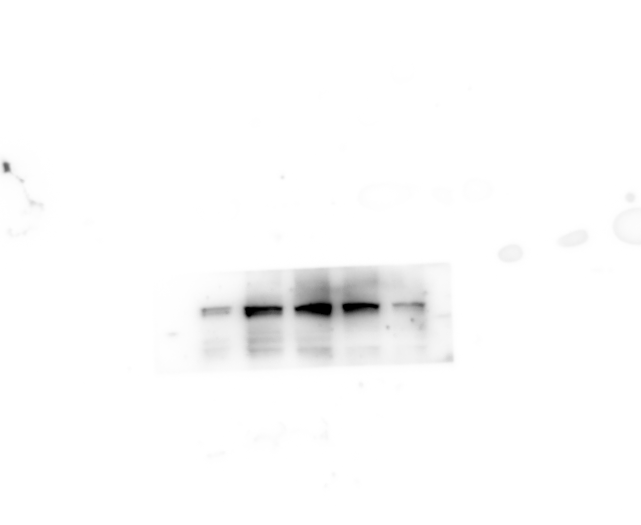

Supplement: Supplementary file 6 [file DataSheet2.ZIP › western blot/figure 5-M/iNOS_2.tif]

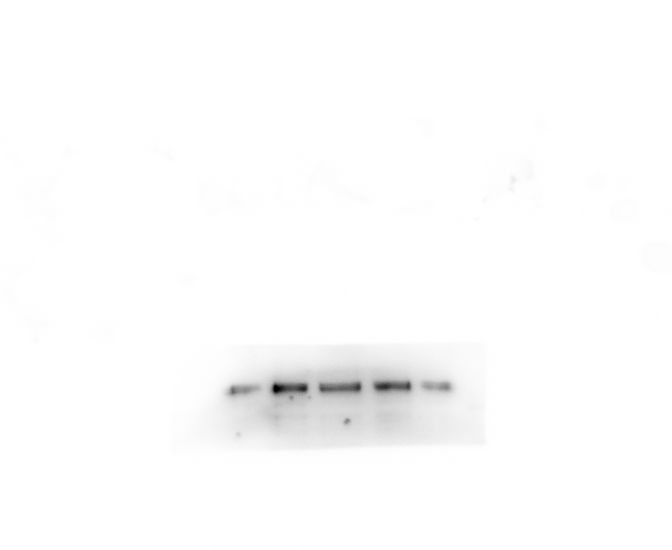

Supplement: Supplementary file 6 [file DataSheet2.ZIP › western blot/figure 5-M/iNOS_3.tif]

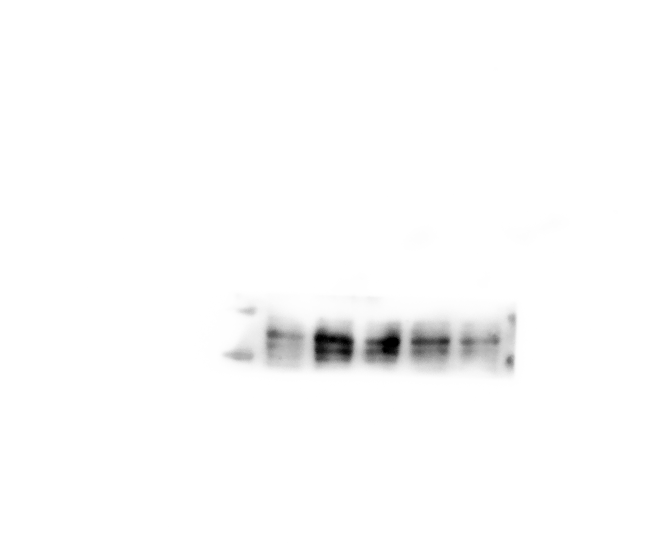

Supplement: Supplementary file 6 [file DataSheet2.ZIP › western blot/figure 5-M/LOX1-1.tif]

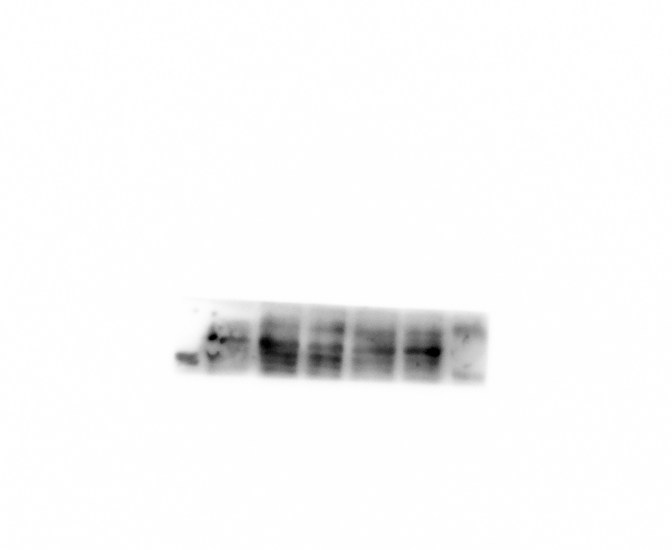

Supplement: Supplementary file 6 [file DataSheet2.ZIP › western blot/figure 5-M/LOX1-2.tif]

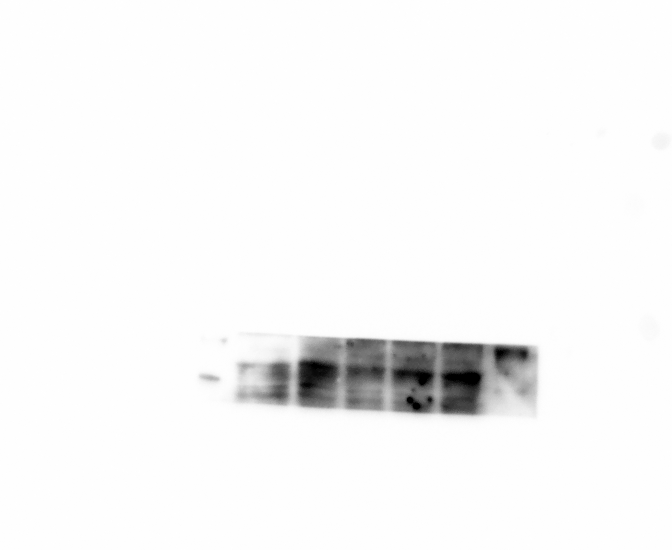

Supplement: Supplementary file 6 [file DataSheet2.ZIP › western blot/figure 5-M/LOX1-3.tif]

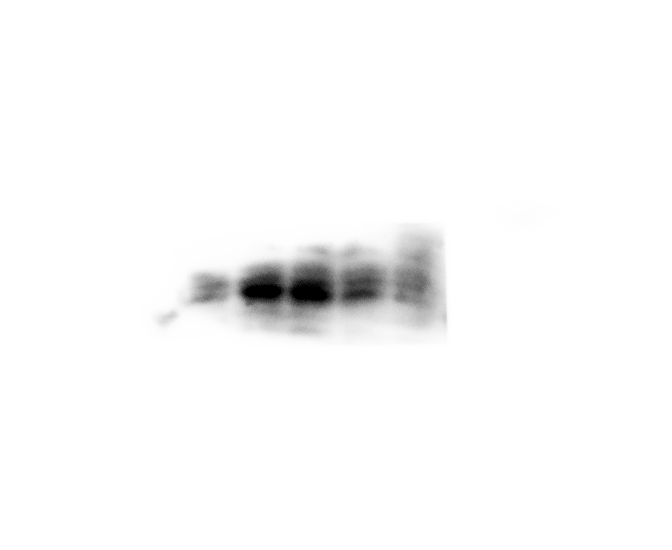

Supplement: Supplementary file 6 [file DataSheet2.ZIP › western blot/figure 5-M/MCP1-1.tif]

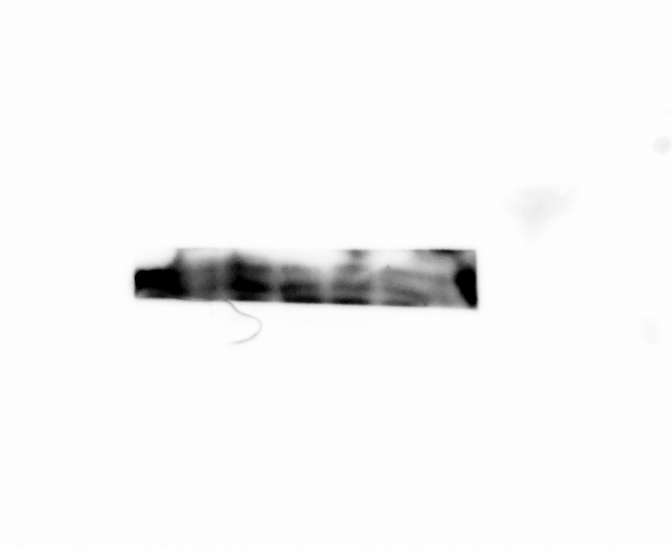

Supplement: Supplementary file 6 [file DataSheet2.ZIP › western blot/figure 5-M/MCP1-2.tif]

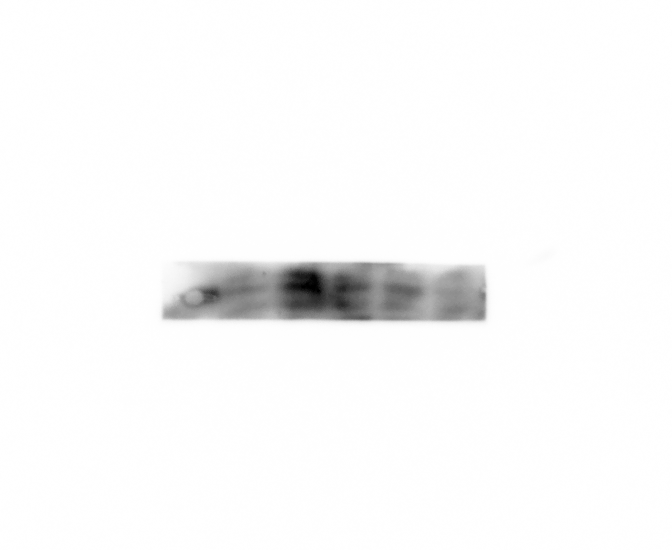

Supplement: Supplementary file 6 [file DataSheet2.ZIP › western blot/figure 5-M/MCP1-3.tif]

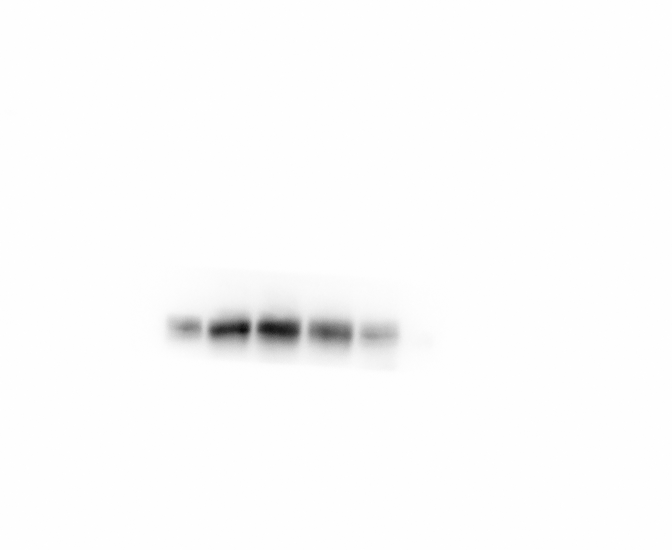

Supplement: Supplementary file 6 [file DataSheet2.ZIP › western blot/figure 5-M/VCAM_1.tif]

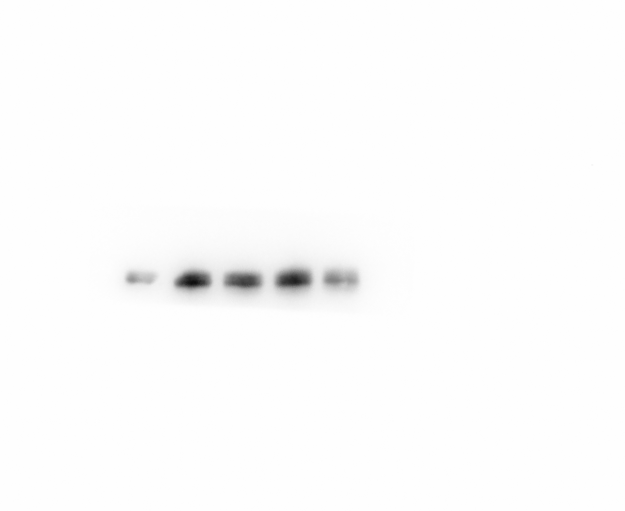

Supplement: Supplementary file 6 [file DataSheet2.ZIP › western blot/figure 5-M/VCAM_2.tif]

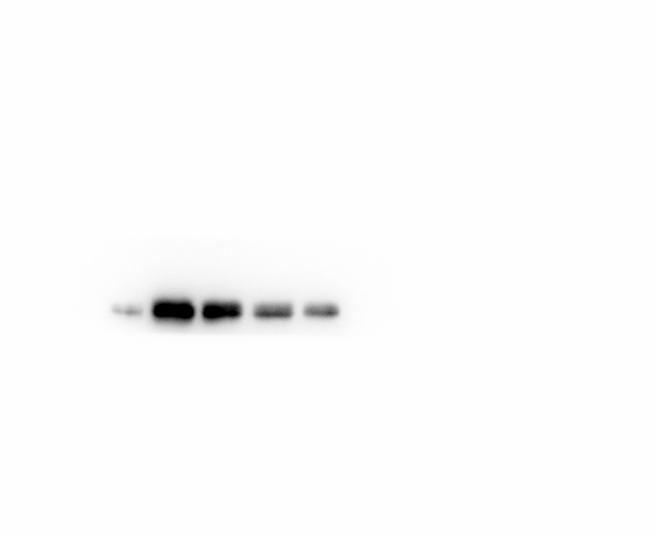

Supplement: Supplementary file 6 [file DataSheet2.ZIP › western blot/figure 5-M/VCAM_3.tif]

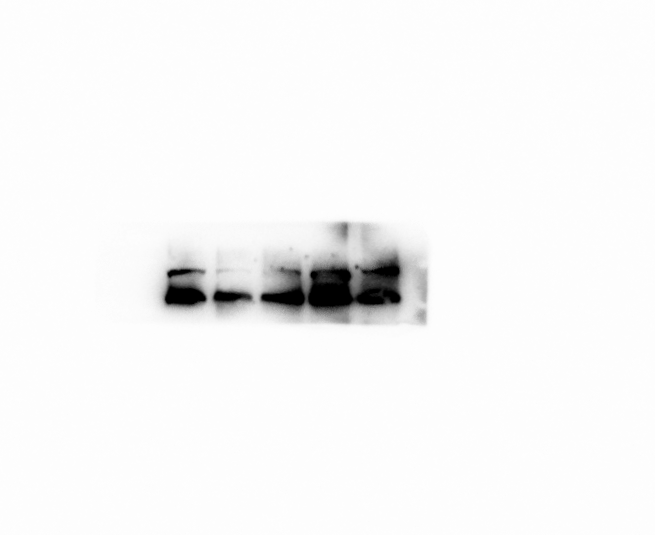

Supplement: Supplementary file 6 [file DataSheet2.ZIP › western blot/figure 6-E/AXL-1.tif]

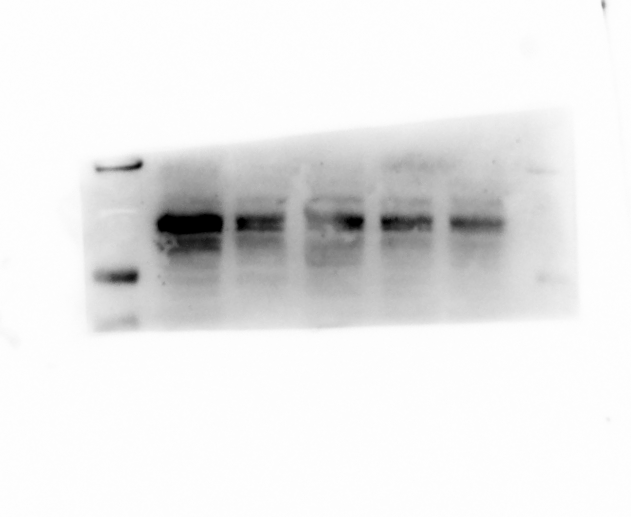

Supplement: Supplementary file 6 [file DataSheet2.ZIP › western blot/figure 6-E/AXL-2.tif]

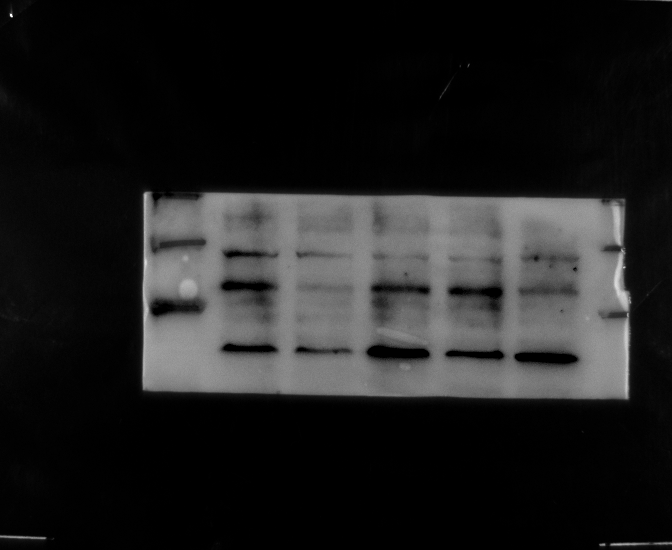

Supplement: Supplementary file 6 [file DataSheet2.ZIP › western blot/figure 6-E/AXL-3_.tif]

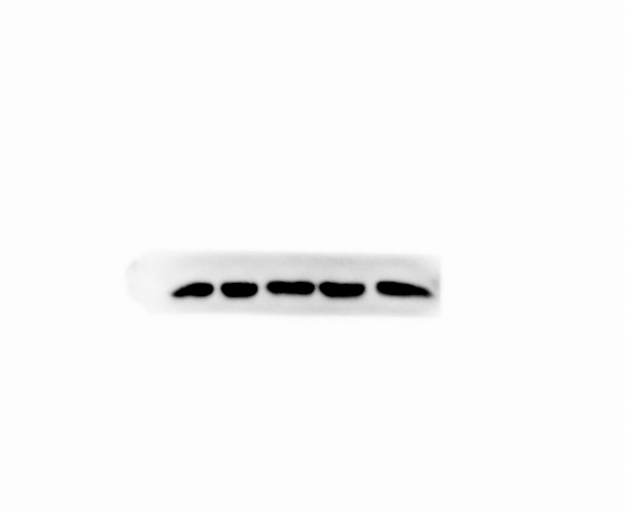

Supplement: Supplementary file 6 [file DataSheet2.ZIP › western blot/figure 6-E/GAPDH-1.tif]

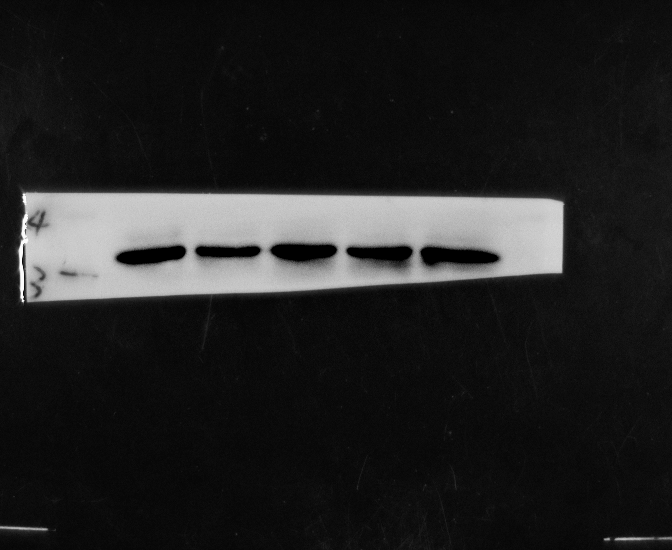

Supplement: Supplementary file 6 [file DataSheet2.ZIP › western blot/figure 6-E/GAPDH-2.tif]

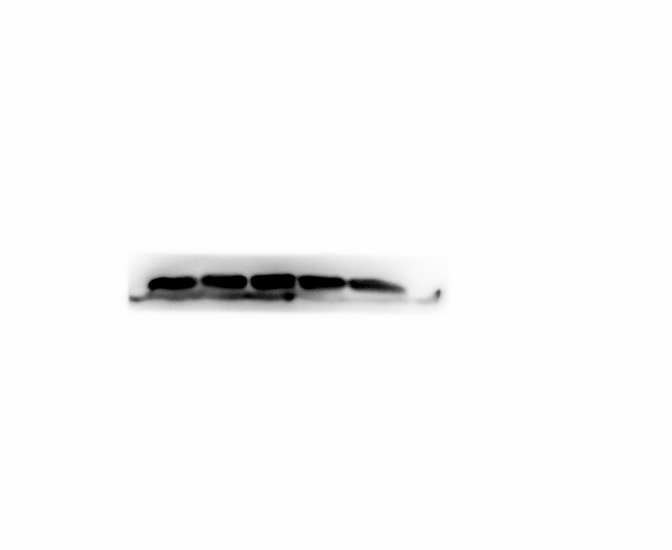

Supplement: Supplementary file 6 [file DataSheet2.ZIP › western blot/figure 6-E/GAPDH-3.tif]

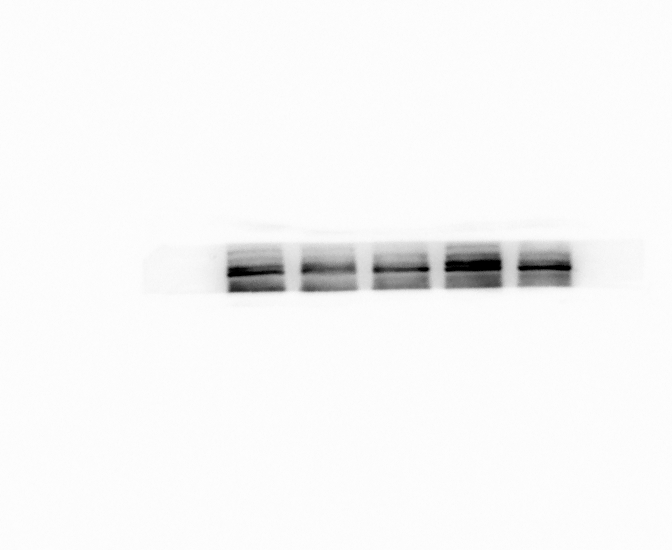

Supplement: Supplementary file 6 [file DataSheet2.ZIP › western blot/figure 6-E/MERTK-1.tif]

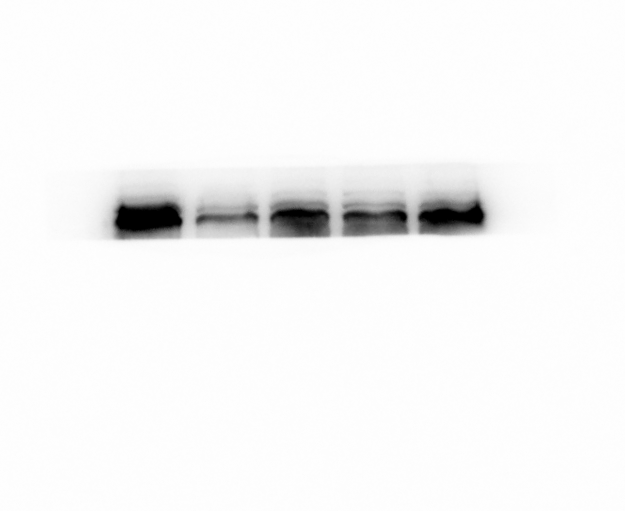

Supplement: Supplementary file 6 [file DataSheet2.ZIP › western blot/figure 6-E/MERTK-2.tif]

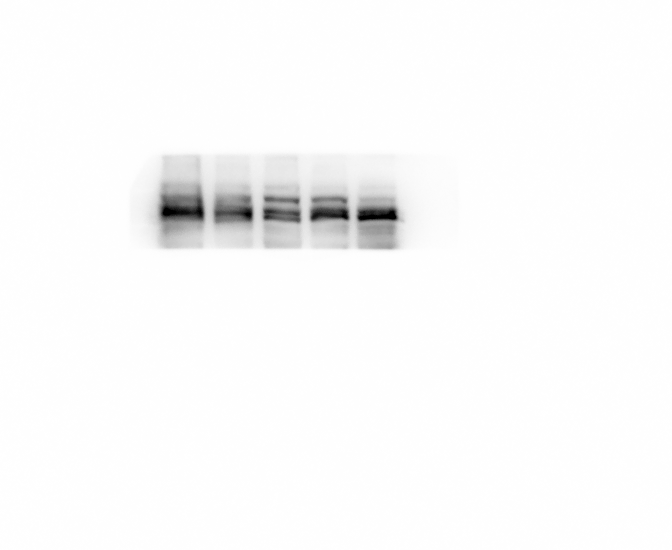

Supplement: Supplementary file 6 [file DataSheet2.ZIP › western blot/figure 6-E/MERTK-3.tif]

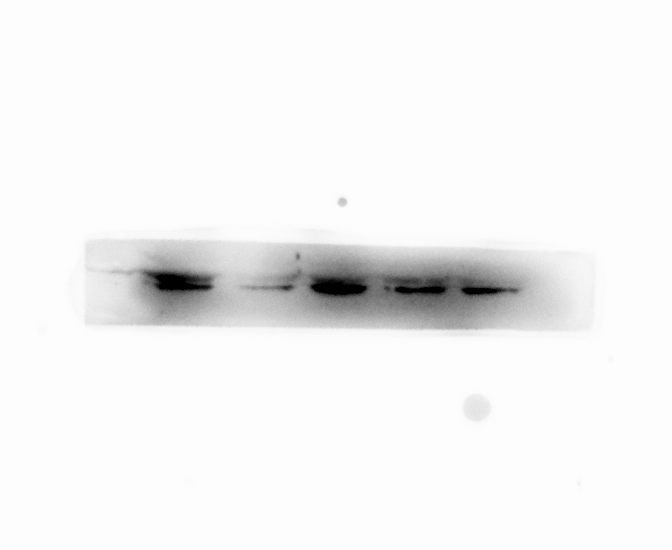

Supplement: Supplementary file 6 [file DataSheet2.ZIP › western blot/figure 6-E/MFGE8-1.tif]

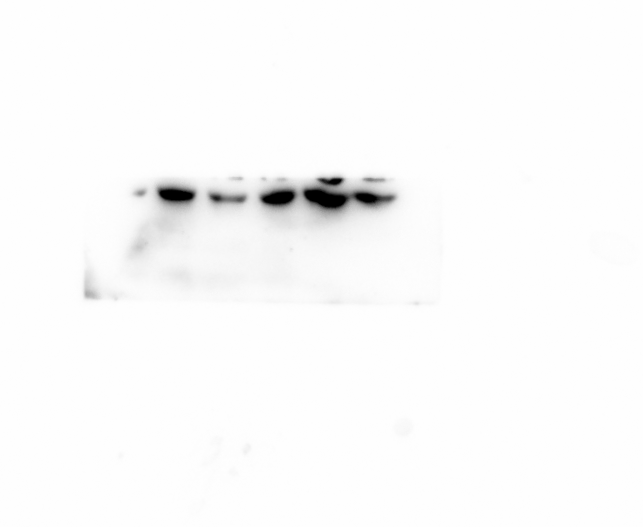

Supplement: Supplementary file 6 [file DataSheet2.ZIP › western blot/figure 6-E/MFGE8-2.tif]

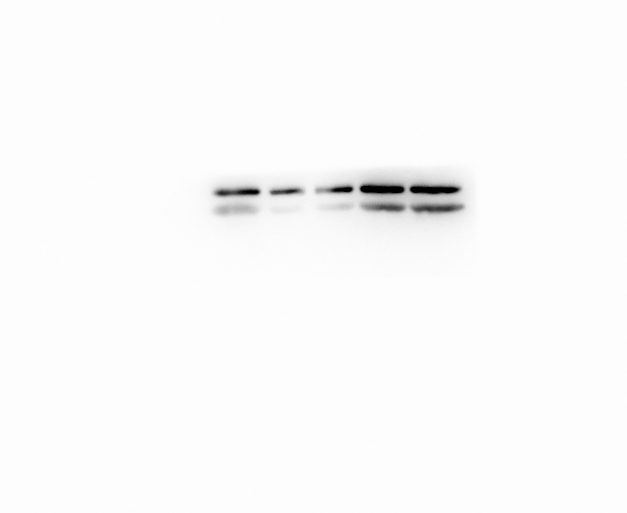

Supplement: Supplementary file 6 [file DataSheet2.ZIP › western blot/figure 6-E/MFGE8-3.tif]

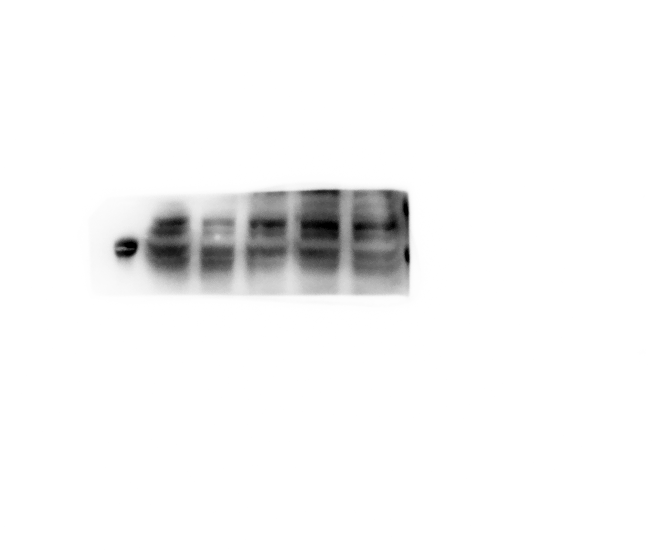

Supplement: Supplementary file 6 [file DataSheet2.ZIP › western blot/figure 6-E/TYRO-1.tif]

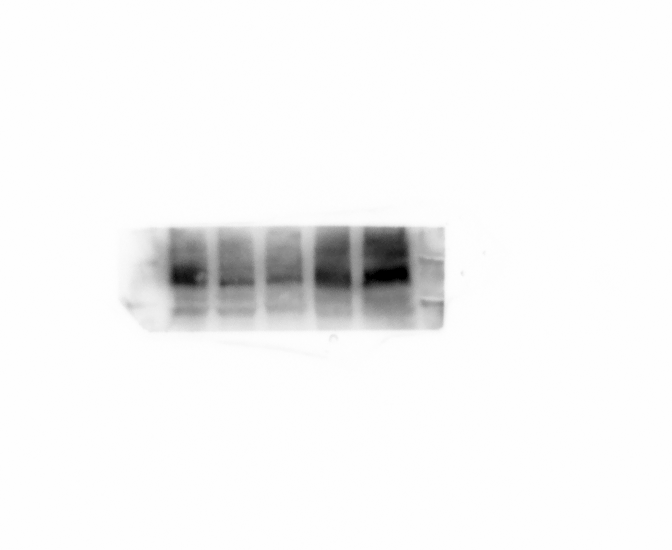

Supplement: Supplementary file 6 [file DataSheet2.ZIP › western blot/figure 6-E/TYRO-2.tif]

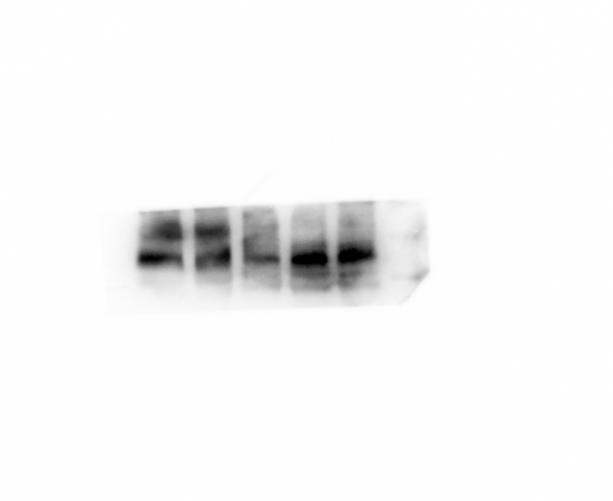

Supplement: Supplementary file 6 [file DataSheet2.ZIP › western blot/figure 6-E/TYRO-3.tif]

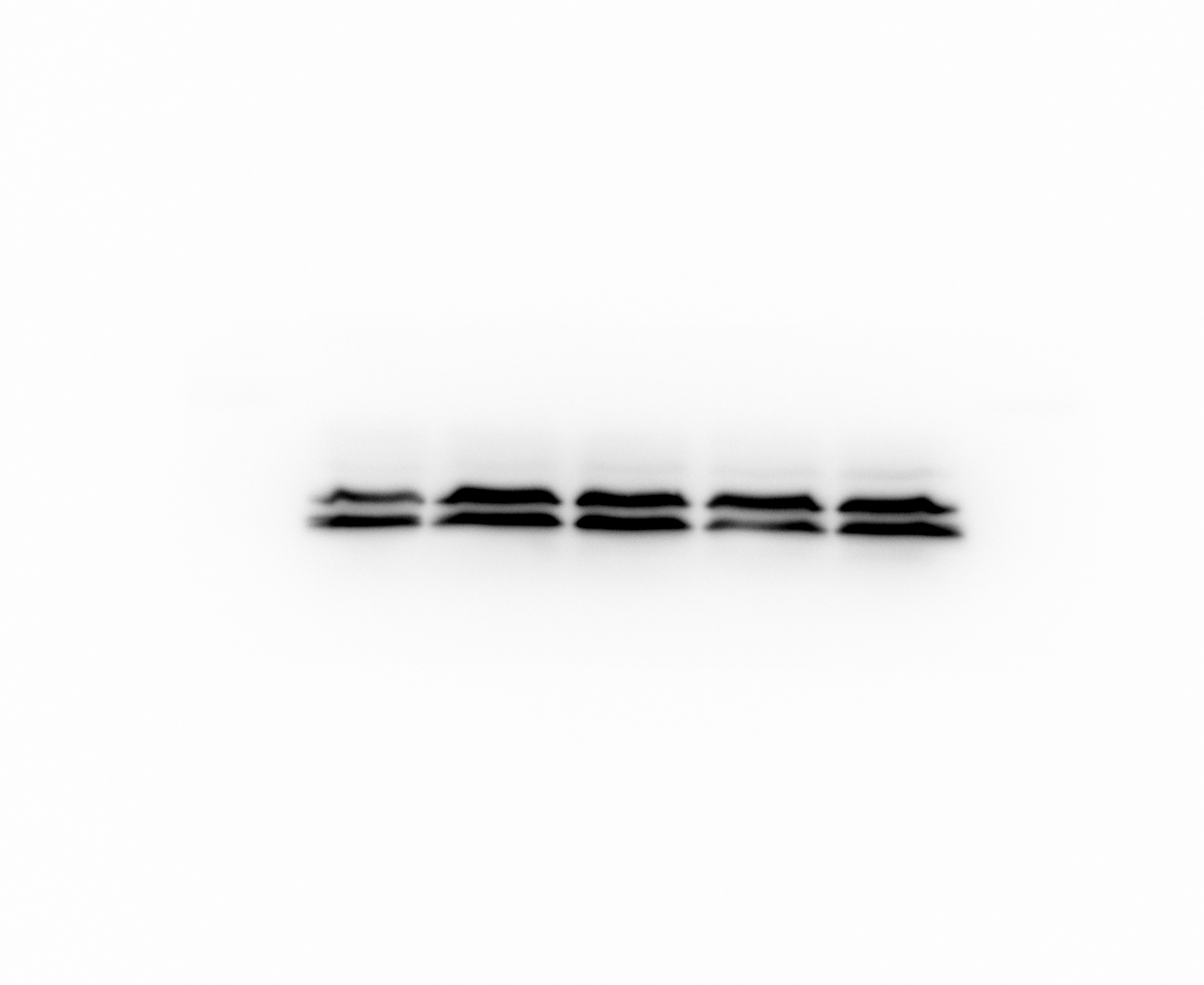

Supplement: Supplementary file 6 [file DataSheet2.ZIP › western blot/figure 7-A/ERK-1.tif]

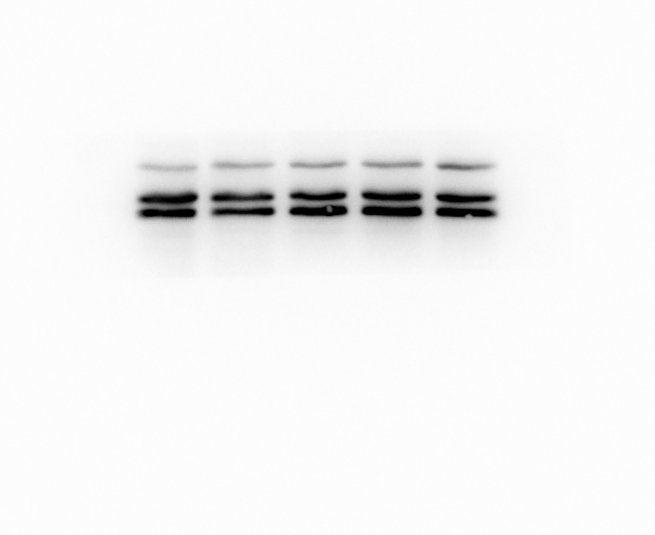

Supplement: Supplementary file 6 [file DataSheet2.ZIP › western blot/figure 7-A/ERK-2.tif]

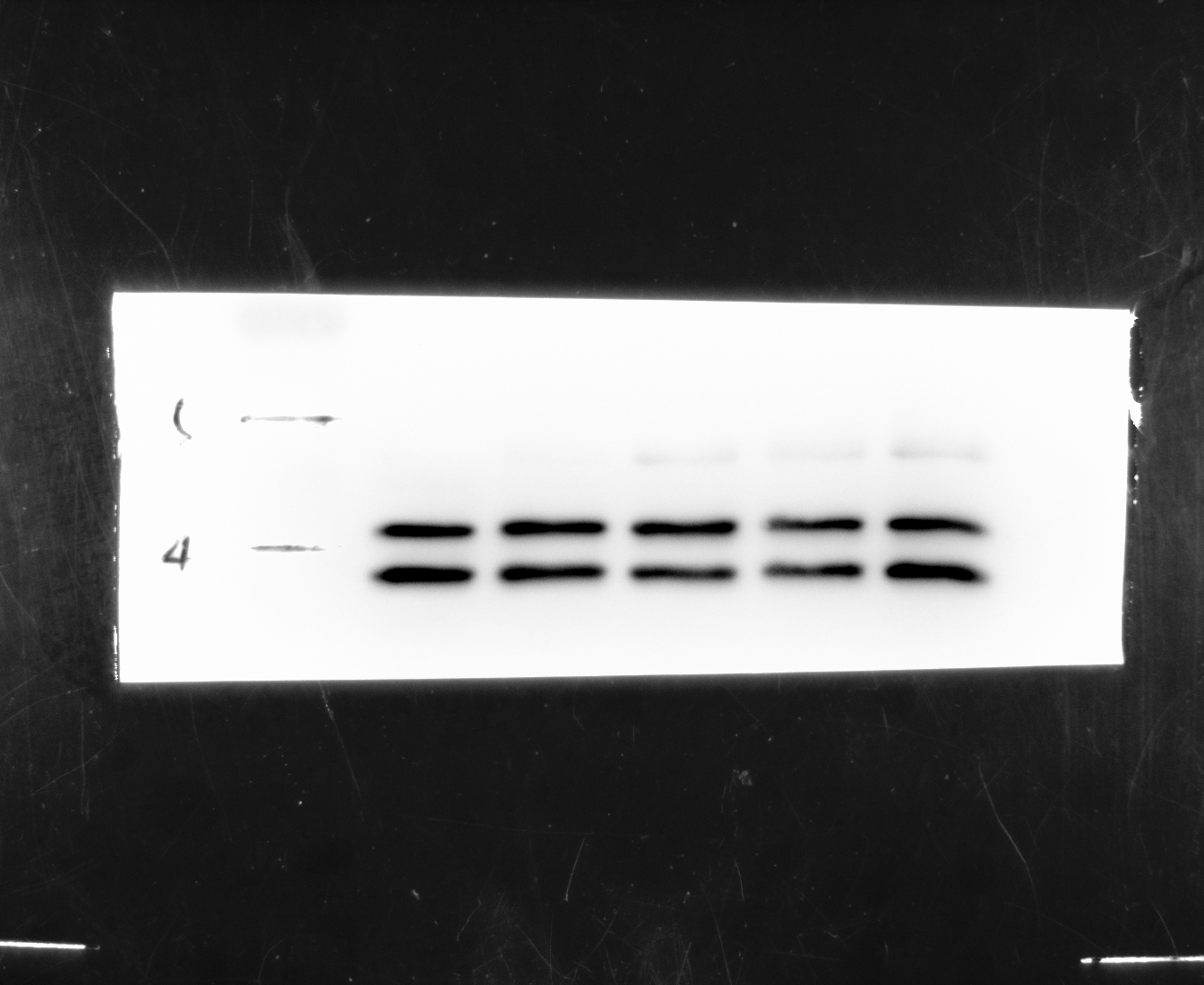

Supplement: Supplementary file 6 [file DataSheet2.ZIP › western blot/figure 7-A/ERK-3.tif]

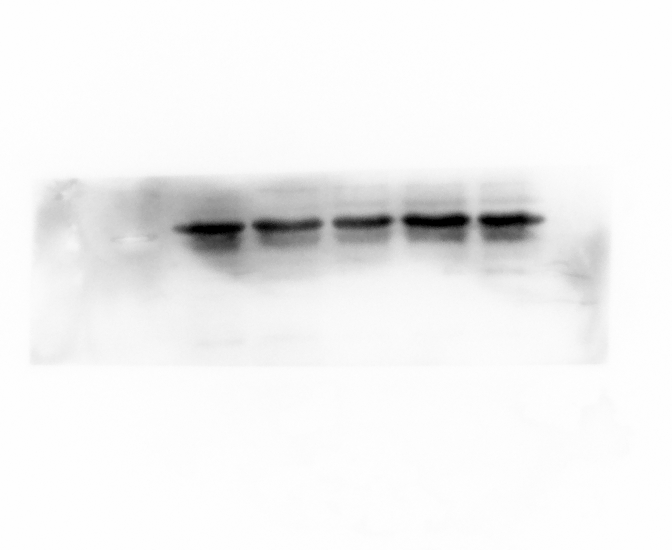

Supplement: Supplementary file 6 [file DataSheet2.ZIP › western blot/figure 7-A/GAPDH-1.tif]

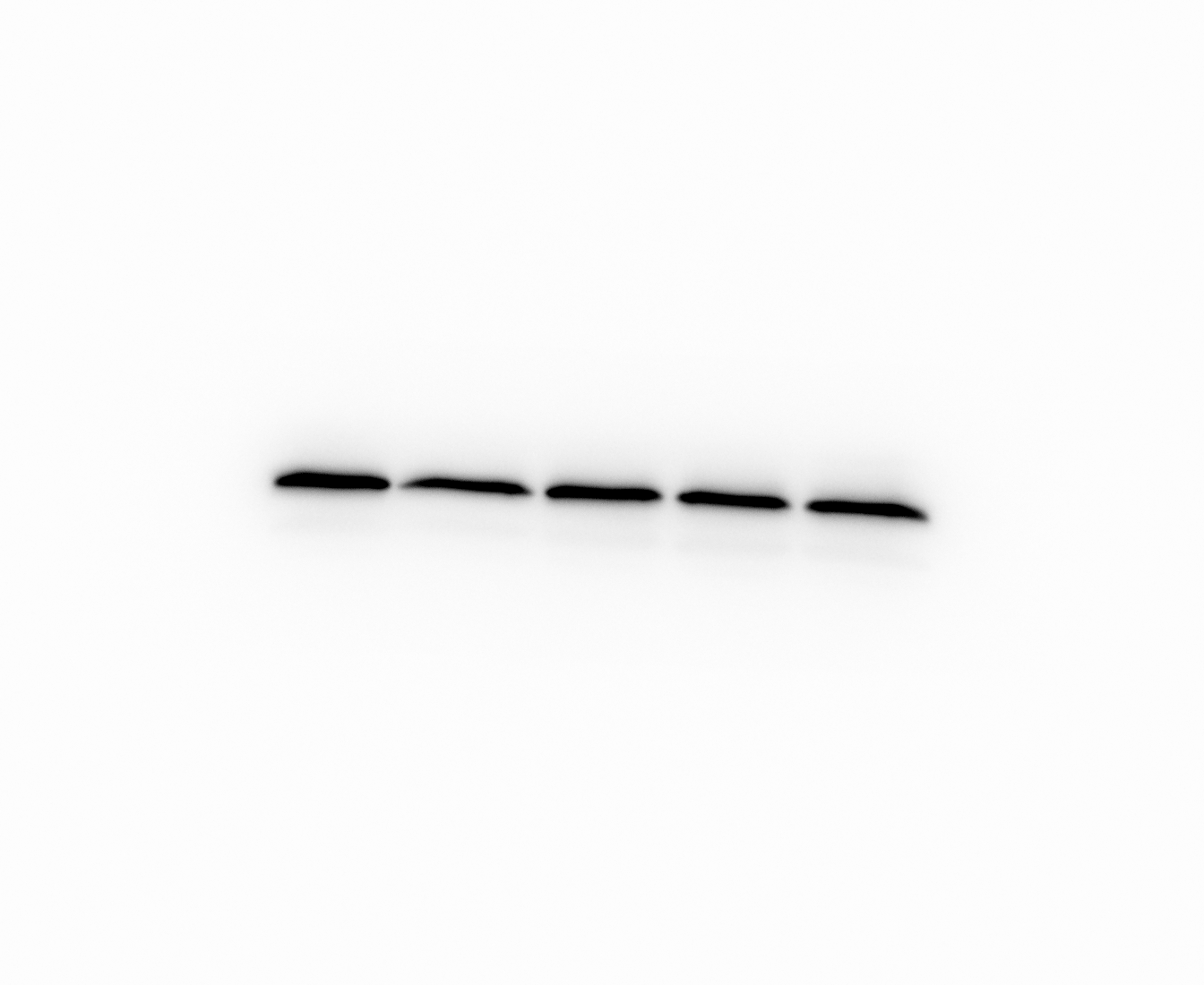

Supplement: Supplementary file 6 [file DataSheet2.ZIP › western blot/figure 7-A/GAPDH-2.tif]

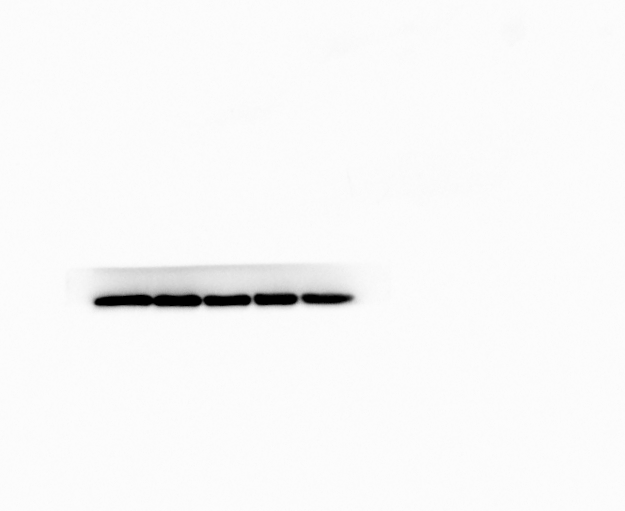

Supplement: Supplementary file 6 [file DataSheet2.ZIP › western blot/figure 7-A/GAPDH-3.tif]

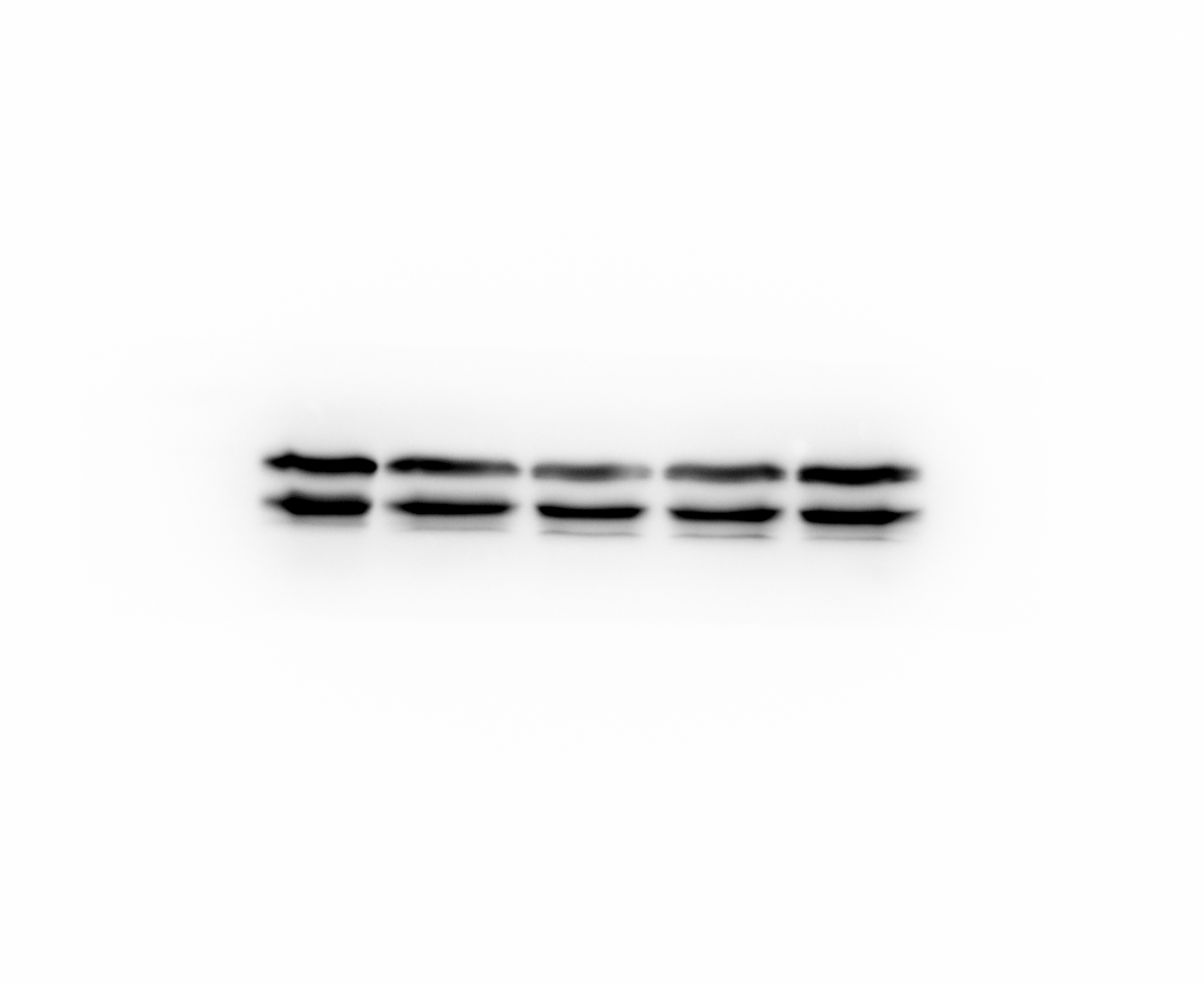

Supplement: Supplementary file 6 [file DataSheet2.ZIP › western blot/figure 7-A/JNK_1.tif]

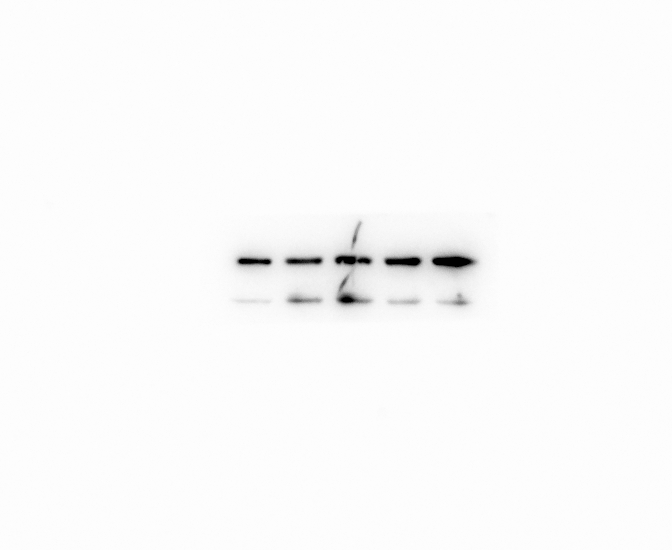

Supplement: Supplementary file 6 [file DataSheet2.ZIP › western blot/figure 7-A/JNK_2.tif]

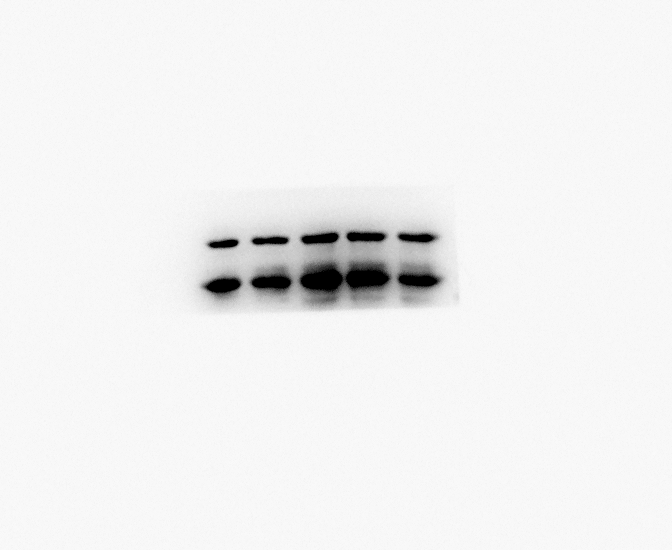

Supplement: Supplementary file 6 [file DataSheet2.ZIP › western blot/figure 7-A/JNK_3.tif]

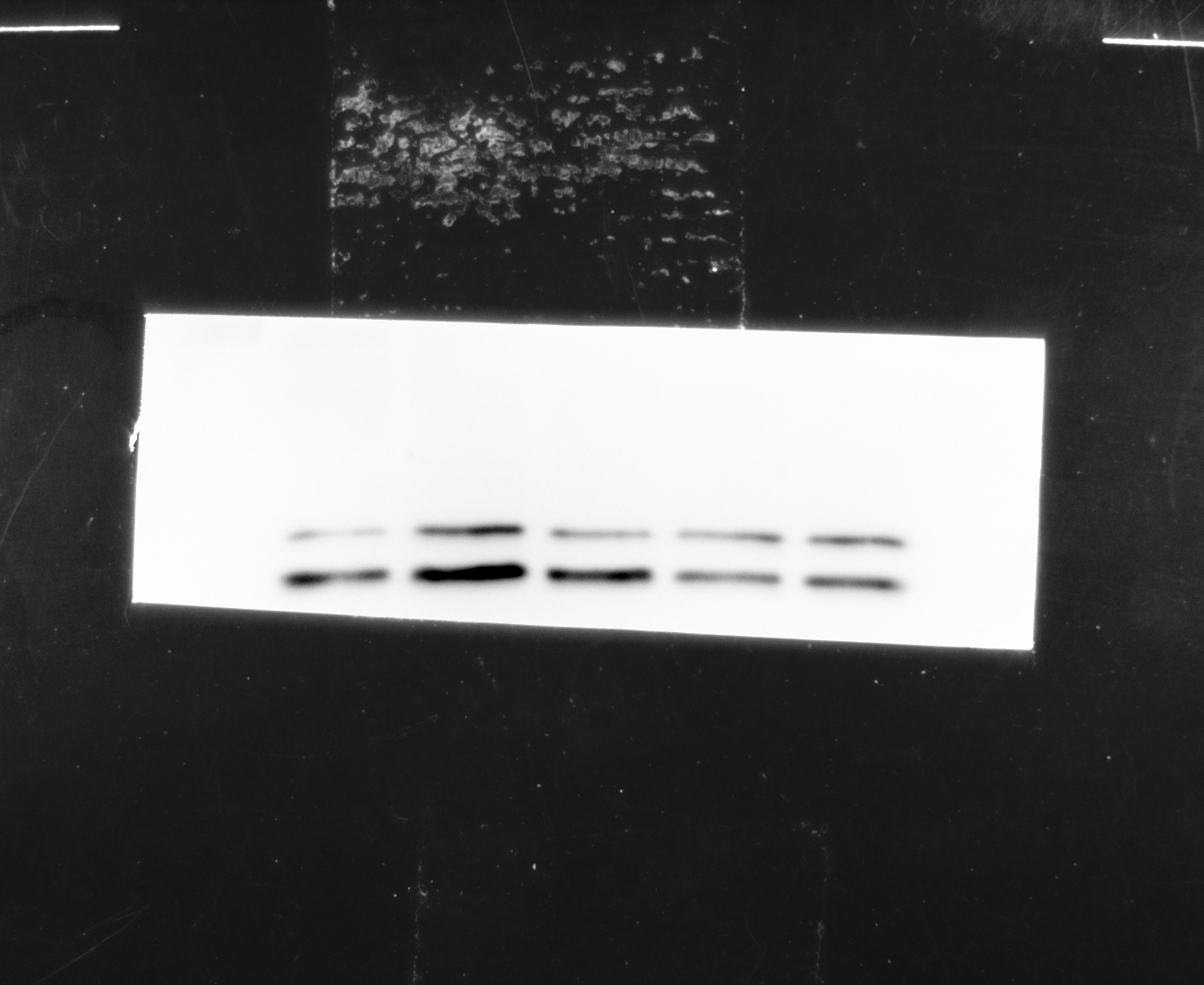

Supplement: Supplementary file 6 [file DataSheet2.ZIP › western blot/figure 7-A/P-ERK-1.tif]

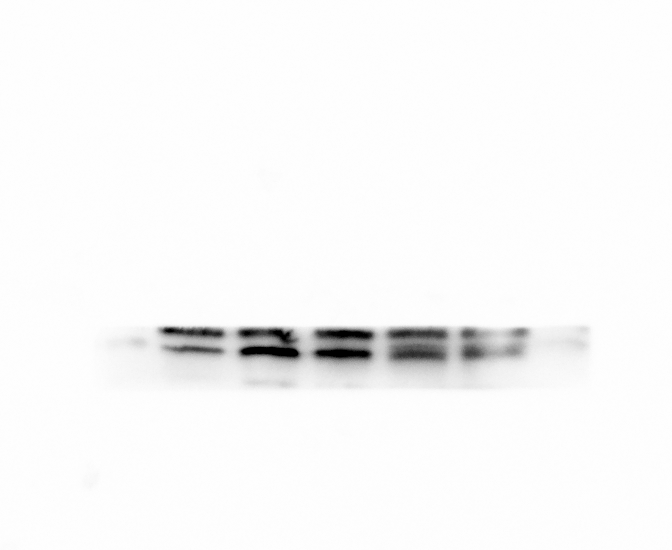

Supplement: Supplementary file 6 [file DataSheet2.ZIP › western blot/figure 7-A/P-ERK-2.tif]

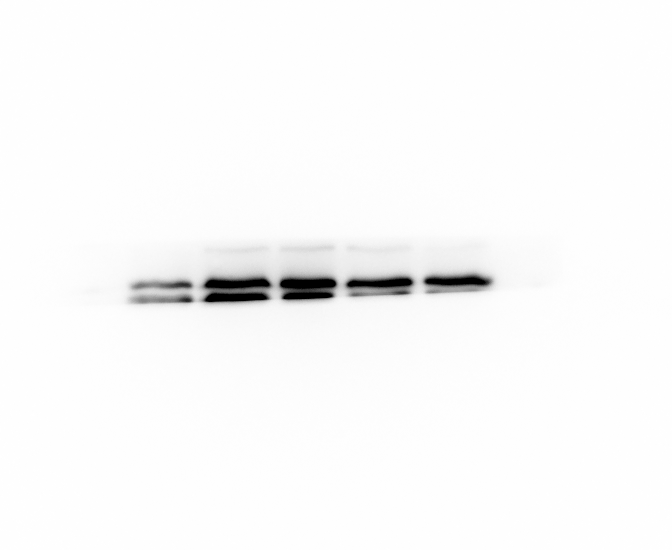

Supplement: Supplementary file 6 [file DataSheet2.ZIP › western blot/figure 7-A/P-ERK-3.tif]

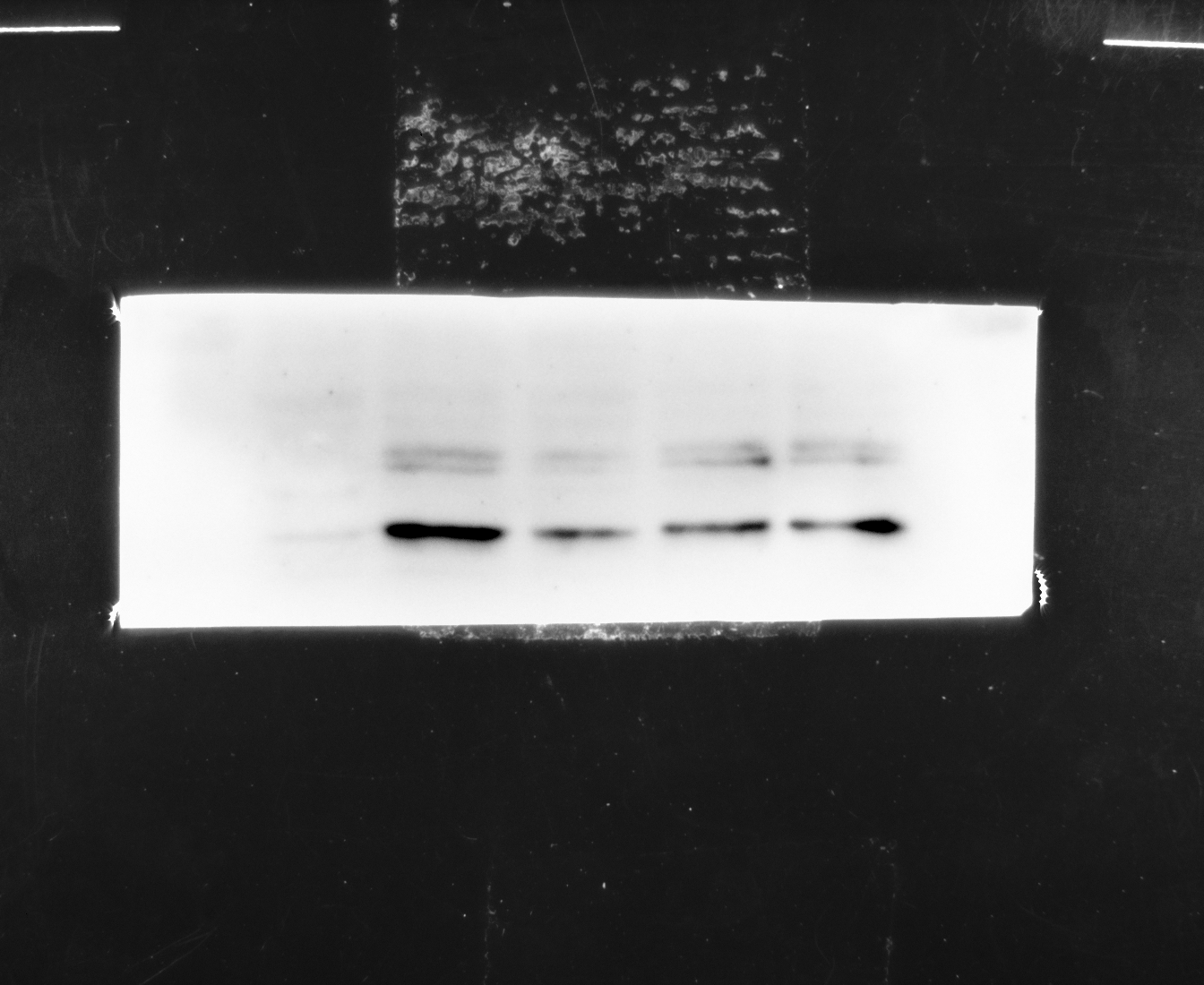

Supplement: Supplementary file 6 [file DataSheet2.ZIP › western blot/figure 7-A/P-JNK-1.tif]

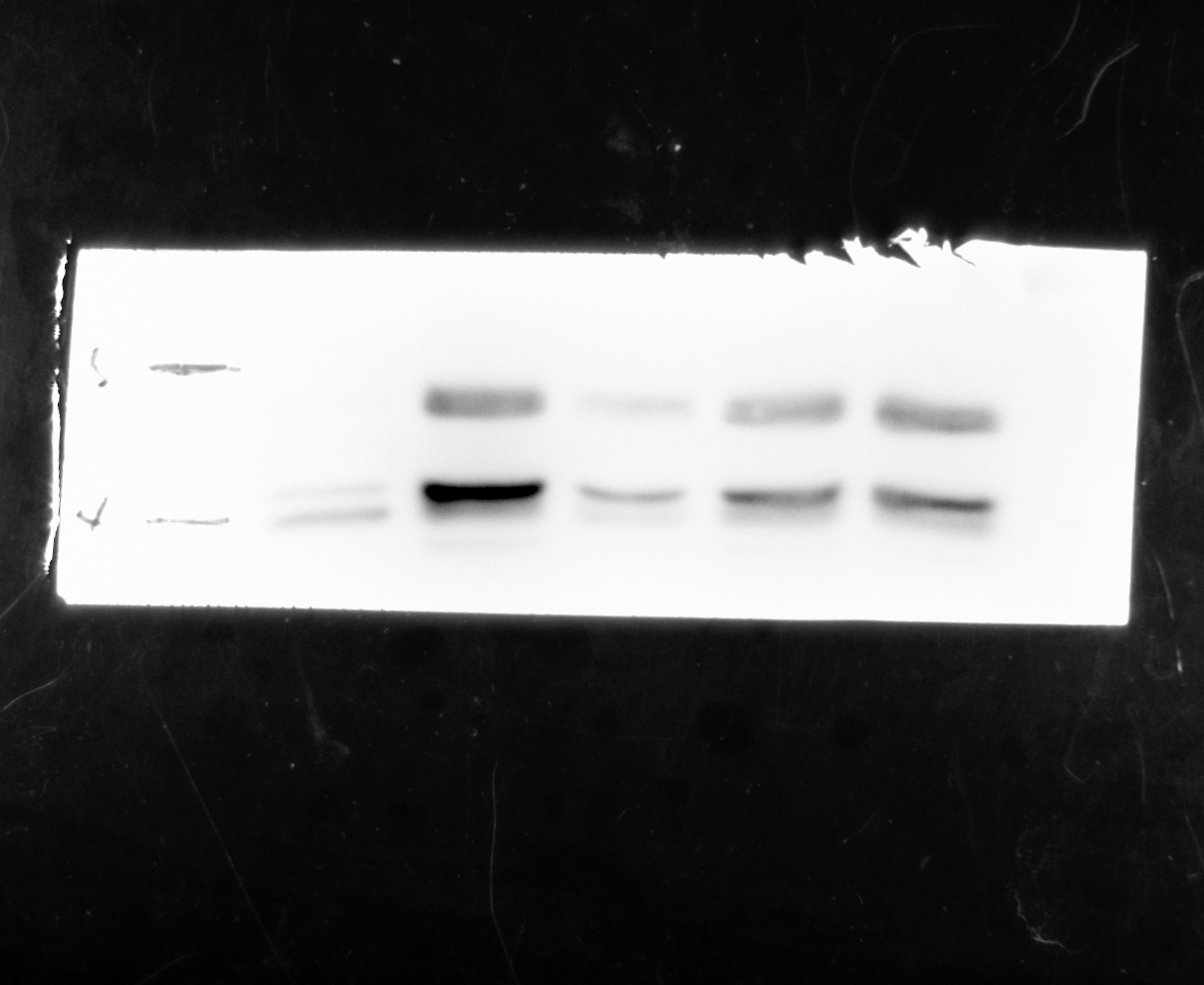

Supplement: Supplementary file 6 [file DataSheet2.ZIP › western blot/figure 7-A/P-JNK-2.tif]

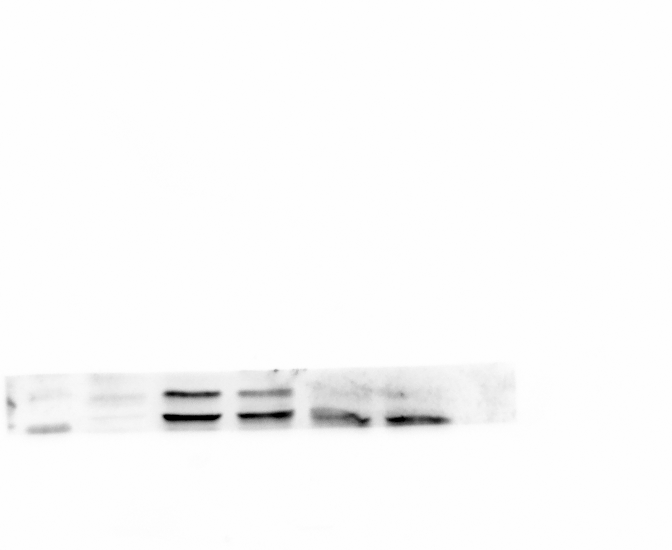

Supplement: Supplementary file 6 [file DataSheet2.ZIP › western blot/figure 7-A/P-JNK-3.tif]

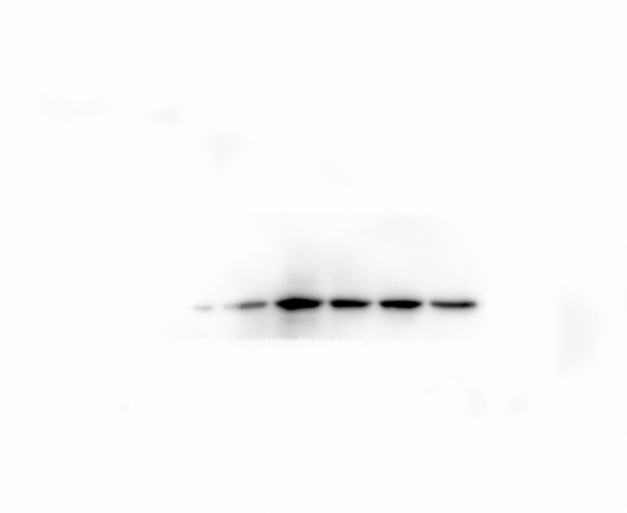

Supplement: Supplementary file 6 [file DataSheet2.ZIP › western blot/figure 7-A/P-P38-1.tif]

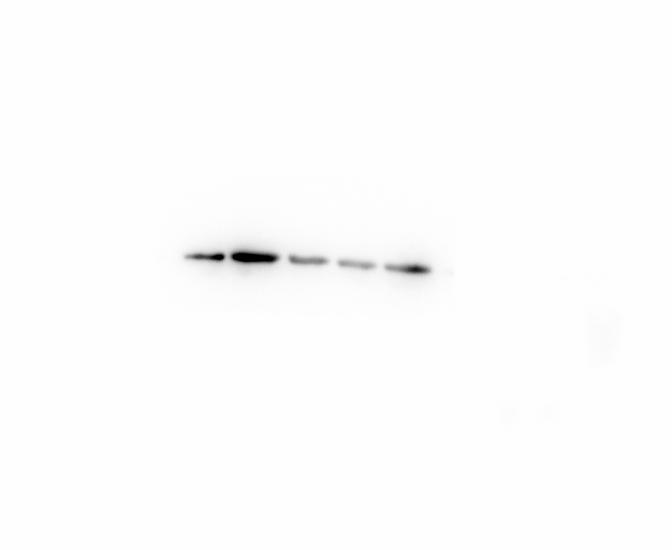

Supplement: Supplementary file 6 [file DataSheet2.ZIP › western blot/figure 7-A/P-P38-2.tif]

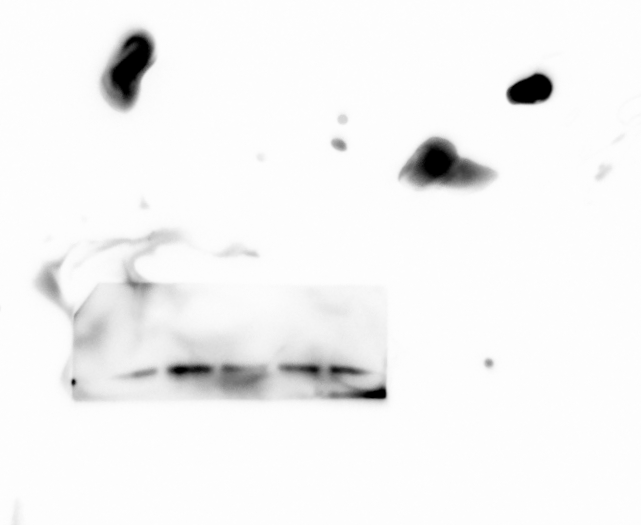

Supplement: Supplementary file 6 [file DataSheet2.ZIP › western blot/figure 7-A/P-P38-3.tif]

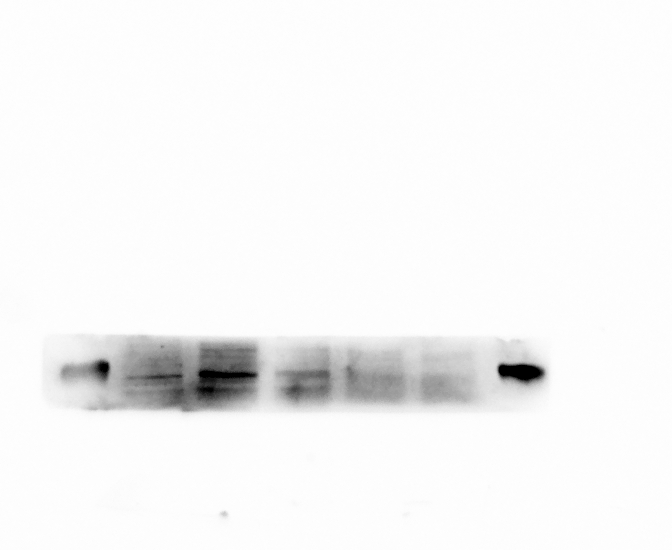

Supplement: Supplementary file 6 [file DataSheet2.ZIP › western blot/figure 7-A/P-P65-1.tif]

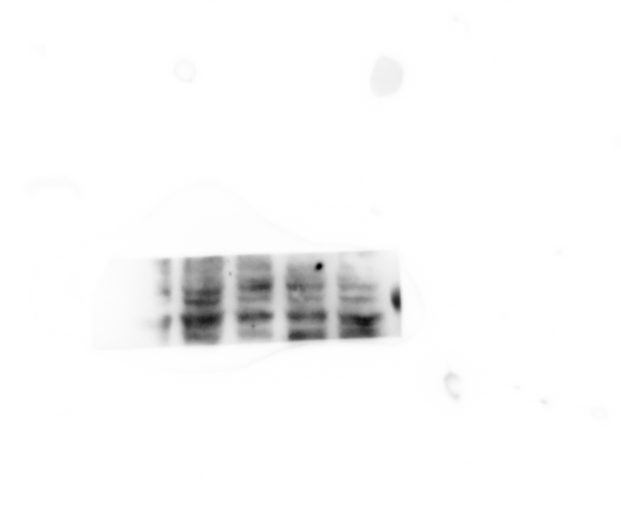

Supplement: Supplementary file 6 [file DataSheet2.ZIP › western blot/figure 7-A/P-P65-2.tif]

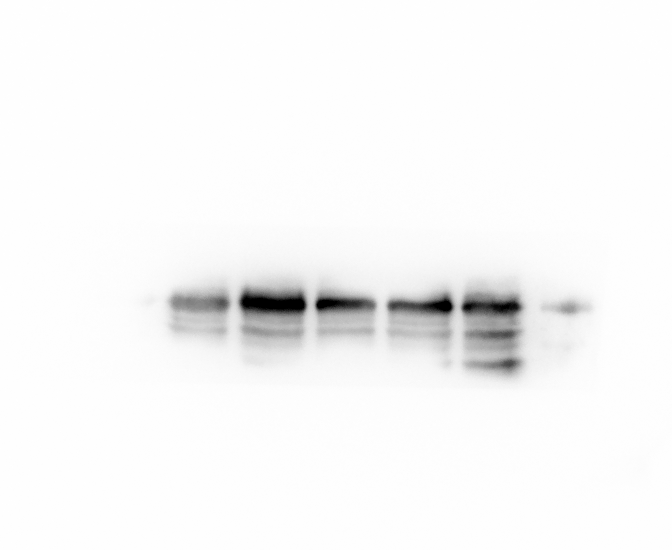

Supplement: Supplementary file 6 [file DataSheet2.ZIP › western blot/figure 7-A/P-P65-3.tif]

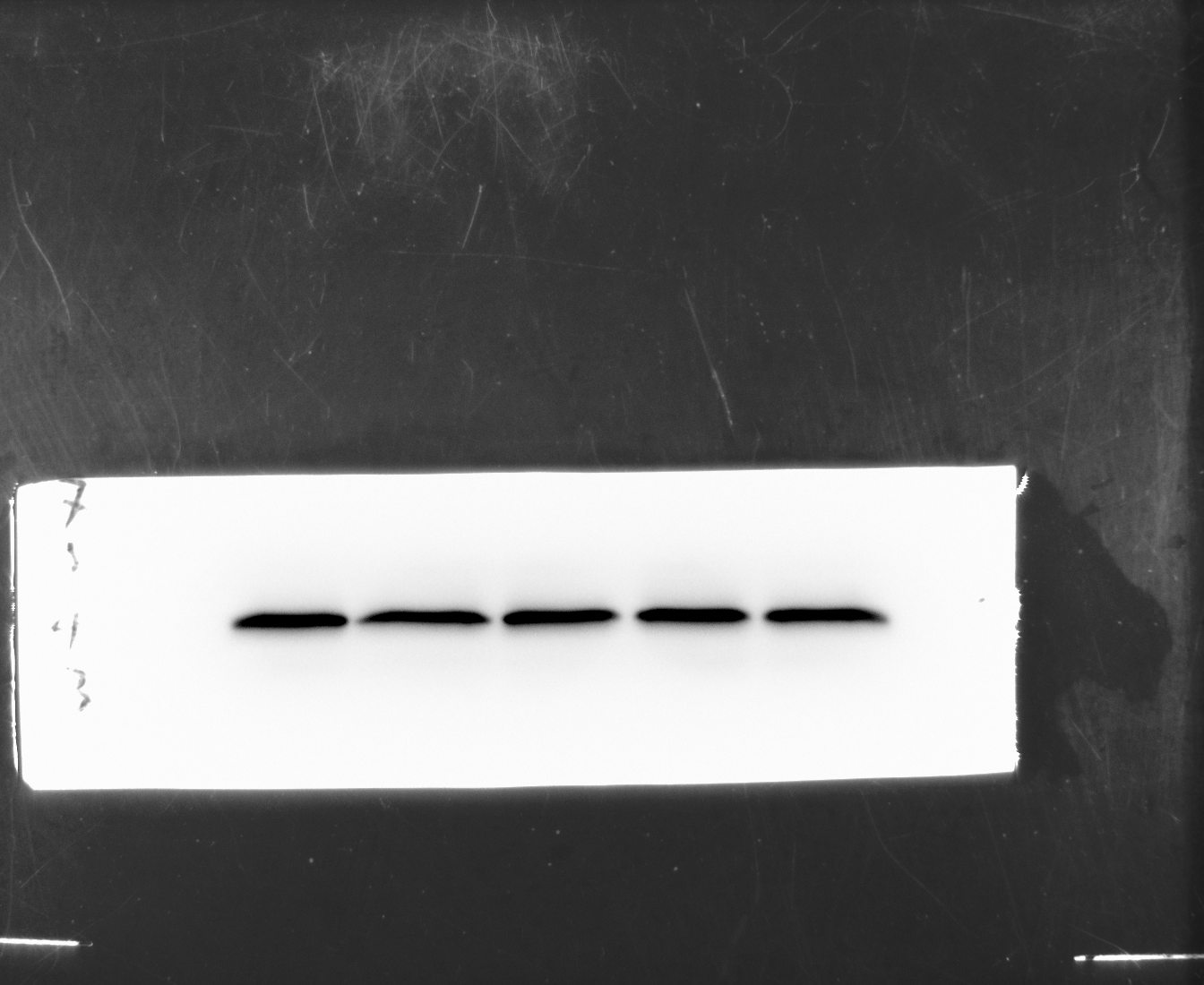

Supplement: Supplementary file 6 [file DataSheet2.ZIP › western blot/figure 7-A/P38-1.tif]

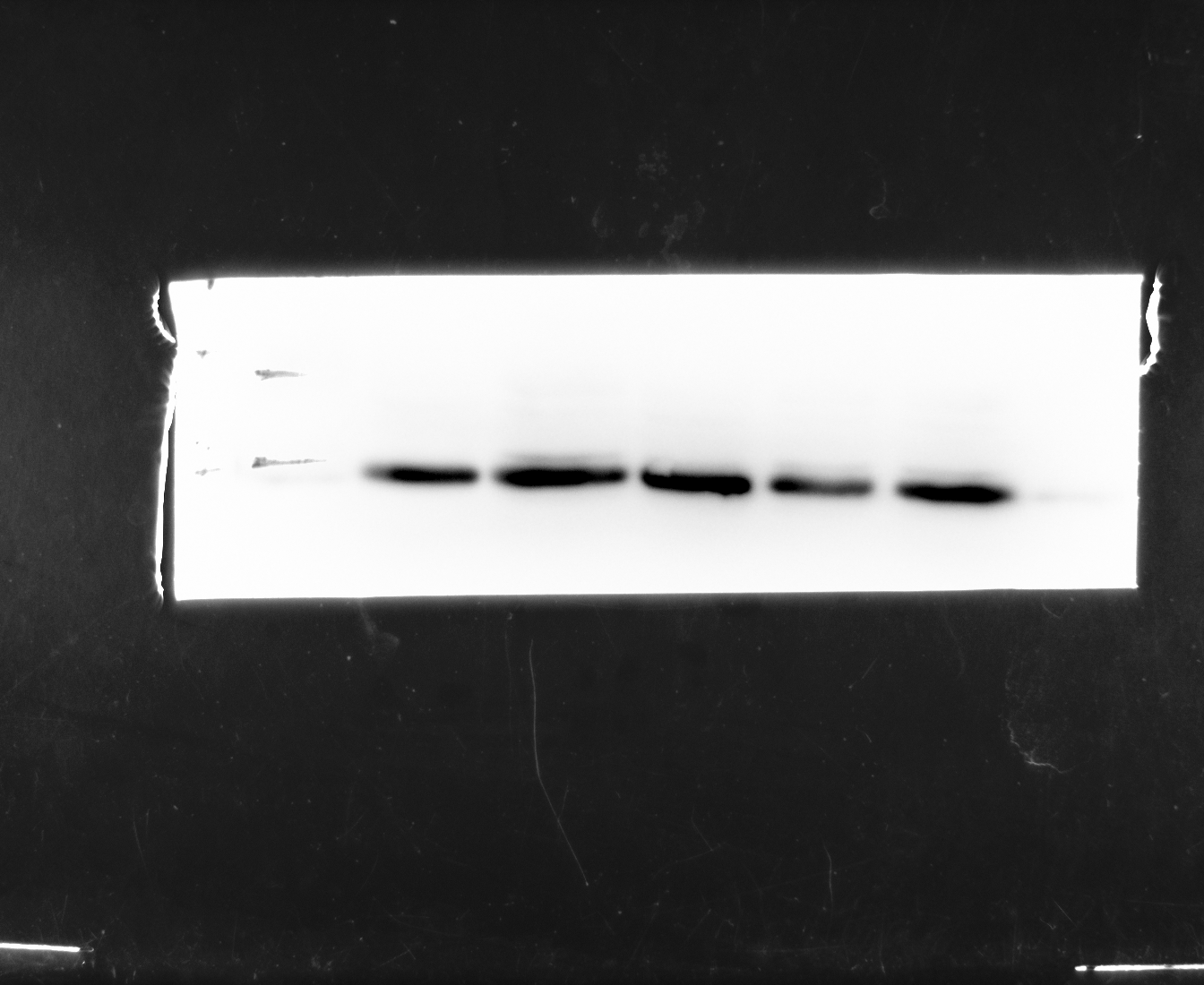

Supplement: Supplementary file 6 [file DataSheet2.ZIP › western blot/figure 7-A/P38-2.tif]
