# Supplementary material for: Guanxinkang Decoction Attenuates the Inflammation in Atherosclerosis by Regulating Efferocytosis and MAPKs Signaling Pathway in LDLR−/− Mice and RAW264.7 Cells
Source: Front Pharmacol. 2021 Dec 7;12:731769. doi: 10.3389/fphar.2021.731769 (PMC8688952; doi:10.3389/fphar.2021.731769)
Supplement: Supplementary file 7 [file DataSheet8.docx]

Supplementary Material

# Liquid Chromatography Mass Spectrometry (LCMS) experiment of GXK

GXK contains six herbs (Huang Qi, Yi Mu Cao, Dan Shen, Xie Bai, Ban Xia, Gua Lou).

## Supplementary Figure 1


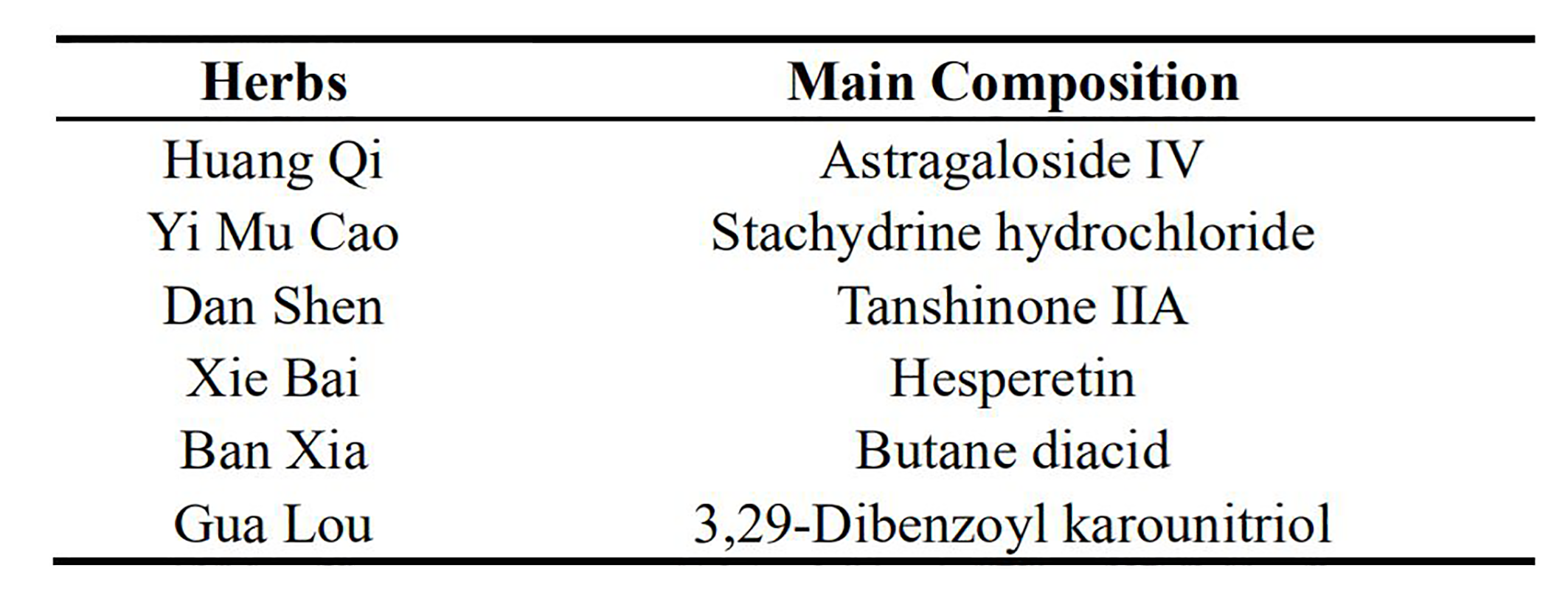


**Supplementary Figure 1.** Main composition of each herb according to the Chinese Pharmacopoeia.

## Supplementary Figure 2


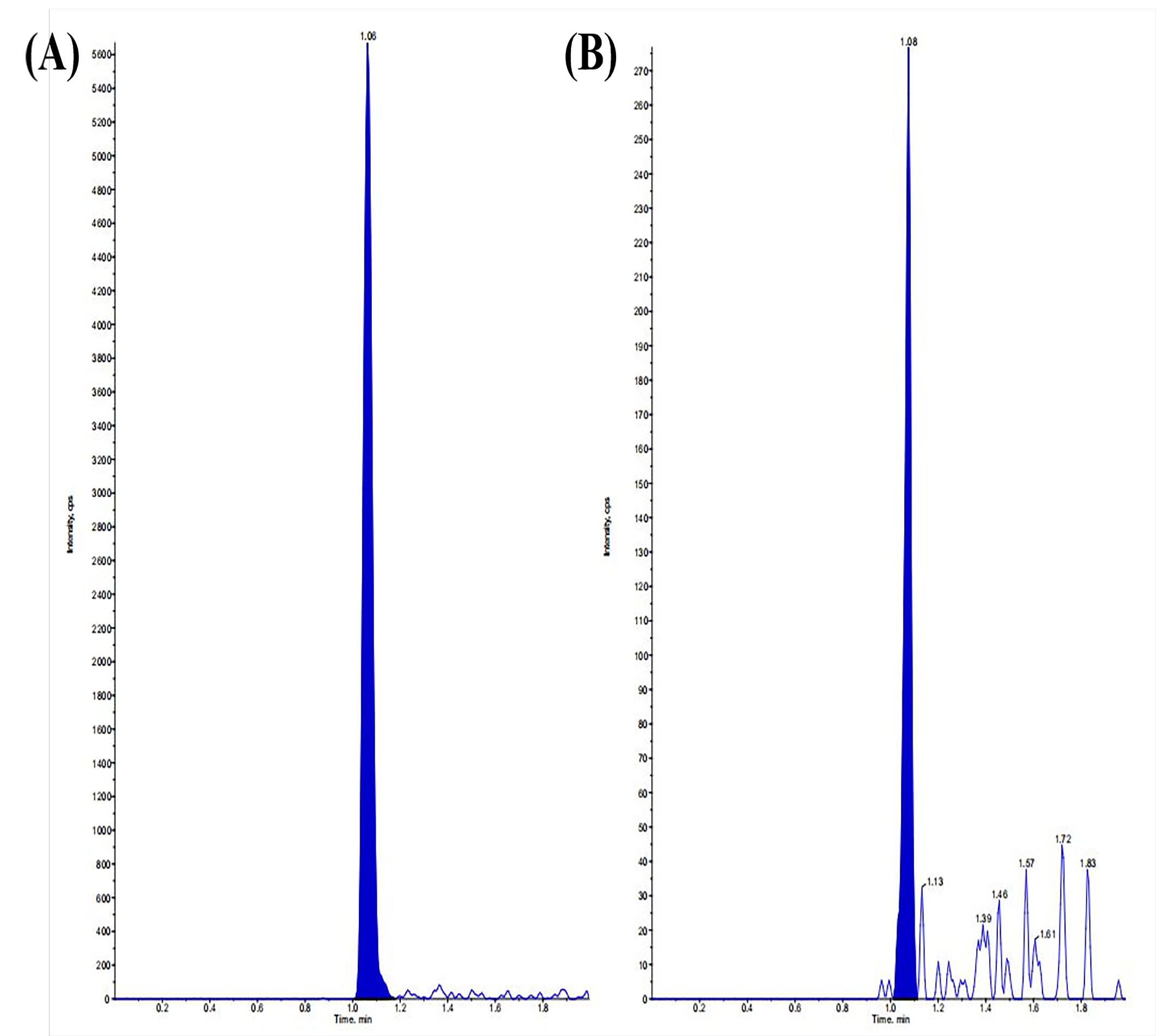


**Supplementary Figure 2.** LCMS of Astragaloside IV. (A) standard sample, (B) plant extract.

## Supplementary Figure 3


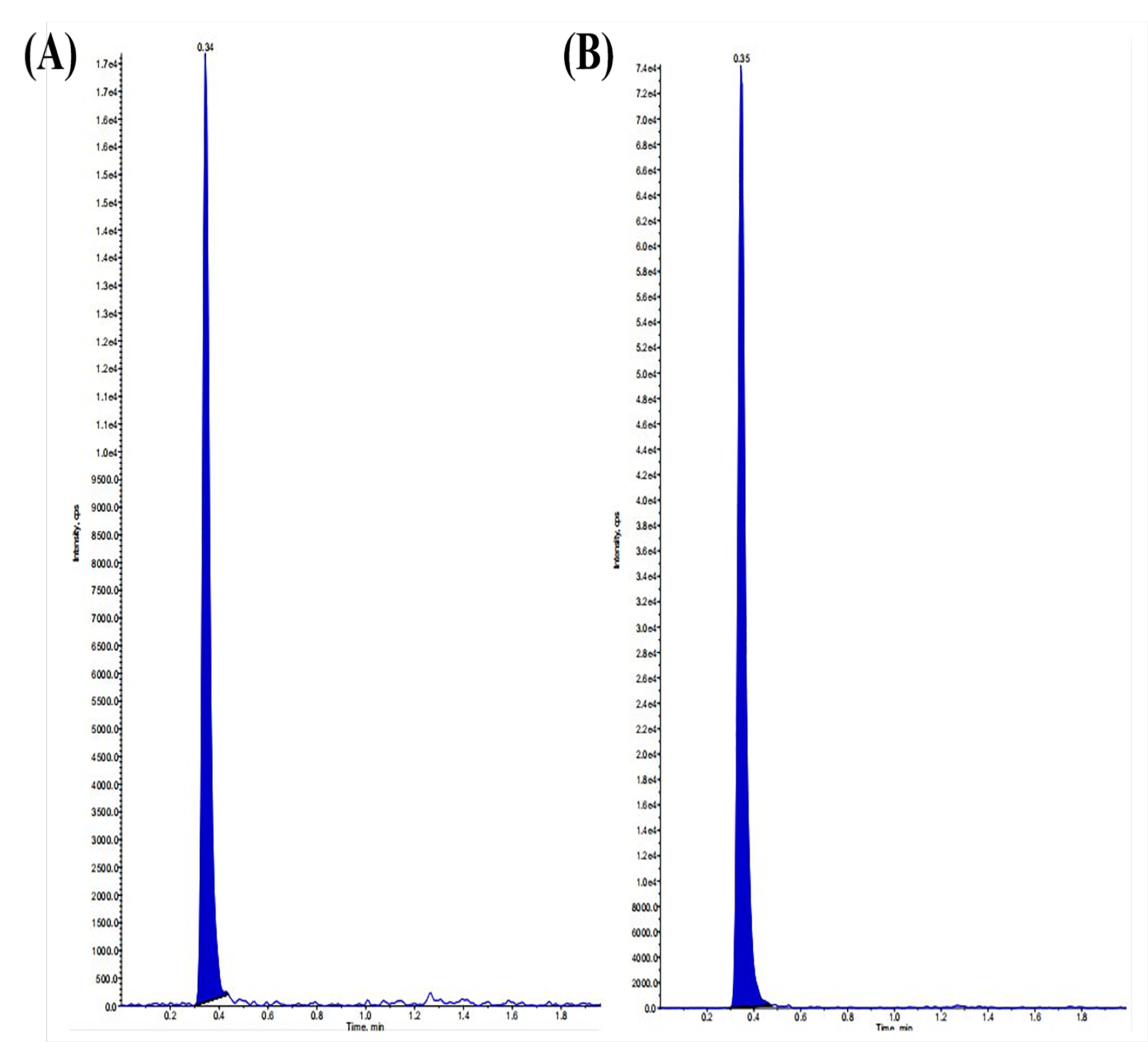


**Supplementary Figure 3.** LCMS of Stachydrine hydrochloride. (A) standard sample, (B) plant extract.

## Supplementary Figure 4


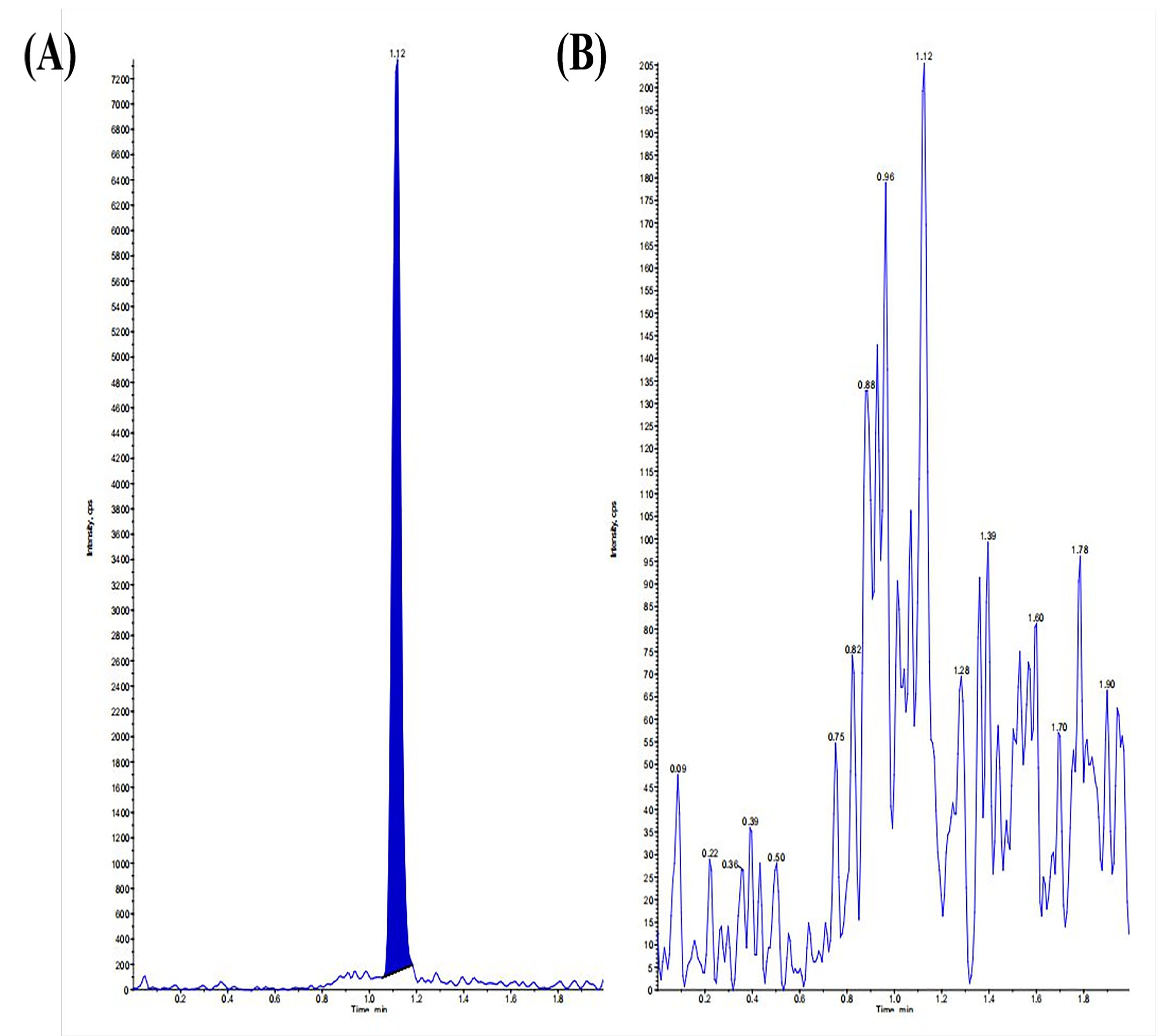


**Supplementary Figure 4.** LCMS of Tanshinone IIA. (A) standard sample, (B) plant extract.

## Supplementary Figure 5


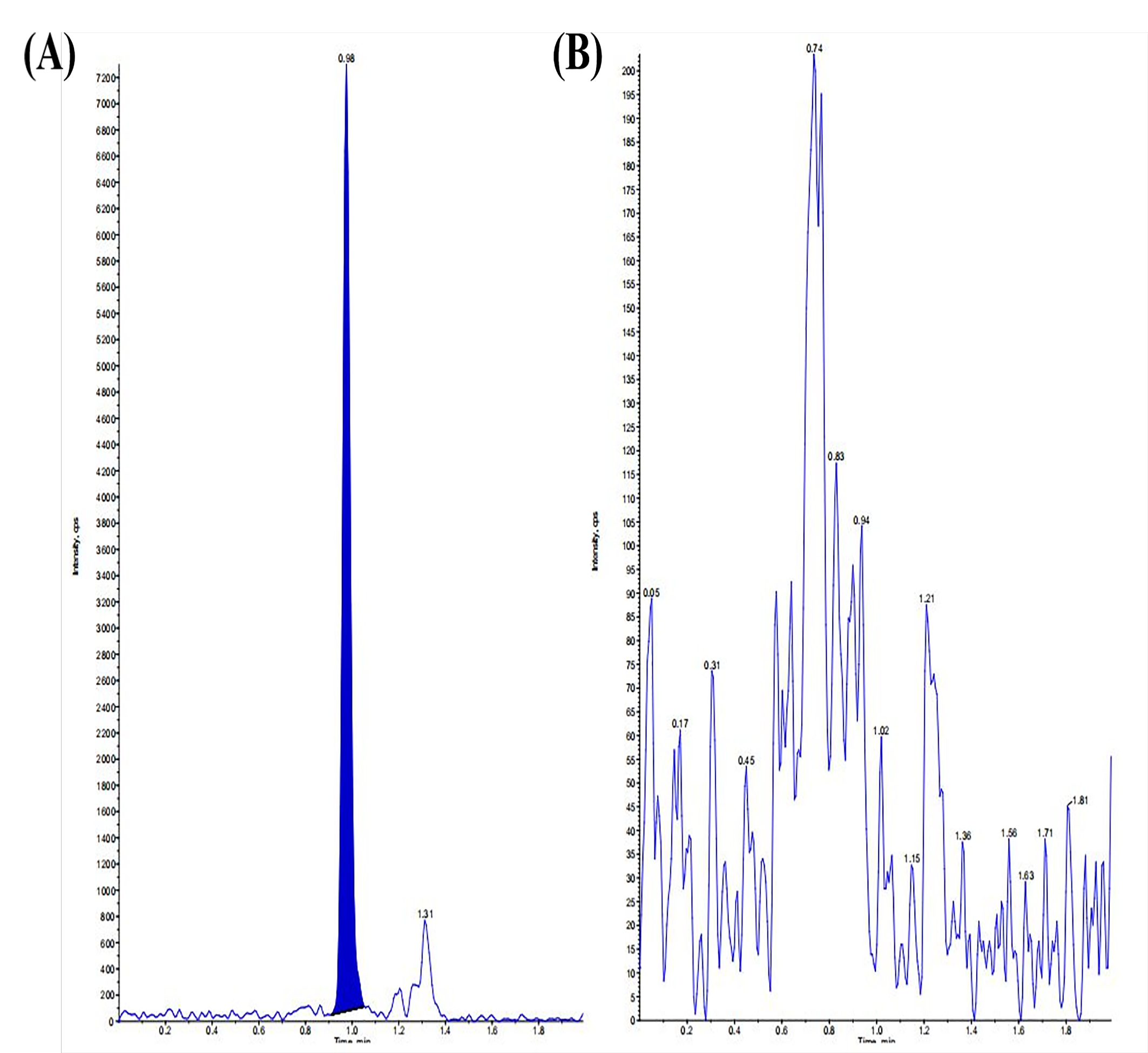


**Supplementary Figure 5.** LCMS of Hesperetin. (A) standard sample, (B) plant extract.

## Supplementary Figure 6


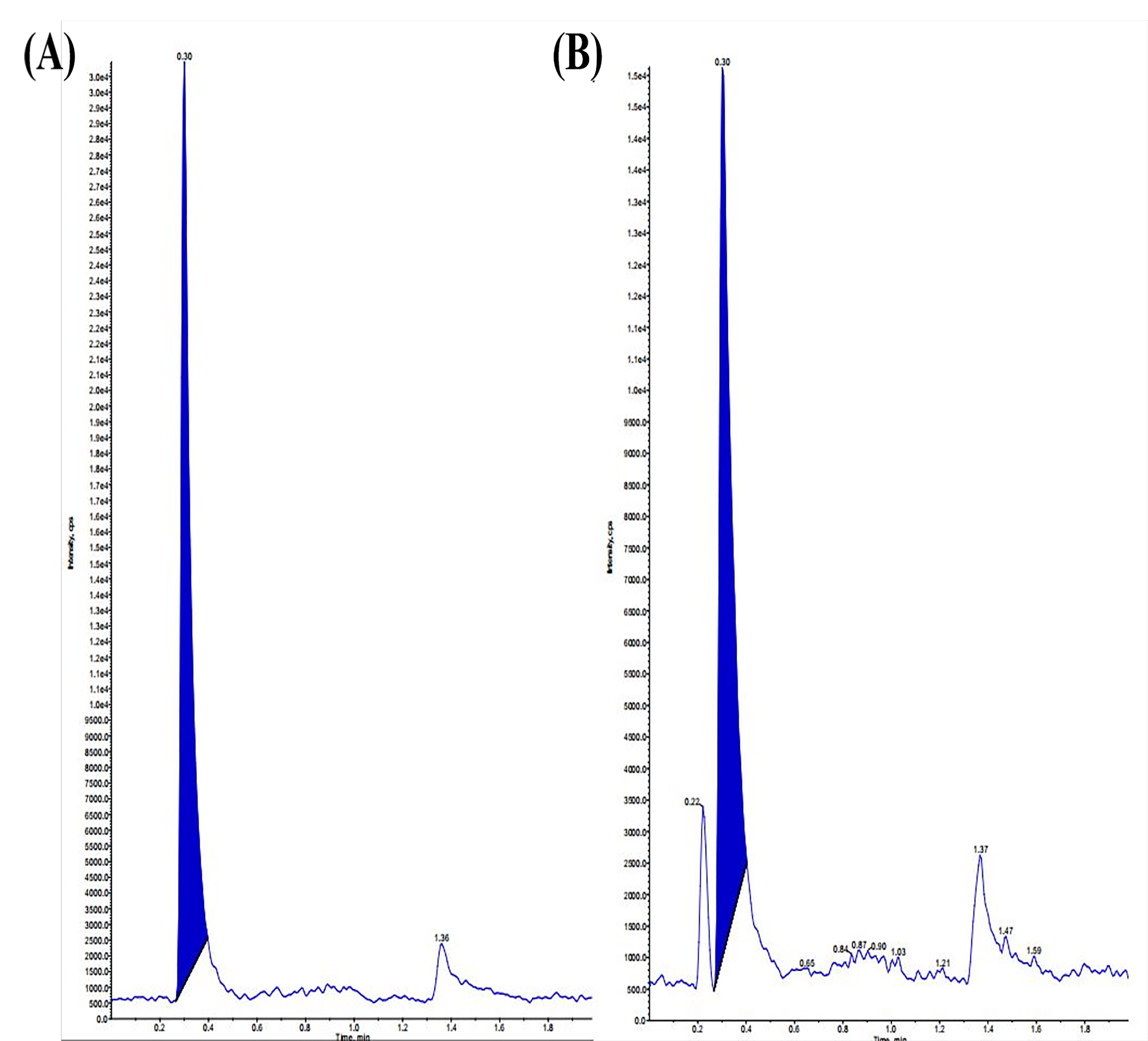


**Supplementary Figure 6.** LCMS of Butane diacid. (A) standard sample, (B) plant extract.

## Supplementary Figure 7


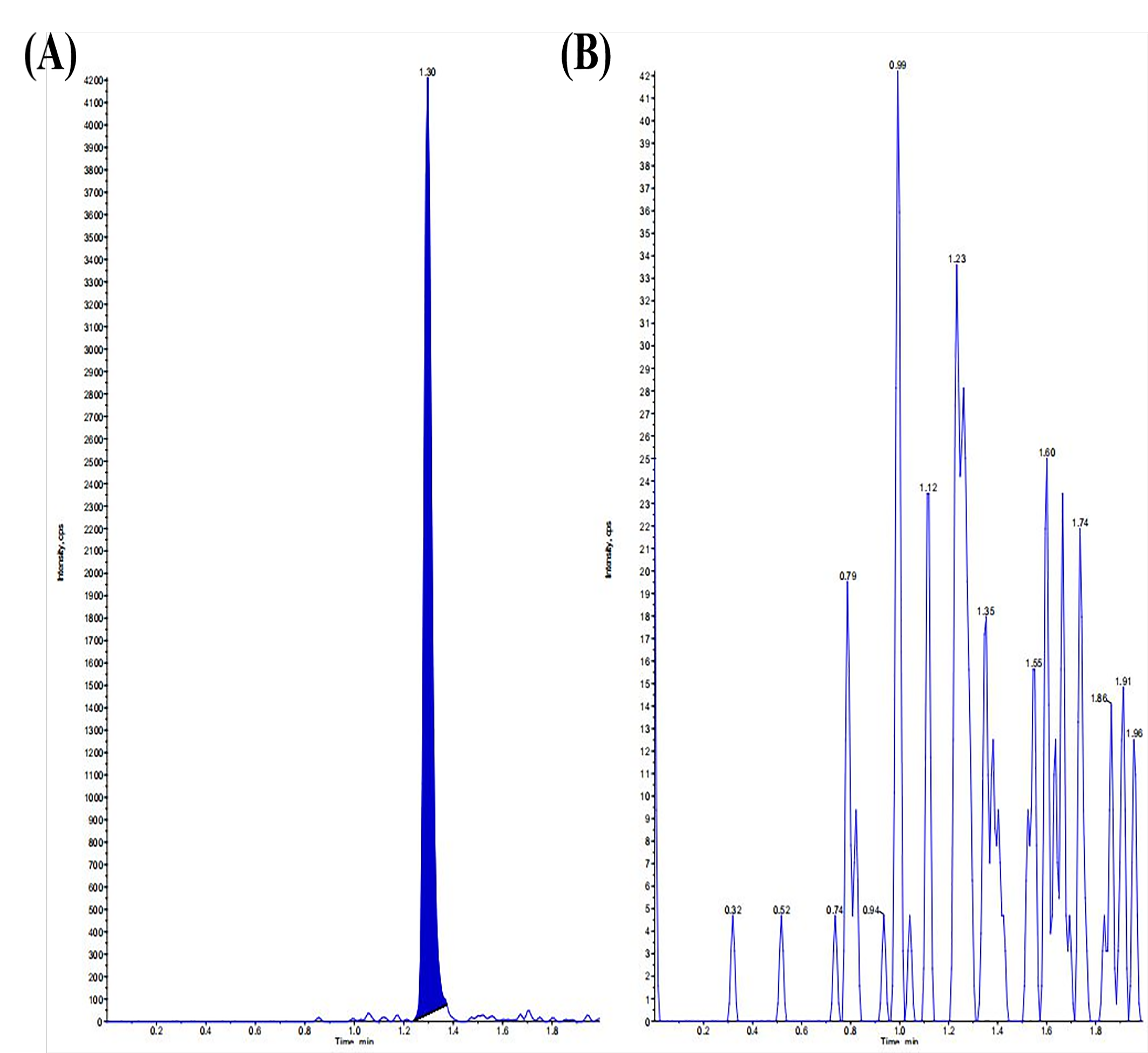


**Supplementary Figure 7.** LCMS of 3,29-Dibenzoyl karounitriol. (A) standard sample, (B) plant extract.

## Supplementary Figure 8


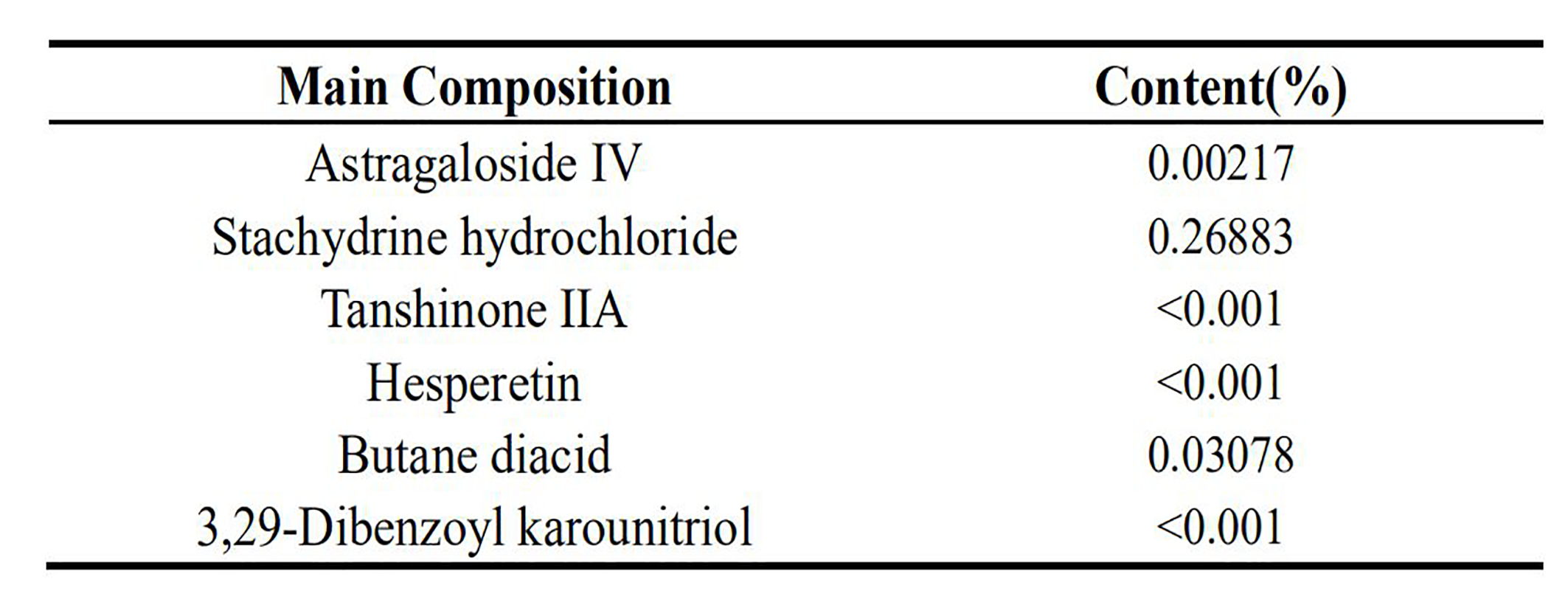


**Supplementary Figure 8.** Main composition of GXK based on the LCMS results.

# Animal welfare review material


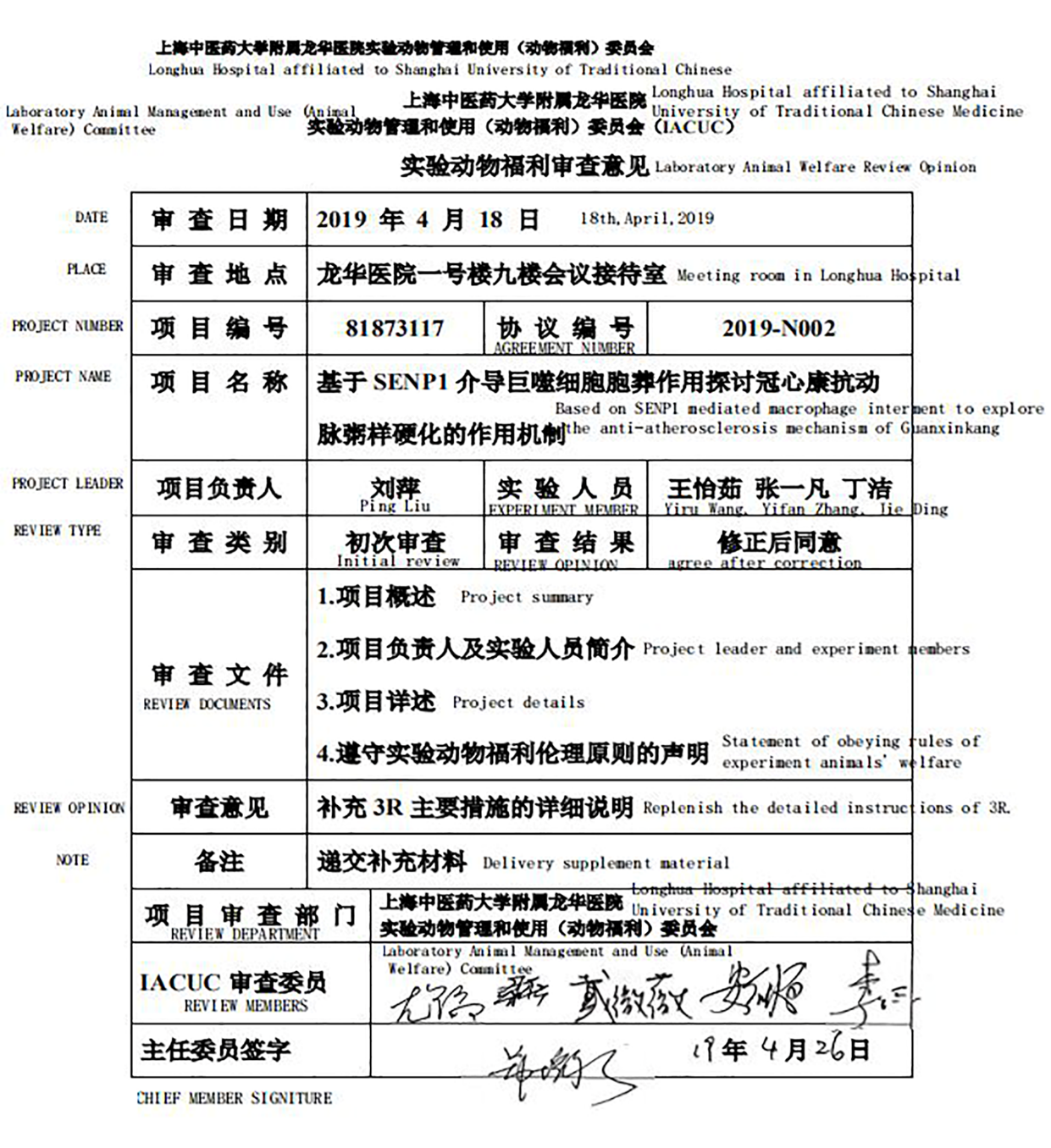


# GXK attenuates body weight in mice


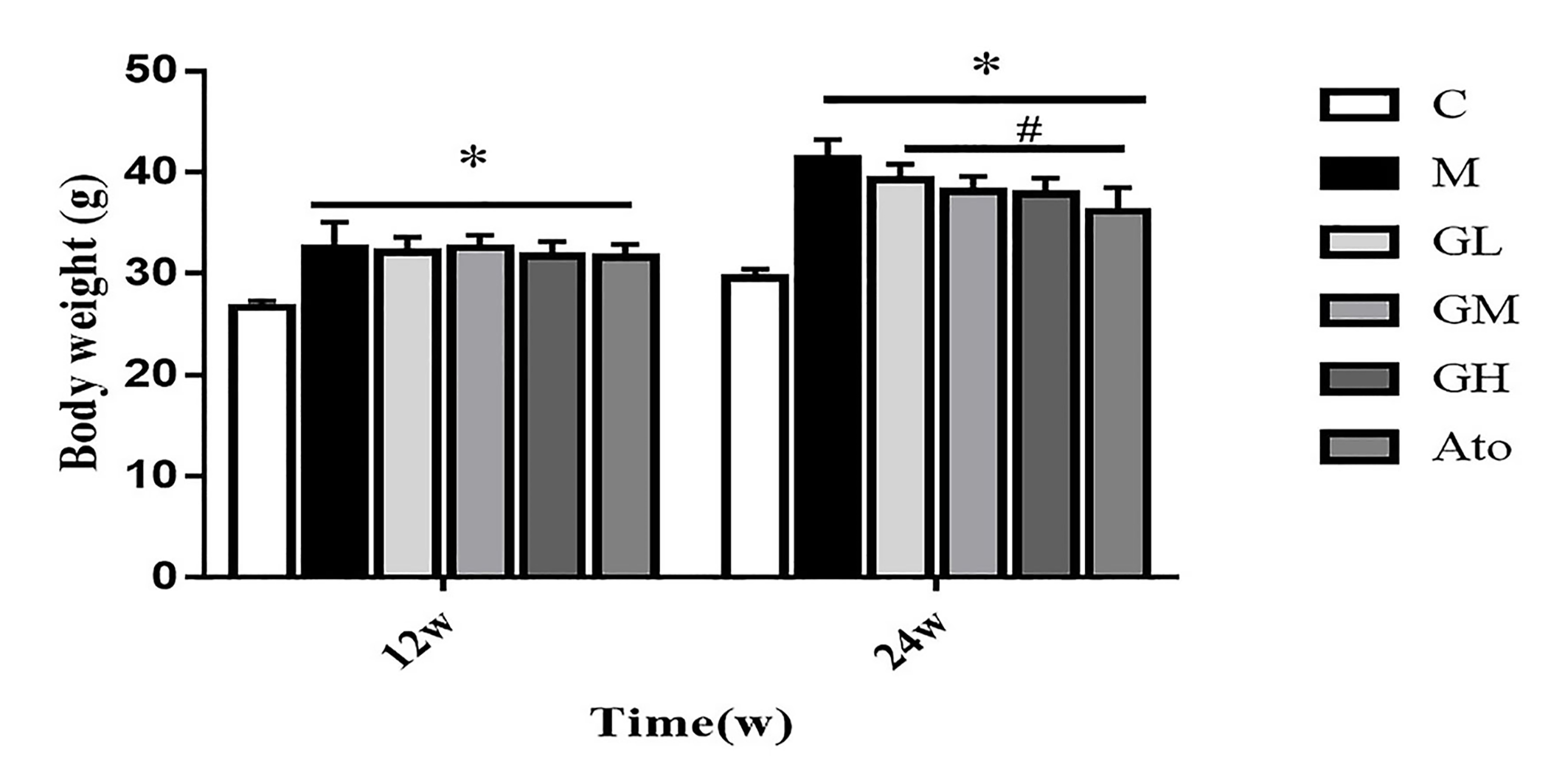


**Supplementary Figure 9.** GXK attenuates body weight in mice.

The body weight of mice was detected before the beginning and at the end of treatment, n=8. C means control group, M means model group, GL means low GXK group, GM means medium GXK group, GH means high GXK group, Ato means Atorvastatin group. Data are expressed as mean ± SD. **P* < 0.05 versus C group; # *P* < 0.05 versus M group.

# GXK attenuates liver enzymes levels in mice


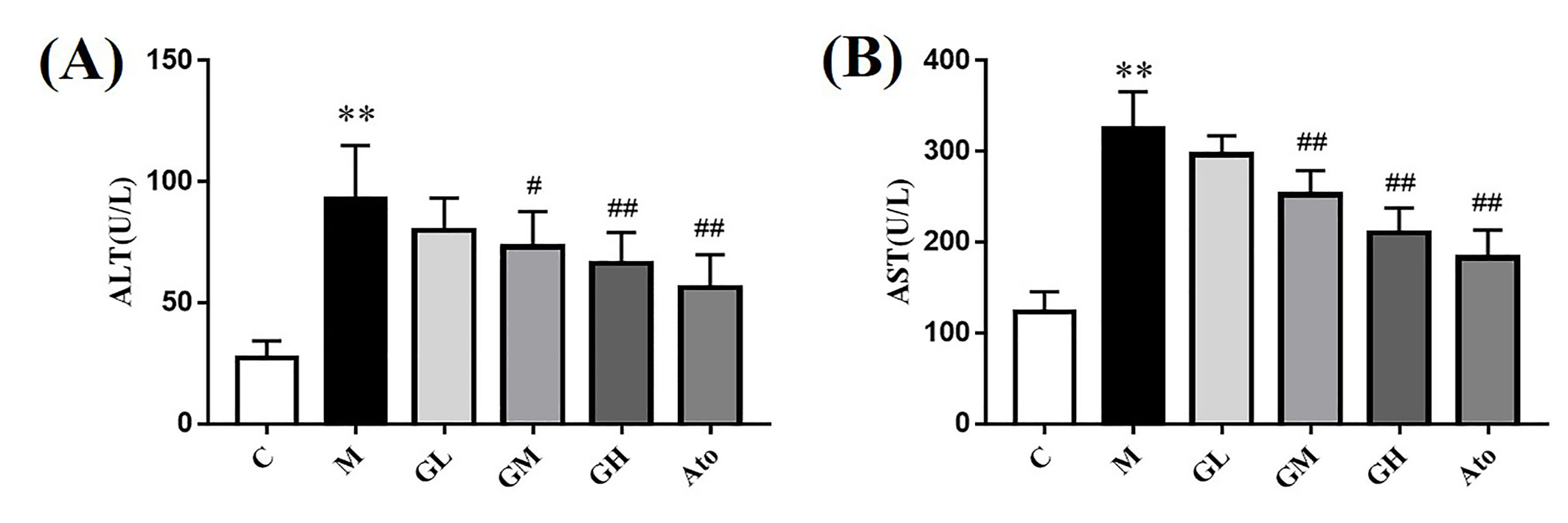


**Supplementary Figure 10.** GXK attenuates liver enzymes levels in mice.

(A-B) ALT andAST levels of serum were detected, n=8. C means control group, M means model group, GL means low GXK group, GM means medium GXK group, GH means high GXK group, Ato means Atorvastatin group. Data are expressed as mean ± SD. ***P* < 0.01 versus C group; # *P* < 0.05, # #*P* < 0.01 versus M group.
